# Automatic purpose-driven basis set truncation for time-dependent Hartree–Fock and density-functional theory

**DOI:** 10.1038/s41467-022-35694-4

**Published:** 2023-01-06

**Authors:** Ruocheng Han, Johann Mattiat, Sandra Luber

**Affiliations:** Department of Chemistry, University of Zurich, Zurich, Switzerland

**Keywords:** Quantum chemistry, Density functional theory

## Abstract

Real-time time-dependent density-functional theory (RT-TDDFT) and linear response time-dependent density-functional theory (LR-TDDFT) are two important approaches to simulate electronic spectra. However, the basis sets used in such calculations are usually the ones designed mainly for electronic ground state calculations. In this work, we propose a systematic and robust scheme to truncate the atomic orbital (AO) basis set employed in TDDFT and TD Hartree–Fock (TDHF) calculations. The truncated bases are tested for both LR- and RT-TDDFT as well as RT-TDHF approaches, and provide an acceleration up to an order of magnitude while the shifts of excitation energies of interest are generally within 0.2 eV. The procedure only requires one extra RT calculation with 1% of the total propagation time and a simple modification on basis set file, which allows an instant application in any quantum chemistry package supporting RT-/LR-TDDFT calculations. Aside from the reduced computational effort, this approach also offers valuable insight into the effect of different basis functions on computed electronic excitations and further ideas on the design of basis sets for special purposes.

## Abstract (web summary)

Time-dependent calculations are widely employed in simulating spectra. Here, the authors present a basis set truncation scheme to analyse and accelerate TDDFT calculations with negligible change in the resulting electronic spectra.

## Introduction

Electronically excited states and their properties are among the central topics of quantum chemistry research. The utilized theoretical methods for excited state calculations typically require equivalent or higher computational resources compared to analogous ground state calculations. Highly accurate multiconfigurational methods are computationally demanding and thus can only be applied to small systems.

Time-dependent density-functional theory (TDDFT), due to its good compromise between accuracy and efficiency, has been employed in a wide range of applications, especially for spectroscopy^1–3^. Real-time propagation (RTP) has become an appealing technique for the solution of the time-dependent Kohn–Sham calculations, namely, real-time time-dependent density-functional theory (RT-TDDFT), or general approximations to the time-dependent Schrödinger equation^4–9^. It is based on the evolution of molecular orbitals under the influence of an external field, often with only a *δ*-pulse (field) applied at the beginning. In the weak field limit within the adiabatic approximation, the spectroscopy simulations using RT-TDDFT and linear response time-dependent density-functional theory (LR-TDDFT) should provide comparable results^4^. During each time step, one needs to construct the Hamiltonian given by the new molecular orbital (MO) coefficients, which is the most time-consuming part of the RTP. Depending on the quantum chemistry method employed in RTP, the construction of the Hamiltonian may scale to ${{{{{{{\mathcal{O}}}}}}}}({N}^{4})$, e.g., for HF Coulomb and exchange matrices calculated with 2-electron integrals, where *N* refers to the number of AO basis functions. Therefore, reducing the number of basis functions or finding a proper smaller basis set can potentially save a large amount of computational time and memory.

Previous studies on the topic of basis set truncation/reduction have mainly followed three strategies: (1) decreasing the size of the virtual space for frozen natural orbital approximations used in perturbation based methods^10,11^ (e.g., Møller–Plesset perturbation theory, coupled cluster single-double and perturbative triple, complete active space perturbation theory), (2) reducing the number of functions in correlation consistent basis sets^12,13^, (3) reducing the number of basis functions of subsystems (which apply expensive wavefunction-based methods) for embedding calculations^14,15^. However, these works focus on the electronic ground state. Multiple embedding techniques have been applied to accelerate RT-TDDFT calculations by treating subsystems with different level of theories^16–20^. The idea of a decomposition of the electric dipole moment into molecular orbital pairs was also proposed in recent works for the acceleration or analyses of spectra^21–23^. This work explores the contribution of a fundamental ingredient—basis functions—to the electronic spectra. The truncation of basis functions proposed in this work is designed to check every single component in the basis set (basis function). One can also apply a shell level truncation for general applications. Basis set files can be easily modified for an accelerated simulation of the spectrum and to obtain a better chemical insight into the electric dipole moments contribution. Moreover, a routine to construct complete basis set (CBS) for TDDFT calculations is proposed. The calculations of electronic absorption and ECD spectra in this work take place in a linear response framework within the electric dipole approximation, assuming that the excited states of the system can be well described within the occupied-virtual space spanned by the ground state solution of the system. As standardly done, we assume the adiabatic approximation, discarding the dependence of the exchange-correlation functional on the history of the propagation. The decomposition of electric dipole moments into the contribution of individual AO basis functions and checking the variation of molecular orbitals (in component of basis functions) during the RTP provides a quantitative evaluation of each AO basis function based on its importance for the electronic spectra under study. This further paves the way for a truncation process on the basis set for the computational speed-up and a way to generate complete basis set for TDDFT calculations.

In this work, we propose a basis set truncation scheme for TDDFT calculations. The method is tested for small molecules up to a highly conjugated system and a metal cluster, and achieves an acceleration up to an order of magnitude in RT-TDDFT or LR-TDDFT calculations with negligible change in the region of interests (e.g., valence-shell transitions) of the computed spectra.

## Results

### Electric dipole moment

In the context of this work, the electronic part of the electric dipole moment $\overrightarrow{d}$ is defined as the trace of the product of the density matrix and integrals of the electric dipole moment operator $-e\overrightarrow{r}$ ($\overrightarrow{r}=(x,\, y,\, z)$, *e* is elementary charge) in the AO basis with basis functions $\{{\chi }_{\mu }\}$. For calculating the time-dependent electric dipole moment $\overrightarrow{d}(t)$, we use the AO basis representation for both density matrix ***P***^AO^(*t*) and the electric dipole moment integrals $\overrightarrow{{{{{{{{\boldsymbol{D}}}}}}}}}$ as shown in Eq. (1). In this way, only the density matrix ***P***^AO^(*t*) is time-dependent and $\overrightarrow{{{{{{{{\boldsymbol{D}}}}}}}}}$ remains the same during the RTP for fixed nuclei. ***P***^AO^(*t*) can be further expressed in molecular orbital (MO) basis as ***P***^MO^ (MO density matrix after SCF, see Eq. (3) where *f*_*i*_ is the occupation number of the *i*th MO) and the time-dependent part is only carried by the MO coefficients ***C***(*t*) and its complex conjugate ***C***^†^(*t*) (see Eq. (2)). In this work, the AO basis functions are all Gaussian-type orbitals.

1 $$\overrightarrow{d}(t)=-\!e\cdot \mathop{\sum}\limits_{\mu \nu }{P}_{\mu \nu }^{{{{{{{{\rm{AO}}}}}}}}}\langle {\chi }_{\mu }|\overrightarrow{r}|{\chi }_{\nu }\rangle=-\!e\cdot {{{{{{{\rm{Tr}}}}}}}}({{{{{{{{\boldsymbol{P}}}}}}}}}^{{{{{{{{\rm{AO}}}}}}}}}(t)\overrightarrow{{{{{{{{\boldsymbol{D}}}}}}}}})$$

2 $${{{{{{{{\boldsymbol{P}}}}}}}}}^{{{{{{{{\rm{AO}}}}}}}}}(t)={{{{{{{\boldsymbol{C}}}}}}}}(t){{{{{{{{\boldsymbol{P}}}}}}}}}^{{{{{{{{\rm{MO}}}}}}}}}{{{{{{{{\boldsymbol{C}}}}}}}}}^{{{{\dagger}}} }(t)$$

3 $${{{{{{{{\boldsymbol{P}}}}}}}}}^{{{{{{{{\rm{MO}}}}}}}}}=({p}_{ij})=\left\{\begin{array}{ll}{f}_{i},\quad &i=j\\ 0,\quad &i\,\ne\, j\end{array}\right.$$

### Real-time propagation

In our implementation, the MO coefficients ***C***(*t*) are propagated for a small timestep Δ*t* (see Eq. (4)) using the “enforced time-reversal symmetry” (ETRS)^24^ scheme. ***U***(*t* + Δ*t*) represents the propagator at time *t* + Δ*t* and is calculated with Eq. (5), where ***S*** is the overlap matrix in AO basis and ***F***(*t*) is the Fock matrix or Kohn–Sham (KS) matrix in AO basis at time *t*. ***C***(*t* + Δ*t*), ***U***(*t* + Δ*t*), and ***F***(*t* + Δ*t*) are computed self-consistently^24^.

4 $${{{{{{{\boldsymbol{C}}}}}}}}(t+{{\Delta }}t)={{{{{{{\boldsymbol{U}}}}}}}}(t+{{\Delta }}t){{{{{{{\boldsymbol{C}}}}}}}}(t)$$

5 $${{{{{{{\boldsymbol{U}}}}}}}}(t+{{\Delta }}t)=\exp \left[-\frac{i}{2}{{{{{{{{\boldsymbol{S}}}}}}}}}^{-1}({{{{{{{\boldsymbol{F}}}}}}}}(t)+{{{{{{{\boldsymbol{F}}}}}}}}(t+{{\Delta }}t)){{\Delta }}t\right]$$

***F***(*t*) needs to be constructed for each time step and usually contributes most to the computational time in RTP. For example, the elements of the HF exchange matrix *K*_*μν*_(*t*) are given in Eq. (6) ($\left\langle \mu \lambda|\sigma \nu \right\rangle$ are the two-electron repulsion integral (ERIs) in AO basis expressed in Eq. (7)), and the computation of the exchange matrix ***K***(*t*), which is required for the construction of ***F***(*t*), scales as ${{{{{{{\mathcal{O}}}}}}}}({{N}_{{{{{{{{\rm{AO}}}}}}}}}}^{4})$ (*N*_AO_ is the number of AO basis functions).

6 $${K}_{\mu \nu }(t)=\mathop{\sum}\limits_{\lambda \sigma }{P}_{\lambda \sigma }^{{{{{{{{\rm{AO}}}}}}}}}(t)\left\langle \mu \sigma|\lambda \nu \right\rangle$$

7 $$\left\langle \mu \sigma|\lambda \nu \right\rangle=\int{\chi }_{\mu }^{*}({\overrightarrow{r}}_{1}){\chi }_{\sigma }^{*}({\overrightarrow{r}}_{2})\frac{1}{{r}_{12}}{\chi }_{\lambda }({\overrightarrow{r}}_{1}){\chi }_{\nu }({\overrightarrow{r}}_{2})d{\overrightarrow{r}}_{1}d{\overrightarrow{r}}_{2}$$

### AO basis truncation

In order to decrease *N*_AO_, we first analyse Eq. (1) for the electric dipole contribution from each AO basis function. For the sake of simplicity, ${\overrightarrow{O}}_{\mu }(t)$ is used to represent the *μ*th diagonal element of ${{{{{{{{\boldsymbol{P}}}}}}}}}^{{{{{{{{\rm{AO}}}}}}}}}(t)\overrightarrow{{{{{{{{\boldsymbol{D}}}}}}}}}$, and thus $\overrightarrow{d}(t)$ can then be rewritten as in Eq. (8). Taking a detailed look at the construction of ${\overrightarrow{O}}_{\mu }(t)$ in Eq. (9), one can find that it provides a decomposed form of electric dipole moments of each basis function. Therefore, we use ${\overrightarrow{O}}_{\mu }(t)$ to represent the electric dipole contribution from the *μ*th basis function.

8 $$\overrightarrow{d}(t)=-\!e\cdot \mathop{\sum }\limits_{\mu }^{{N}_{{{{{{{{\rm{AO}}}}}}}}}}{\overrightarrow{O}}_{\mu }(t),\,{{{{{{{\rm{where}}}}}}}}\,{\overrightarrow{O}}_{\mu }(t)={({{{{{{{{\boldsymbol{P}}}}}}}}}^{{{{{{{{\rm{AO}}}}}}}}}(t)\overrightarrow{{{{{{{{\boldsymbol{D}}}}}}}}})}_{\mu \mu }$$

9 $${\overrightarrow{O}}_{\mu }(t)=\mathop{\sum}\limits_{\nu }{P}_{\mu \nu }^{{{{{{{{\rm{AO}}}}}}}}}(t)\langle {\chi }_{\mu }|\overrightarrow{r}-\overrightarrow{R}|{\chi }_{\nu }\rangle$$

However, ${\overrightarrow{O}}_{\mu }(t)$ is not translational invariant because the value of ${\overrightarrow{D}}_{\mu \nu }$ (element in $\overrightarrow{{{{{{{{\boldsymbol{D}}}}}}}}}$) depends on the choice of reference points $\overrightarrow{R}$ (see Eq. (10)). Note that $\overrightarrow{r}$ and $\overrightarrow{R}$ are referenced to the origin of coordinate system. Though $\overrightarrow{R}$ does not affect the full spectrum after Fourier transform (because $\overrightarrow{d}$ is translational invariant for neutral systems as $\overrightarrow{R}{S}_{\mu \nu }$ cancels with the nuclear electric dipole contribution), it can change the relative contribution of electric dipole moments from each AO basis function (${\overrightarrow{O}}_{\mu }(t)$). We can further split ${\overrightarrow{D}}_{\mu \nu }$ into a reference point ($\overrightarrow{R}$)-independent term $\langle {\chi }_{\mu }|\overrightarrow{r}|{\chi }_{\nu }\rangle$ and a reference point-dependent term $\overrightarrow{R}{S}_{\mu \nu }$, where *S*_*μν*_ is the element of the overlap matrix in AO basis.

10 $${\overrightarrow{D}}_{\mu \nu }=\langle {\chi }_{\mu }|\overrightarrow{r}-\overrightarrow{R}|{\chi }_{\nu }\rangle=\langle {\chi }_{\mu }|\overrightarrow{r}|{\chi }_{\nu }\rangle -\overrightarrow{R}{S}_{\mu \nu },\,{{{{{{{\rm{where}}}}}}}}\,{S}_{\mu \nu }=\left\langle {\chi }_{\mu }|{\chi }_{\nu }\right\rangle$$

In $\langle {\chi }_{\mu }|\overrightarrow{r}|{\chi }_{\nu }\rangle$, the relative position of atoms can cause different values of elements in the matrix, which we would like to avoid. To explain the reason, we can think about a toy system consisting of only two hydrogen atoms with Cartesian coordinates H1 ${\overrightarrow{r}}_{1}$ = (0, 0, -*a*) and H2 ${\overrightarrow{r}}_{2}$ = (0, 0, *a*) where *a* ≠ 0. It is obvious that e.g., diagonal matrix elements $\langle {\chi }_{{{{{{{{{\rm{H1}}}}}}}}}_{s}}|\overrightarrow{r}|{\chi }_{{{{{{{{{\rm{H1}}}}}}}}}_{s}}\rangle \,\ne \,\langle {\chi }_{{{{{{{{{\rm{H2}}}}}}}}}_{s}}|\overrightarrow{r}|{\chi }_{{{{{{{{{\rm{H2}}}}}}}}}_{s}}\rangle$ (see Eq. (11)) even though, by symmetry, we expect the same “contribution” of electric dipole from the two atoms. In Eq. (11), each H atom has a Slater-type 1*s* orbital *Ae*^−*ζr*^ where *A* is normalization constant and *r* is the distance from the center of the atom, and we change the integration variable from $\overrightarrow{r}$ to $\overrightarrow{s}$ using $\overrightarrow{s}=\overrightarrow{r}-{\overrightarrow{r}}_{1}$ and $\overrightarrow{s}=\overrightarrow{r}-{\overrightarrow{r}}_{2}$.

11 $$\langle {\chi }_{{{{{{{{{\rm{H2}}}}}}}}}_{s}}|\overrightarrow{r}|{\chi }_{{{{{{{{{\rm{H2}}}}}}}}}_{s}}\rangle -\langle {\chi }_{{{{{{{{{\rm{H1}}}}}}}}}_{s}}|\overrightarrow{r}|{\chi }_{{{{{{{{{\rm{H1}}}}}}}}}_{s}}\rangle	=\int{A}^{2}\overrightarrow{r}{e}^{-2\zeta|\overrightarrow{r}-{\overrightarrow{r}}_{2}|}d\overrightarrow{r}-\int{A}^{2}\overrightarrow{r}{e}^{-2\zeta|\overrightarrow{r}-{\overrightarrow{r}}_{1}|}d\overrightarrow{r}\\ 	=\int{A}^{2}(\overrightarrow{s}+{\overrightarrow{r}}_{2}){e}^{-2\zeta|\overrightarrow{s}|}d\overrightarrow{s}-\int{A}^{2}(\overrightarrow{s}+{\overrightarrow{r}}_{1}){e}^{-2\zeta|\overrightarrow{s}|}d\overrightarrow{s}\\ 	=({\overrightarrow{r}}_{2}-{\overrightarrow{r}}_{1})\int{A}^{2}{e}^{-2\zeta|\overrightarrow{s}|}d\overrightarrow{s}\\ 	={\overrightarrow{r}}_{2}-{\overrightarrow{r}}_{1}$$

One way of minimizing the effect of $\langle {\chi }_{\mu }|\overrightarrow{r}|{\chi }_{\nu }\rangle$ is to shift the molecular system far from (0, 0, 0), which is equivalent to set a large $\overrightarrow{R}$. It is worth noting that we do not need to formally “move” the molecule, and this is just an assumption made in the derivation from Eq. (9) to Eq. (12). In this method, we only care about the relative value of ${\overrightarrow{O}}_{\mu }(t)$ when determining the basis function(s) to be truncated, and $\overrightarrow{R}$ provides the same factor to ${\overrightarrow{D}}_{\mu \nu }$ and later ${\overrightarrow{O}}_{\mu }(t)$. Therefore, it is safe to substitute ${\overrightarrow{D}}_{\mu \nu }$ with *S*_*μν*_ in the expression of ${\overrightarrow{O}}_{\mu }(t)$, and thus we have a scalar *O*_*μ*_(*t*) as shown in Eq. (12). It is worth noting that *O*_*μ*_(*t*) does not explicitly contain any electric dipole information, which makes sense because the electric dipole cannot be formally defined on a single atomic centered orbital. However, AOs do contribute to the electric dipole by forming MOs across different atomic centers, and the information of such contribution, which we call density matrix contribution, is contained in ***P***^AO^(*t*). The expression in Eq. (12) is also known in *Mulliken* population analysis, as the number of electrons associated with ${\chi }_{\mu }$. The following computations are all based on the scalar form *O*_*μ*_(*t*).

12 $${O}_{\mu }(t)={({{{{{{{{\boldsymbol{P}}}}}}}}}^{{{{{{{{\rm{AO}}}}}}}}}(t){{{{{{{\boldsymbol{S}}}}}}}})}_{\mu \mu }$$

To quantify the contribution, we use the formula in Eq. (13). ${x}_{\mu }^{{{{{{{{\rm{DC}}}}}}}}}$ is an indicator measuring the variation of the Density matrix Contribution (DC) of the *μ*th AO basis function. *S*_*t*_[*O*_*μ*_(*t*)] computes the standard deviation of *O*_*μ*_(*t*) for the total simulation time for each *μ*. The numerator of Eq. (13) indicates variation (along the RTP) of the electric dipole moment contribution from each AO basis function. The dimensionless quantity ${x}_{\mu }^{{{{{{{{\rm{DC}}}}}}}}}$ is then constructed by dividing the numerator with its mean value of all AO basis functions. A small ${x}_{\mu }^{{{{{{{{\rm{DC}}}}}}}}}$ value means the change of electric dipole moments contributed from the *μ*th basis function is comparatively small among all AO basis functions, and removing this basis function should not change the spectrum (a constant value vanishes after Fourier transform for RT-TDHF/TDDFT) significantly. One might point out that ${x}_{\mu }^{{{{{{{{\rm{DC}}}}}}}}}$ cannot distinguish the pulse from different directions because *O*_*μ*_(*t*) in Eq. (12) is no longer direction dependent like in Eq. (8). However, it is found that ***P***^AO^(*t*) still varies according to the direction of the pulse because its action is coded in the MO coefficients.

13 $${x}_{\mu }^{{{{{{{{\rm{DC}}}}}}}}}=\frac{{S}_{t}[{O}_{\mu }(t)]}{\frac{1}{{N}_{{{{{{{{\rm{AO}}}}}}}}}}{\sum }_{\mu }^{{N}_{{{{{{{{\rm{AO}}}}}}}}}}{S}_{t}[{O}_{\mu }(t)]}$$

It is worth noting that we have considered applying basis transformation regarding to ${{{{{{{{\boldsymbol{P}}}}}}}}}^{{{{{{{{\rm{AO}}}}}}}}}(t)\overrightarrow{{{{{{{{\boldsymbol{D}}}}}}}}}$, namely using the eigenvectors of ***P***^AO^(*t*) (transform to natural orbital basis) or $\overrightarrow{{{{{{{{\boldsymbol{D}}}}}}}}}$. However, the former one is time-dependent so it is hard to choose a transformation matrix for all time steps, and the latter one is reference point-dependent (see Eq. (10)) and one cannot obtain the consistent truncation choice under translation (note that nuclei do not move in this study as opposed to, e.g., Ehrenfest dynamics). Also, an AO basis is a common choice in most molecular simulations and some solid state simulations (e.g., Gaussian and Plane Waves^25^ method in CP2K package (CP2K version 7.0 (Development Version), the CP2K developers group. CP2K is freely available from https://www.cp2k.org/.)). Therefore, the truncation on the AO basis has broad application prospects and can be easily applied by a simple modification of basis set file.

During practical tests of basis truncation, we observed that using only ${x}_{\mu }^{{{{{{{{\rm{DC}}}}}}}}}$ as indicator is not enough for obtaining an accurate spectrum. Another indicator ${x}_{\mu }^{{{{{{{{\rm{IP}}}}}}}}}$ is introduced which measures the Importance of Propagation stability (IP) of the *μ*th AO basis function (see Eq. (14)). *C*_*μj*_(*t*) denotes an element in transformation matrix (from AO to MO basis), *S*_*t*_ computes the standard deviation along the time for each *μ* and *j*, and $\mathop{\sum }\nolimits_{j}^{{N}_{{{{{{{{\rm{MO}}}}}}}}}}$ sums over all standard deviations in MOs originating from the *μ*th AO basis function. The numerator of Eq. (14) indicates the variation (along RTP) of the contribution from each AO basis function to all MOs in transformation matrix ***C***(*t*). As for ${x}_{\mu }^{{{{{{{{\rm{DC}}}}}}}}}$, the dimensionless quantity ${x}_{\mu }^{{{{{{{{\rm{IP}}}}}}}}}$ is also constructed by dividing the numerator with its mean value of all AO basis functions. Small ${x}_{\mu }^{{{{{{{{\rm{IP}}}}}}}}}$ value means that the contributions to MOs from the *μ*th basis function do not change much compared to the contribution of all AO basis functions, and removing this basis function should not affect the propagation of the density matrix (remaining part) significantly. Note that both *O*_*μ*_(*t*) and *C*_*μj*_(*t*) are usually complex numbers for RTP, and the standard deviation of a set of complex numbers is calculated as in Supplementary Eq. (1).

14 $${x}_{\mu }^{{{{{{{{\rm{IP}}}}}}}}}=\frac{\mathop{\sum }\nolimits_{j}^{{N}_{{{{{{{{\rm{MO}}}}}}}}}}{S}_{t}[{C}_{\mu j}(t)]}{\frac{1}{{N}_{{{{{{{{\rm{AO}}}}}}}}}}\mathop{\sum }\nolimits_{\mu }^{{N}_{{{{{{{{\rm{AO}}}}}}}}}}\mathop{\sum }\nolimits_{j}^{{N}_{{{{{{{{\rm{MO}}}}}}}}}}{S}_{t}[{C}_{\mu j}(t)]}$$

In practice, an empirical parameter *x*^thr^ is chosen as threshold for both ${x}_{\mu }^{{{{{{{{\rm{DC}}}}}}}}}$ and ${x}_{\mu }^{{{{{{{{\rm{IP}}}}}}}}}$, where the AO basis functions with both indicators smaller than *x*^thr^ can be removed. The remaining basis set ${\{{\chi }_{\mu }\}}_{{{{{{{{\rm{trunc}}}}}}}}}$ (truncated AO basis set) is then defined as in Eq. (15), given the original AO basis set {*χ*_*μ*_} (of which the cardinality $|{\{{\chi }_{\mu }\}}|$ is *N*_AO_). Sometimes ${\{{\chi }_{\mu }\}}_{{{{{{{{\rm{trunc}}}}}}}}}$ includes only part of the given shell, e.g., for a *p*-shell, only ${\chi }^{{p}_{x}}$ and ${\chi }^{{p}_{y}}$ are in ${\{{\chi }_{\mu }\}}_{{{{{{{{\rm{trunc}}}}}}}}}$ and ${\chi }^{{p}_{z}}$ is truncated. Such symmetry breaking is mainly due to the utilization of polarized field (*δ*-pulse) in the RT-TDDFT calculations, and the rotational invariance requires a shell level truncation. Considering the truncated basis set used in any computational chemistry package, we also recommend shell level truncation for the general application of the basis set file. In most cases, the majority rule can be applied for a truncation at the shell level, namely, the shells containing more than half of their original basis functions remain in ${\{{\chi }_{\mu }\}}_{{{{{{{{\rm{trunc}}}}}}}}}$ while others are fully discarded. This has to be checked with the ${x}_{\mu }^{{{{{{{{\rm{DC}}}}}}}}}$ indicators of the basis functions in the same shell to ensure that there are no strong contribution to electric dipole transitions arising from some basis functions. In this study, the basis sets of (*S*)-methyloxirane, (-)-*α*-pinene, ZnPc, and Ag_20_ systems are truncated at the shell level.

**Fig. 1:**
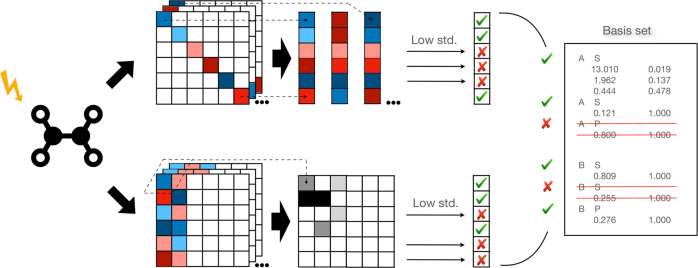
Schematic diagram of the basis set truncation process. First, a real-time propagation run of 1% (e.g., 100 steps) of the total simulation time is performed. Then the information of AO density matrix ***P***^AO^(*t*) and MO coefficient ***C***(*t*) at every step, and overlap matrix ***S***, is collected. Basis functions to be truncated are selected based on the low standard deviation (std. in the figure) of *O*_*μ*_(*t*) and *C*_*μj*_(*t*). Eventually, one can directly modify the basis set file for a complete RT-TDHF/TDDFT calculation or a LR-TDHF/TDDFT calculation.

The schematic view of the truncation process is shown in Fig. 1.

15 $${\{{\chi }_{\mu }\}}_{{{{{{{{\rm{trunc}}}}}}}}}=\left\{{\chi }_{\mu }\,|\,{x}_{\mu }^{{{{{{{{\rm{DC}}}}}}}}} \, > \, {x}^{{{{{{{{\rm{thr}}}}}}}}}\vee {x}_{\mu }^{{{{{{{{\rm{IP}}}}}}}}} \, > \,{x}^{{{{{{{{\rm{thr}}}}}}}}},\,\forall i\in \{1,...,{N}_{{{{{{{{\rm{AO}}}}}}}}}\}\right\}$$

Using ${\{{\chi }_{\mu }\}}_{{{{{{{{\rm{trunc}}}}}}}}}$, namely, reducing number of basis functions from *N*_AO_ to ${N}_{{{{{{{{\rm{trunc}}}}}}}}}=|{\{{\chi }_{\mu }\}}_{{{{{{{{\rm{trunc}}}}}}}}}|$, can ideally decrease the total computational time to ${({N}_{{{{{{{{\rm{trunc}}}}}}}}}/{N}_{{{{{{{{\rm{AO}}}}}}}}})}^{4}$ for a RT-TDHF calculation or a RT-TDDFT calculation with hybrid exchange-correlation functional. Also, this truncated basis set can be transferred to LR-TDHF/TDDFT calculations.

The procedure for carrying out RT-TDHF/TDDFT calculations with truncated AO basis set for the examples studied in this work is described as follows:

- Run 100 (400) steps (1% of the total simulation time) of RT-TDHF/RT-TDDFT simulation with the timestep 0.2 (0.05) atomic units using a preliminarily chosen basis set ${\{{\chi }_{\mu }\}}$, and collect the information regarding ***S***, ***P***^AO^(*t*), ***C***(*t*) of every step.
- Calculate ${x}_{\mu }^{{{{{{{{\rm{DC}}}}}}}}}$ and ${x}_{\mu }^{{{{{{{{\rm{IP}}}}}}}}}$ via Eqs. (13) and (14), respectively, and select truncated AO basis set ${\{{\chi }_{\mu }\}}_{{{{{{{{\rm{trunc}}}}}}}}}$ based on the criteria in Eq. (15).
- Run 10’000 (40’000) steps of RT-TDHF/TDDFT (full) simulation with the same timestep using ${\{{\chi }_{\mu }\}}_{{{{{{{{\rm{trunc}}}}}}}}}$. Note that a ground state SCF calculation should be carried out with the truncated basis set before the RT-TDHF/RT-TDDFT simulation in order to apply the perturbation to a converged ground state.

It is worth noting that the truncation procedure by construction eliminates the transitions that are not (or very weakly) electric dipole allowed, and thus this approach focuses more on the overall spectrum rather than the types of transitions.

### Complete basis set limit

In addition to the analyses of truncated AO basis functions, we introduce an algorithm to construct basis sets towards the CBS limit for RT(LR)-TDHF/TDDFT calculations.

The idea of CBS limit employed here is to add diffuse functions (see examples (*S*)-methyloxirane and (-)-*α*-pinene for the reason) to all types of AO basis functions (s, p, d, f, g, ...) representing different orbital angular momenta *l*. These functions are added in an even-tempered manner^26,27^ by a geometric progression of the orbital exponents in the original basis set: ${\alpha }_{l,k}={\alpha }_{l}{\beta }_{l}^{k},\,\forall k\in {\mathbb{N}}$. *α*_*l*,*k*_ is an exponent of the *l*-shell with *k*th power, and *α*_*l*_ and ${\beta }_{l}^{k}$ are two parameters to be optimized for the basis set. Since most basis sets available (Pople^28^, Dunning^29^, Jensen^30^, Ahlrichs^31,32^, etc.) provide more than one exponent for each type of shell, we can directly extrapolate from these values to get the additional exponent ${\alpha }_{l,k+1}={\alpha }_{l,k}^{2}/{\alpha }_{l,k-1}$. One can increase the *k* value until significant linear dependencies are found in the basis set (sometimes also referred to as basis set overcompleteness^33^).

This CBS scheme usually requires a quite large basis set for the calculation, and it is usually unclear which basis function(s) should be removed once overcompleteness is reached. Therefore, we combine it with AO truncation and propose an “Add-While-Truncate” algorithm (see Algo. 1) to construct the CBS specifically designed for RT(LR)-TDHF/TDDFT calculations. Firstly, a preliminarily chosen basis set ${\{{\chi }_{\mu }\}}$ is used for a short period of RT-TDHF/TDDFT calculation and ${\{{\chi }_{\mu }\}}_{{{{{{{{\rm{trunc}}}}}}}}}$ is selected. An additional basis set containing diffuse functions (${\{{\chi }_{\mu }\}}_{{{{{{{{\rm{diffuse}}}}}}}}}$) is constructed in an even-tempered manner. ${\{{\chi }_{\mu }\}}_{{{{{{{{\rm{diffuse}}}}}}}}}$ may contain some basis functions truncated in previous steps (combined as ${\{{\chi }_{\mu }\}}_{{{{{{{{\rm{deleted}}}}}}}}}$), which should be removed. Then ${\{{\chi }_{\mu }\}}_{{{{{{{{\rm{diffuse}}}}}}}}}$ is combined with ${\{{\chi }_{\mu }\}}_{{{{{{{{\rm{trunc}}}}}}}}}$ to form a new basis set ${\{{\chi }_{\mu }\}}$. In order to check the overcompleteness of the newly created AO basis set, we calculate the overlap matrix ${{{{{{{{\boldsymbol{S}}}}}}}}}_{\{{\chi }_{\mu }\}}$ and solve for its eigenvalues *λ*. If the minimal absolute eigenvalue ∣*λ*∣_*min*_ is smaller than a user-defined small value *ϵ* or ${\{{\chi }_{\mu }\}}$ remains the same as in the last cycle (namely, basis functions are neither truncated nor added), ${\{{\chi }_{\mu }\}}$ is regarded as the CBS under such *ϵ*-condition (${\{{\chi }_{\mu }\}}_{{{{{{{{\rm{CBS-}}}}}}}}\epsilon }$), otherwise the new ${\{{\chi }_{\mu }\}}$ is used to repeat the previous steps until the final condition is fulfilled. In practice, one can also manually remove some newly added diffuse functions within ${\{{\chi }_{\mu }\}}$ in the iteration to satisfy the given *ϵ*-condition. In this case, in order to minimize the total number of basis functions, we first remove the diffuse functions corresponding to higher orbital angular momentum, which is the same idea as the one applied in calendar basis sets^34^. For the sake of simplicity, we use the term “basis functions” for “AO basis functions" in the remaining part of this manuscript.

#### Algorithm 1

**Add-While-Truncate CBS Algorithm**

1: **repeat**

2: $${\{{\chi }_{\mu }\}}_{{{{{{{{\rm{old}}}}}}}}}\leftarrow \{{\chi }_{\mu }\}$$

3: Run a RT-TDHF/TDDFT simulation with ${\{{\chi }_{\mu }\}}$ for 100 (400) steps.

4: Construct ${\{{\chi }_{\mu }\}}_{{{{{{{{\rm{trunc}}}}}}}}}$ by Eqs. (13)–(15)

5: Construct additional even-tempered basis set ${\{{\chi }_{\mu }\}}_{{{{{{{{\rm{diffuse}}}}}}}}}$ for ${\{{\chi }_{\mu }\}}_{{{{{{{{\rm{trunc}}}}}}}}}$

6: $${\{{\chi }_{\mu }\}}_{{{{{{{{\rm{deleted}}}}}}}}}\leftarrow {\{{\chi }_{\mu }\}}_{{{{{{{{\rm{deleted}}}}}}}}}\cup (\{{\chi }_{\mu }\}\setminus {\{{\chi }_{\mu }\}}_{{{{{{{{\rm{trunc}}}}}}}}})$$

7: $${\{{\chi }_{\mu }\}}_{{{{{{{{\rm{diffuse}}}}}}}}}\leftarrow {\{{\chi }_{\mu }\}}_{{{{{{{{\rm{diffuse}}}}}}}}}\setminus {\{{\chi }_{\mu }\}}_{{{{{{{{\rm{deleted}}}}}}}}}$$

8: $$\{{\chi }_{\mu }\}\leftarrow {\{{\chi }_{\mu }\}}_{{{{{{{{\rm{trunc}}}}}}}}}\cup {\{{\chi }_{\mu }\}}_{{{{{{{{\rm{diffuse}}}}}}}}}$$

9: Solve for eigenvalues *λ* of overlap matrix ${{{{{{{{\boldsymbol{S}}}}}}}}}_{\{{\chi }_{\mu }\}}$

10: **until** ∣*λ*∣_*min*_ < *ϵ ***or **$\{{\chi }_{\mu }\}={\{{\chi }_{\mu }\}}_{{{{{{{{\rm{old}}}}}}}}}$

11: $${\{{\chi }_{\mu }\}}_{{{{{{{{\rm{CBS-}}}}}}}}\epsilon }\leftarrow {\{{\chi }_{\mu }\}}_{{{{{{{{\rm{trunc}}}}}}}}}$$

### Example: H_2_ dimer

**Fig. 2:**
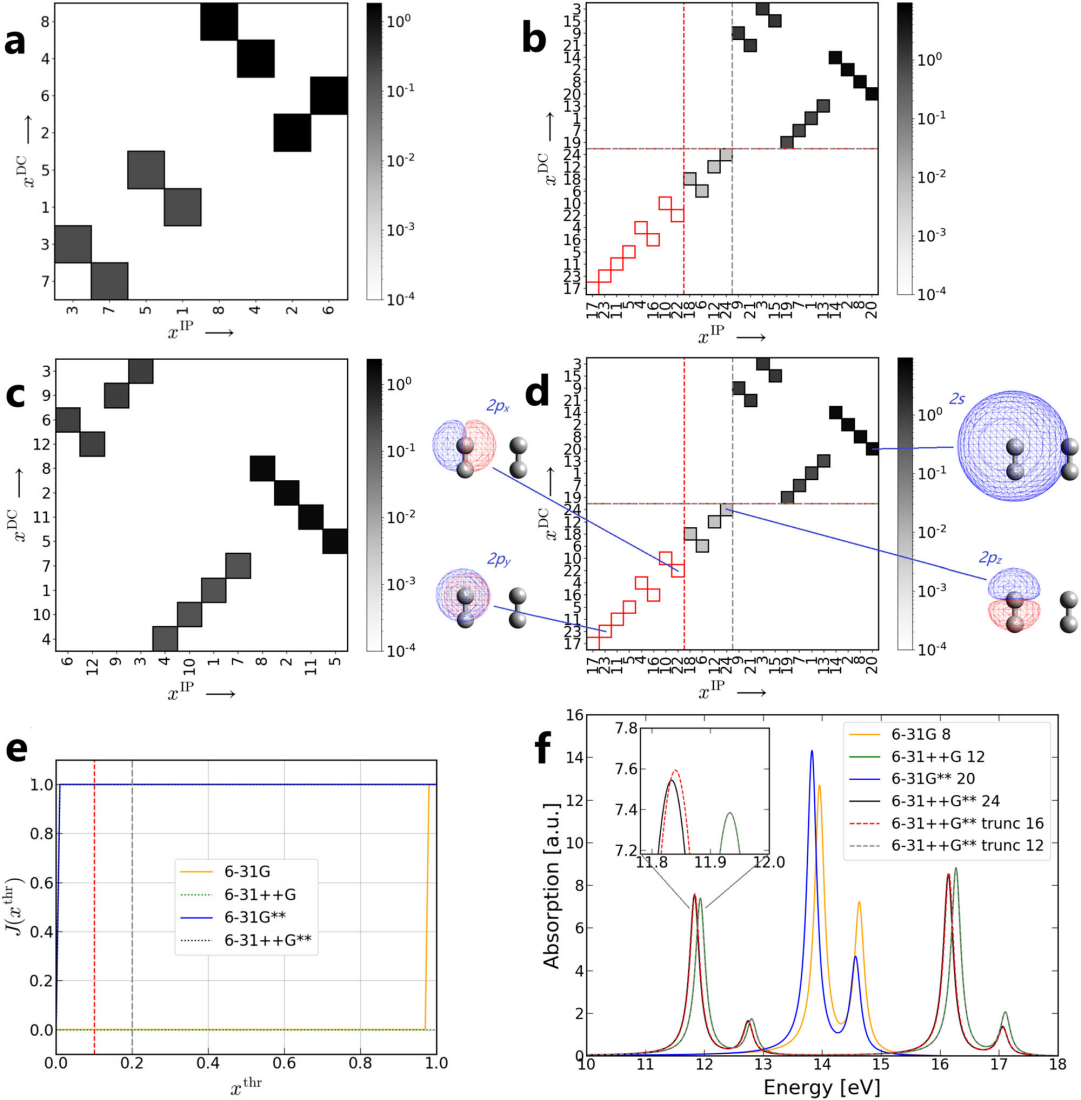
RT-TDHF calculations of the H_2_ dimer. *x*^DC^-*x*^IP^ map obtained with **a** 6-31G, **b** 6-31G**, **c** 6-31++G, and **d** 6-31++G** basis set. Each square in the map represents a basis function with its numbering on *x* and *y* axes. The color of the squares is based on the value calculated as *x*^DC^ ⋅ *x*^IP^, so the deeper the color the more important the basis function is in the TDHF calculation. **d** includes the visualization of related basis functions. Axis labels (basis functions) are sorted in ascending order according to their *x*^DC^ or *x*^IP^ values. The red dashed line represents *x*^thr^ = 0.1 and the gray dashed line represents *x*^thr^ = 0.2. **e** Jaccard indices for 6-31G, 6-31G**, 6-31++G, and 6-31++G** basis sets. The red dashed line represents *x*^thr^ = 0.1 and the gray dashed line represents *x*^thr^ = 0.2. **f** Electronic absorption spectra using 6-31G, 6-31G**, 6-31++G, 6-31++G**, and truncated 6-31++G** basis sets with *x*^thr^ = 0.1 (6-31++G** trunc 16) and *x*^thr^ = 0.2 (6-31++G** trunc 12). The number after the basis set label indicates the number of basis functions used for the RTP. *a.u.* arbitrary units.

The H_2_ dimer is used as the first test system, with the *δ*-pulse applied along *z* direction (see the geometry in Fig. 2d, *z* axis is parallel to the H–H bond, and *y* axis is perpendicular to the plane formed by the four atoms). Four different basis sets, 6-31G, 6-31G**, 6-31++G, and 6-31++G**^28,35,36^, are utilized for RT-TDHF calculations.

For each H atom, 6-31G contains two s-type basis functions (noted as 2s for convenience), 6-31G** contains 2s1p (1*p* as extra polarization function, we use *italic* form to represent specific basis function(s), e.g., 1*p* means the first p-type basis function), 6-31++G contains 3s (3*s* as an extra diffuse function), and 6-31++G** contains 3s1p. Note that the abbreviations we use here refer to basis functions but not specific electron shells. The same convention of basis/orbital notations is utilized for all examples in this study. E.g. for a truncation from 5s4p to 5s3p ( −3*p*), −3*p* means the 3^rd^ p-type basis function is removed but the 1^st^, 2^nd^, and 4^th^ p-type basis functions and all s-type basis functions remain.

After 100 steps of RT-TDHF calculations, *x*^DC^ and *x*^IP^ of each basis function are computed and can be visualized in Fig. 2a–d as *x*^DC^-*x*^IP^ map. Basis functions are represented by colored squares with their value *x*^DC^ ⋅ *x*^IP^. The electric dipole contribution and importance of propagation stability of basis functions are sorted in two axes. The red dashed line and the gray dashed line represent *x*^thr^ = 0.1 and *x*^thr^ = 0.2, respectively. *x*^thr^ splits the *x*^DC^-*x*^IP^ map into four quadrants: important for both electric dipole contribution and propagation stability (top right), important for only electric dipole contribution (top left), important for only propagation stability (bottom right), and important for neither one (bottom left). The basis functions locate inside the left below region (red borders for *x*^thr^ = 0.1) are the ones recommended to be deleted from the basis set.

One can find that no basis function is to be deleted in the case of 6-31G (Fig. 2a) and 6-31++G (Fig. 2c) basis sets, and there are 8 basis functions to be deleted in the case of 6-31G** (Fig. 2b) and 6-31++G** (Fig. 2d) basis sets with *x*^thr^ = 0.1. These 8 basis functions are the same for 6-31G** and 6-31++G**: 2*p*_*x*_ and 2*p*_*y*_ of each H atom, which belong to the polarization functions. If *x*^thr^ is set to 0.2 (gray dashed lines in Fig. 2b, d), extra four basis functions are to be deleted in both cases: 2*p*_*z*_ of each H atom. Therefore, a setting of *x*^thr^ = 0.2 essentially truncates 6-31G** to 6-31G and 6-31++G** to 6-31++G for the H_2_ dimer. Note that the pulse causes differences between atoms with different nuclear Cartesian coordinates, leading to different *x*^DC^-*x*^IP^ values of the same basis functions in different H atoms. In Supplementary Fig. 1, we further provide a more intuitive view of *O*_*μ*_ from each basis function in 6-31++G and 6-31++G** basis sets. One can easily distinguish small contribution components from the large contribution ones.

Let us take a closer look at the *x*^DC^-*x*^IP^ map in the case of 6-31++G** basis set (see basis functions shown in Fig. 2d, we focus only on one H atom here). Actually, we can explain qualitatively that 2*p*_*x*_ and 2*p*_*y*_ are the least important basis functions for the simulation of the electronic absorption spectrum. The electric dipole transitions from *σ*_1*s*−1*s*_ to ${\pi }_{2{p}_{x}-2{p}_{x}}$, ${\pi }_{2{p}_{x}-2{p}_{x}}^{*}$, ${\pi }_{2{p}_{y}-2{p}_{y}}$, and ${\pi }_{2{p}_{y}-2{p}_{y}}^{*}$ are almost (considering the effect from the other H_2_ molecule close-by) forbidden due to symmetry reasons. The *x*^DC^-*x*^IP^ map shows that 2*p*_*x*_ is slightly more important than 2*p*_*y*_, which may be explained by a stronger interaction on the *x* direction between H_2_ molecules. The electric dipole transition ${\pi }_{2{p}_{z}-2{p}_{z}}^{*}\leftarrow {\sigma }_{1s-1s}$ is allowed, and thus the 2*p*_*z*_ basis function is considered to be more important than 2*p*_*x*_ and 2*p*_*y*_ for the electronic absorption spectrum. The electric dipole transition ${\sigma }_{2s-2s}^{*}\leftarrow {\sigma }_{1s-1s}$ is also allowed and ${\sigma }_{2s-2s}^{*}$ has an lower energy than ${\pi }_{2{p}_{z}-2{p}_{z}}^{*}$, which leads to a higher occupation probability. Therefore, 2*s* in 6-31++G** basis set is one of the “dominant” basis functions in the RTP for H_2_ with the computational settings used.

In more complex systems, such an energetic analysis in terms of “static” wavefunctions (e.g., wavefunctions after SCF) is not enough to give a reasonable truncated basis set since MO coefficients ***C***(*t*) are time-dependent, which explains our choice of using first 100 (400) steps for the analysis.

Besides, a Jaccard index^37^ ( *J*(*x*^thr^)) is applied to analyze the similarity of deleted basis functions suggested by our two criteria *x*^DC^ and *x*^IP^ (see Eq. (16)). A high Jaccard index indicates more basis functions in common between two sets and vice versa. This information provides an intuitive view of the truncation along *x*^thr^ for a given basis set.

16 $$J({x}^{{{{{{{{\rm{thr}}}}}}}}})=\frac{|\{{\chi }_{\mu }\,|\,{x}_{\mu }^{{{{{{{{\rm{DC}}}}}}}}} \, < \, {x}^{{{{{{{{\rm{thr}}}}}}}}}\}\cap \{{\chi }_{\mu }\,|\,{x}_{\mu }^{{{{{{{{\rm{IP}}}}}}}}} \, < \, {x}^{{{{{{{{\rm{thr}}}}}}}}}\}|}{|\{{\chi }_{\mu }\,|\,{x}_{\mu }^{{{{{{{{\rm{DC}}}}}}}}} \, < \, {x}^{{{{{{{{\rm{thr}}}}}}}}}\}\cup \{{\chi }_{\mu }\,|\,{x}_{\mu }^{{{{{{{{\rm{IP}}}}}}}}} \, < \, {x}^{{{{{{{{\rm{thr}}}}}}}}}\}|}$$

For H_2_ dimer system, it is found that *J*(*x*^thr^) of 6-31G** and 6-31++G** remains at a value of 1.0 from *x*^thr^ = 0.01 to *x*^thr^ = 1.0 (see Fig. 2e). This shows that RTP has clear “preference” for some basis functions within the given basis sets. In the case of 6-31G and 6-31++G basis sets, on the other hand, *J*(*x*^thr^) remains at a value of 0.0 up to *x*^thr^ = 0.9, which means that no redundant basis functions are found for such basis sets.

The spectra using different basis sets and truncated 6-31++G** basis sets are in Fig. 2f. The spectra using 6-31G and 6-31G** basis sets look very similar, which matches the truncation suggestion given in Fig. 2b. The same situation is also found in the spectra using 6-31++G and 6-31++G** basis sets. Truncated 6-31++G** basis set with *x*^thr^ = 0.2 (noted as 6-31++G** trunc 12, 12 basis functions left) leads to the same basis set as 6-31++G, with an error of ~ 0.1 eV of corresponding excitation energies. With a tighter threshold *x*^thr^ = 0.1, truncated 6-31++G** basis set (noted as 6-31++G** trunc 16, 16 basis functions left) achieves more accurate spectra compared to 6-31++G basis set, with an error at the level of ~0.01 eV. These results are in accordance with the *x*^DC^-*x*^IP^ map introduced before. For the sake of completeness, the cases of *δ*-pulse from *x* or *y* direction are included in Supplementary Fig. 2.

### Example: H_2_O dimer

**Fig. 3:**
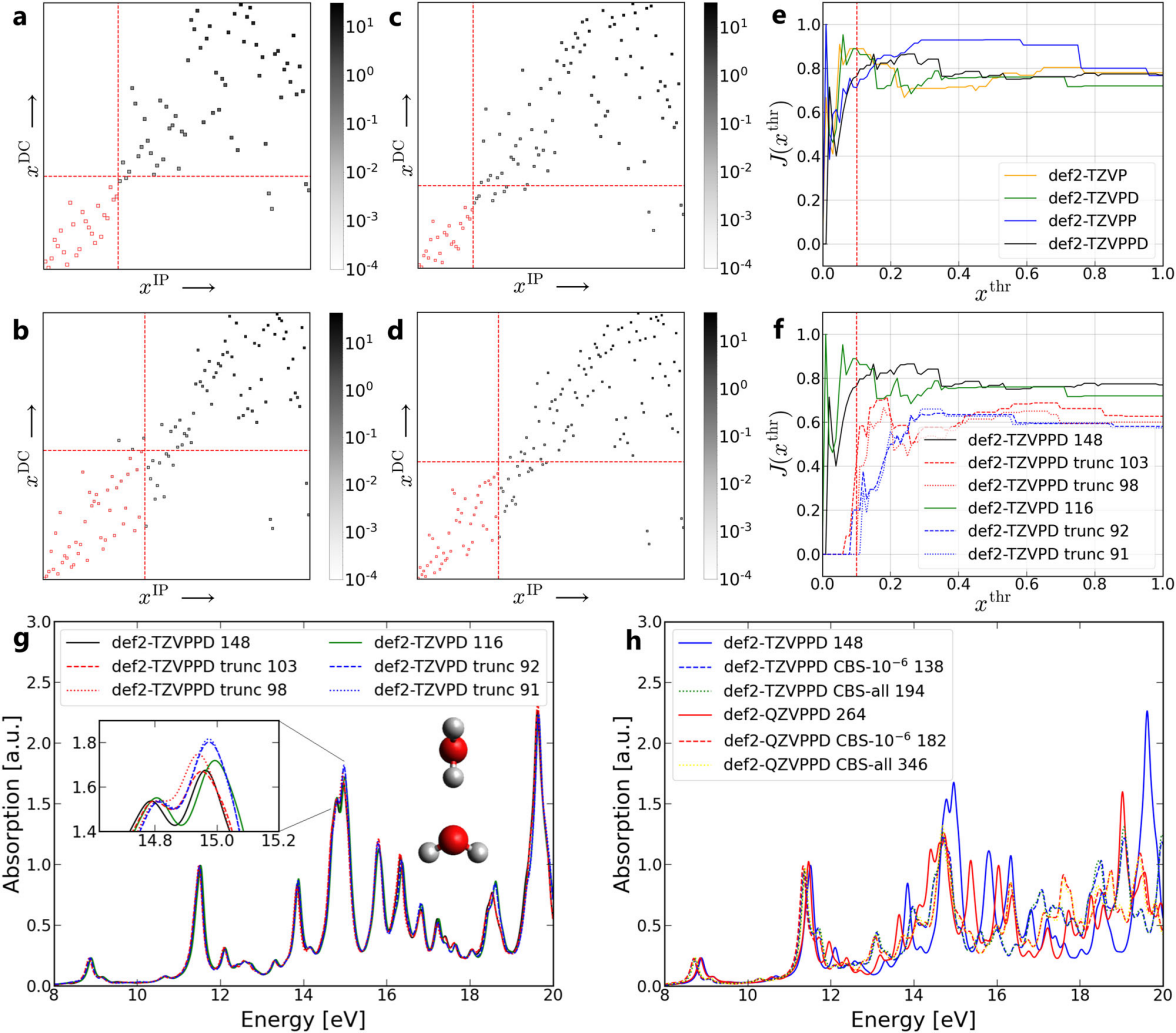
RT-TDHF calculations of the H_2_O dimer. *x*^DC^-*x*^IP^ map obtained with **a** def2-TZVP, **b** def2-TZVPP, **c** def2-TZVPD, and **d** def2-TZVPPD basis set. Each square in the map represents a basis function with its numbering on *x* and *y* axes. The color of the squares is based on the value calculated as *x*^DC^ ⋅ *x*^IP^, so the deeper the color the more important the basis function is in the TDHF calculation. The red dashed line represents *x*^thr^ = 0.1. **e** Jaccard indices for def2-TZVP, def2-TZVPP, def2-TZVPD, and def2-TZVPPD basis sets, and **f** def2-TZVPD, def2-TZVPPD, and corresponding truncated/recursively truncated basis sets. The vertical red dashed line represents *x*^thr^ = 0.1. **g** Electronic absorption spectra using original, one-time truncation, and recursive truncation of def2-TZVPD, def2-TZVPPD basis sets. The truncation threshold is *x*^thr^ = 0.1. **h** Electronic absorption spectra using original, CBS-10^−6^, and CBS-all (combined basis set of the original one and CBS-10^−6^) of def2-TZVPPD and def2-QZVPPD basis sets. The truncation threshold used in the CBS scheme is *x*^thr^ = 0.1. The number after the basis set label indicates the number of basis functions used for the RTP. *a.u.* arbitrary units.

Four different basis sets, def2-TZVP, def2-TZVPP, def2-TZVPD, and def2-TZVPPD^32,38^ are utilized for RT-TDHF calculations of H_2_O dimer system (see Fig. 3g for the nuclear structure). Again, these four basis sets are chosen based on the addition of polarization functions and/or diffuse functions. The *x*^DC^-*x*^IP^ map of this system is shown in Fig. 3a–d after 100 steps of RT-TDHF calculations. Compared to H_2_ dimer, H_2_O dimer is a more complicated system and thus the *x*^DC^-*x*^IP^ map is more involved. Nevertheless, it is clear that the distribution of basis functions in all plots shows a “dumbbell” shape (from left below to right top), namely, more dispersed in low and high *x*^DC^/*x*^IP^ region compared to the middle range. This provides a rough idea of the range of truncation, and the cross point of *x*^thr^ = 0.1 generally locates at the neck of the “dumbbell”. The Jaccard indices for the four basis sets are shown in Fig. 3e. In Supplementary Figure 3, some visualizations of orbitals are shown in the *x*^DC^-*x*^IP^ map of the def2-TZVP basis set.

A natural question of basis set truncation is whether one needs to do it recursively until the basis set does not change anymore (which we refer to as “recursive truncation”). Therefore, we test the recursive truncation of def2-TZVPD and def2-TZVPPD basis sets, and provide the number of basis functions together with Jaccard indices (see Fig. 3f). One-time truncation decreases the number of basis functions from 148 to 103 and from 116 to 92 for the two original basis sets, while recursive truncation only decreases the number further from 103 to 98 and from 92 to 91, respectively. It is worth mentioning that the two basis sets are very similar as one can see from the number of basis functions after the recursive truncation. The Jaccard indices give visual evidence that “def2-TZVPD trunc 91” and “def2-TZVPPD trunc 98” do not change after another truncation process since *J*(*x*) = 0 for *x* ∈ [0, 0.1]. It also shows that one-time truncation is good enough to significantly decrease *J*(*x*^thr^) value (see two dashed lines). Considering the time and computational resources spent on recursive truncation process (usually needs several rounds of RT-TDHF/TDDFT calculation), we only focus on one-time truncation in the following.

The spectra of H_2_O dimer using def2-TZVP, def2-TZVPP, def2-TZVPD, and def2-TZVPPD basis sets give a similar conclusion as in H_2_ dimer case, namely, that extra diffuse functions have large impact on the absorption spectra while extra polarization functions have limited impact on the absorption spectra (see Supplementary Figure 4). In addition, we provide the spectra after a one-time truncation and recursive truncation processes (see Fig. 3g). All spectra in this figure are very close to each other up to an excitation energy of 20 eV, indicating def2-TZVPPD includes many redundant basis functions for RT-TDHF calculations in this case. For the usage of computational resources, in the case of ${{{{{{{\mathcal{O}}}}}}}}({N}^{4})$ scaling (HF Coulomb and exchange matrices calculated with 2-electron integrals, without real-space griding or density-fitting), a RT-TDHF run with “def2-TZVPD trunc 91” basis set only consumes (91/148)^4^ = 14 % of the time compared to the original def2-TZVPPD basis set. For this system, a special interest is the effect of hydrogen bonds on the truncation process. However, we do not find any dependence of deleted basis functions on the distance (up to 10 Å) between two water molecules, and the suggested truncated basis sets are very similar. This may indicate that the basis functions needed for the description of hydrogen bonds are also important for the electronic absorption spectrum of the water monomers themselves.

Moreover, the CBS scheme is tested for the H_2_O dimer system. Two basis sets, def2-TZVPPD and def2-QZVPPD are used as the starting point for the CBS scheme, with *ϵ* = 10^−6^ (CBS-10^−6^). We directly modify the basis set file every time when truncating or adding basis functions. The detailed steps of the CBS scheme for def2-TZVPPD basis set are shown in Table 1. The original def2-TZVPPD basis set of the H atom and the O atom is 3s3p1d and 6s4p3d1f, respectively, with 148 basis functions for the H_2_O dimer system. After the first RT-TDHF run, the first d-subshell (1*d*) of H and the first d-subshell and f-subshell of O (1*d*1*f*) are truncated (shown in the bracket). The diffuse functions are then added to the remaining subshells (shown in the bracket), resulting in 4s4p for H and 7s5p3d for O. This is followed by a second RT-TDHF run with truncating and adding basis function. With a basis set of 5s4p for H and 8s6p4d for O, we find ∣*λ*∣_*min*_ is smaller than the threshold we set (10^−6^), thus the newly added subshell with the highest angular moment is removed, say, 4*p* in H and 4*d* in O. Finally, we obtain a basis set of 5s3p for H and 8s6p3d for O, with 138 basis functions and ∣*λ*∣_*min*_ = 3.7 × 10^−6^. In Supplementary Table 1, we do it analogously for def2-QZVPPD basis set.

**Table 1:** The steps of the CBS scheme for def2-TZVPPD basis set in the H_2_O dimer system

Basis set	H	O	No. basis function
Original	3s3p1d	6s4p3d1f	148
Run 1			
Truncate	3s3p (−1*d*)	6s4p2d (−1*d*1*f*)	104
Add	4s4p (+4*s*4*p*)	7s5p3d (+7*s*5*p*3*d*)	138
Run 2			
Truncate	4s3p (−1*p*)	7s5p3d	126
Add	5s4p (+5*s*4*p*)	8s6p4d (+8*s*6*p*4*d*)	160
∣*λ*∣_*min*_ < 10^−6^			
Remove	5s3p (−4*p*)	8s6p3d (−4*d*)	138
Run 3			
Truncate	5s3p (none)	8s6p3d (none)	138
∣*λ*∣_*min*_ = 3.7 × 10^−6^			

The truncation threshold used in the CBS scheme is x^thr^ = 0.1. Basis set information of H and O atoms is noted as *non-italic* form, and the added/truncated/removed basis functions are in *italic* form in the brakets. “Run” refers to a short time RT-TDHF calculation with 100 steps. In the last column, the total number of basis functions of the system is listed.

RT-TDHF calculations are carried out with these two CBSs, and the resulting spectra are shown in Fig. 3g. Choosing either the def2-TZVPPD or the def2-QZVPPD basis set (blue and red solid lines) can result in differences of absorption peaks with an excitation energy larger than 12 eV, while their corresponding CBSs-10^−6^ (blue and red dashed lines) match until 15 eV. Also, CBSs-10^−6^ leads to a red shift of 0.2 eV compared to the two original basis sets, which usually indicates the behavior of a larger basis set according to the observations in this work, while in our case this is achieved by less basis functions. In addition, we utilize combined basis sets of original basis set and its CBS-10^−6^ (noted as CBS-all) for the same calculation, and we can see that each spectrum (dotted lines) also agrees with the corresponding CBS-10^−6^ one.

### Examples: (***S***)-methyloxirane and (-)-***α***-pinene

As other examples, (*S*)-methyloxirane and (-)-*α*-pinene molecules are tested with truncated basis sets. The def2-TZVPP^32,38^ basis set is adopted as a reference basis set and the B3LYP functional is selected as the exchange-correlation functional for these two systems. *x*^thr^ = 0.1 and *x*^thr^ = 0.2 are used as the truncation threshold. Supplementary Tables 2 and 3 give information about the original and truncated basis sets for (*S*)-methyloxirane and (-)-*α*-pinene, respectively. The corresponding *x*^DC^-*x*^IP^ maps are in Supplementary Figs. 5 and 6.

**Fig. 4:**
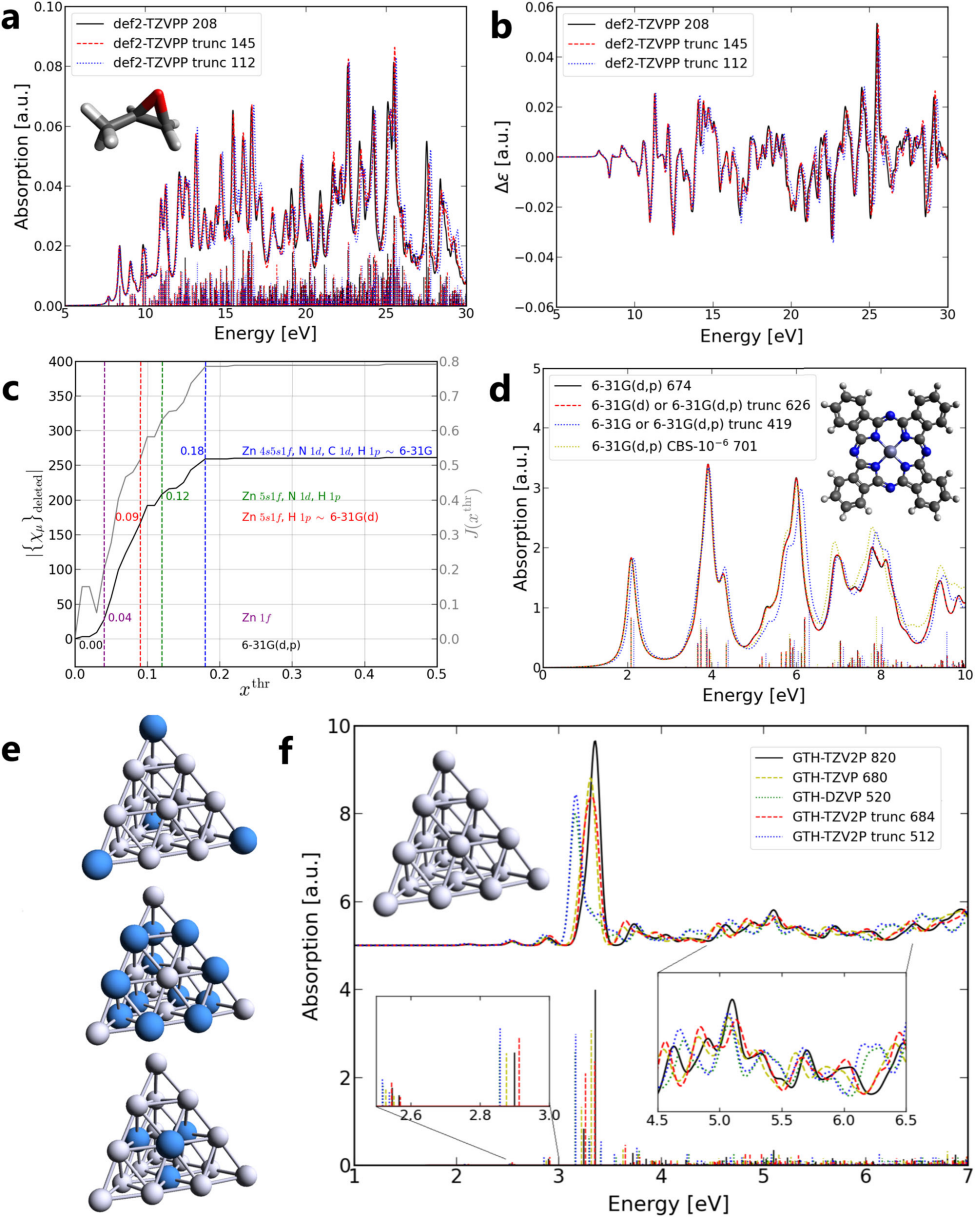
RT-TDDFT calculations of (***S***)-methyloxirane, ZnPc, and the Ag_20_ structures. (*S*)-methyloxirane **a** electronic absorption spectra and **b** electronic circular dichroism spectra using original and truncated basis sets of def2-TZVPP (stick spectra below correspond to LR-TDDFT ones). **c** Number of deleted basis functions ($|{\{{\chi }_{\mu }\}}_{{{{{{{{\rm{deleted}}}}}}}}}|$) and corresponding Jaccard indices of 6-31G(d,p) basis set for ZnPc system. Purple, red, green, and blue dashed lines represent different *x*^thr^ values, and the corresponding colored notations give the deleted basis functions in *italic* form. Note that the red dashed line with *x*^thr^ = 0.09 and blue dashed line with *x*^thr^ = 0.18 essentially reduce 6-31G(d,p) to 6-31G(d) and 6-31G basis set, respectively (except for the addition truncation on the Zn atom). **d** RT-TDDFT/LR-TDDFT electronic absorption spectra using 6-31G(d,p), 6-31G(d), 6-31G, and 6-31G(d,p) CBS-10^−6^ basis sets for ZnPc system (stick spectra below correspond to LR-TDDFT ones). **e** Ag_20_ geometry with colored vertex atoms (top), colored edge atoms (middle), and colored face atoms (bottom). **f** Ag_20_ LR-TDDFT electronic absorption spectra using GTH-TZV2P, GTH-TZVP, GTH-DZVP, and truncated GTH-TZV2P basis sets. Below are the stick spectra and above are the Gaussian broadened spectra with FWHM = 0.12 eV. The number after the basis set label indicates the number of basis functions. *a.u.* arbitrary units.

The resulting absorption spectra are shown in Fig. 4a and Supplementary Fig. 8a. The truncated basis sets, both *x*^thr^ = 0.1 and *x*^thr^ = 0.2, provide a good approximation to the absorption spectra compared to the original def2-TZVPP basis set, while using as few as half of the basis functions (in the case of *x*^thr^ = 0.2). Apart from the usage in RT-TDDFT, the truncation process is found to be robust in LR-TDDFT as well. In LR-TDDFT calculations, 500 and 2000 roots are solved for (*S*)-methyloxirane and (-)-*α*-pinene systems, respectively. From these results and the results in H_2_ dimer and H_2_O dimer systems, one may find that most basis functions truncated are polarization functions, e.g., p/d-subshell for H and d/f-subshell for C/O, while diffuse functions are usually not removed. This explains why the CBS scheme we propose only considers additional diffuse functions.

Furthermore, we use the same basis sets for ECD spectra calculations, considering that the two quantities *x*^DC^ and *x*^IP^ do not explicitly depend on the electric dipole operator. The ECD spectra of (*S*)-methyloxirane and (-)-*α*-pinene are shown in Fig. 4b and Supplementary Fig. 8b, respectively.

Table 2 gives the benchmark of basis sets used for (*S*)-methyloxirane and (-)-*α*-pinene. RT-TDDFT calculations of these two systems are carried out using CP2K. Because Coulomb and exchange and correlation (XC) terms are evaluated on grids, we do not observe a significant time-saving using the proposed truncated basis sets. Nevertheless, computational resources can be reduced as much as one order of magnitude in LR-TDDFT calculations (Gaussian09^39^) using truncated basis sets. The corresponding memory usage of (-)-*α*-pinene system is also shown in the table. The memory cost of Coulomb and exchange matrices scale as ${{{{{{{\mathcal{O}}}}}}}}({N}^{4})$ (2-electron integrals, without real-space griding or density-fitting), and one may easily encounter a memory bottleneck with large basis sets (e.g., using def2-TZVPP for (-)-*α*-pinene, maximal memory set to 200 *GB*), which, however, can be alleviated with truncated basis sets. In Supplementary Tables 4 and 5, we show the scaling information of (-)-*α*-pinene using HF/def2-TZVPP and its truncated basis set, and computational time to calculate Coulomb and exchange matrices, respectively. To assess the contribution from HF exchange term, we further show the difference between B3LYP/def2-TZVPP and BLYP/def2-TZVPP in the calculation of RT-TDDFT spectrum in Supplementary Fig. 7.

**Table 2:** Benchmark of the original and truncated basis sets of def2-TZVPP used in (*S*)-methyloxirane and (-)-*α*-pinene systems

System	Basis set	Time/Step (RTP)	Time [Memory] (LR)
	Def2-TZVPP	3.56 ± 0.22 *s*	767 *s*
(S)-methyloxirane	Trunc *x*^thr^ = 0.1	2.19 ± 0.19 *s*	214 *s*
	Trunc *x*^thr^ = 0.2	1.74 ± 0.16 *s*	111 *s*
	Def2-TZVPP	4.30 ± 0.54 *s*	38.4 *h* [198 *GB*]
(-)-α-pinene	Trunc *x*^thr^ = 0.1	2.27 ± 0.19 *s*	9.1 *h* [165 *GB*]
	Trunc *x*^thr^ = 0.2	1.44 ± 0.18 *s*	3.1 *h* [81 *GB*]

The truncation threshold used are xthr = 0.1 and x^thr^ = 0.2. The RT-TDDFT time is noted as time per step (40’000 steps in total) and LR-TDDFT time is noted as full computational time. The memory usage of LR-TDDFT calculations of (-)-α-pinene system is also presented in square brackets. LR-TDDFT calculations are processed with 8 CPU cores@3.3 GHz, and RT-TDDFT calculations are processed with 2 × 12 CPU cores@2.6 GHz and 32 × 12 CPU cores@2.6 GHz for (S)-methyloxirane and (-)-α-pinene, respectively. *s* second, *h* hour, *GB* gigabyte

### Example: ZnPc

ZnPc is a popular example for excited-state calculations^40–45^. This example is mainly utilized to demonstrate a step-by-step truncation from 6-31G(d,p) to 6-31G. Here we use the nuclear geometry of ZnPc from a previous study^45^ with the B3LYP functional and 6-31G(d,p)^28,35,36^ as the reference basis set. Figure 4c provides the information of deleted basis functions and Jaccard indices. The numbers close to the dashed lines are *x*^thr^ values, and the notations on the right side are details of deleted basis functions in *italic* form. Note that the number of deleted basis functions ($|{\{{\chi }_{\mu }\}}_{{{{{{{{\rm{deleted}}}}}}}}}|$) can be higher than the number calculated from the subshell notations on the right. This is because extra basis functions might also be deleted but not the corresponding full subshells, e.g., Zn 1*f  x*^thr^=0.04 corresponds to a $|{\{{\chi }_{\mu }\}}_{{{{{{{{\rm{deleted}}}}}}}}}|$ value larger than 7 because some other basis functions like H 1*p*_*z*_ (but not the full 1*p*) are deleted. In addition, this planar system, which we place in *x* − *y* plane in the simulation, shows some preferences for 1*d*_*xy*_ and $1{d}_{{x}^{2}-{y}^{2}}$ of C/N elements, and thus 1*d*_*yz*_, 1*d*_*xz*_, and $1{d}_{{z}^{2}}$ basis functions are the first to be deleted in the range of *x*^thr^ = 0.09 ~ 0.18, indicating that the truncation scheme can provide the information of preference on the orientation of basis functions (or AOs with different magnetic quantum number). It is worth mentioning that *x*^thr^ = 0.09 and *x*^thr^ = 0.18 truncation leads basically to the 6-31G(d) and 6-31G basis sets, except for the additional truncation on the Zn atom. Also, it is found that $|{\{{\chi }_{\mu }\}}_{{{{{{{{\rm{deleted}}}}}}}}}|$ and *J*(*x*^thr^) show a very similar trend. After *x*^thr^ = 0.18, both lines reach a plateau where seldom further basis functions can be removed, indicating 6-31G as a good truncated basis set. Actually, we can see this from *x*^DC^-*x*^IP^ map of the same system (see Supplementary Fig. 9) in which the truncated and remaining basis functions almost form two blocks with *x*^thr^ = 0.18 (dashed blue line).

The corresponding RT-TDDFT and LR-TDDFT (1000 roots) spectra are given in Fig. 4d. It is clear that 6-31G(d,p), 6-31G(d), and 6-31G basis sets all provide similar results, which match our truncation suggestions. This shows a practical usage of our truncation scheme on the selection of basis set. In addition, the CBS-10^−6^ with 6-31G(d,p) reference is constructed (see Supplementary Table 6 for the CBS process). Nevertheless, it does not change much in the RT-TDDFT/LR-TDDFT spectra compared to the original 6-31G(d,p) basis set.

### Example: Ag_20_

Ag_20_ is a metal cluster with tetrahedral structure (*T*_*d*_ symmetry), which has been investigated with TDDFT calculations^46–48^. Here we use the nuclear geometry of Ag_20_ from a previous study^48^ together with PBE0^49^ functional and GTH^50,51^ Gaussian-type pseudopotential basis sets^52^ GTH-DZVP, GTH-TZVP, and GTH-TZV2P. GTH-TZV2P is used as the reference basis set for the truncation process. The Ag atoms in Ag_20_ cluster are categorized into 3 groups: vertex (v), edge (e), and face (f) (see Fig. 4e). The atoms in the same group are equivalent in space and should have the same contribution to the electronic absorption spectrum. Table 3 shows the truncated basis functions versus increasing *x*^thr^ values. As one can see from the table, atoms in different groups generally have different suggested basis set truncations. More basis functions are truncated for atoms at vertex position, and less for atoms at face position. This is reasonable because vertex Ag atoms have a limited space angle “bonded” with other atoms, while face Ag atoms have half of their surrounding space occupied with 9 nearest neighbors, and complex surroundings often require more basis functions to describe the interactions. For comparison, basis set information of GTH-TZVP and GTH-DZVP is also listed. The truncation scheme provides quite different basis sets from GTH-TZVP and GTH-DZVP basis set, e.g., GTH-TZVP can be regarded as −2*f* truncated basis set of GTH-TZV2P, but 2*f* basis functions are the last choice of truncation from our scheme (up to *x*^thr^ = 0.6). This means that the corresponding standardly available smaller basis sets, e.g., GTH-DZVP or GTH-TZVP, do not always contain the most important basis functions (for TDDFT calculations) from the larger ones, e.g., GTH-TZV2P, which is different from what we have found for the ZnPc example system. We select *x*^thr^ = 0.3 and *x*^thr^ = 0.5 truncated basis sets for LR-TDDFT calculations, under the consideration that the numbers of basis functions are close to GTH-TZVP and GTH-DZVP basis sets, respectively.

**Table 3:** The truncation process of TZV2P basis set in Ag_20_ system

*x*^thr^	Truncated basis functions	No. truncated basis function	No. basis function [basis set]
0.0	–	0	820 [GTH-TZV2P]
	v: −3*p*		
0.0-0.1	e: −3*p*	60	760
	f: −3*p*		
	v: −3*p*1*f*		
0.1-0.3	e: −3*p*	88	732
	f: −3*p*		
	v: −3*p*3*d*1*f*2*f*		
0.3	e: −3*p*	136	684
	f: −3*p*		
–	v,e,f: −2*f*	140	680 [GTH-TZVP]
	v: −3*p*3*d*1*f*2*f*		
0.3-0.5	e: −3*p*1*f*	220	600
	f: −3*p*		
–	v,e,f: −3*p*3*d*2*f*	300	520 [GTH-DZVP]
	v: −3*p*3*d*1*f*2*f*		
0.5	e: −3*p*3*d*1*f*	308	512
	f: −3*p*1*f*		
	v: −3*p*3*d*1*f*2*f*		
0.5-0.6	e: −3*p*3*d*1*f*2*f*	392	428
	f: −3*p*1*f*		

Due to the T_d_ symmetry of the system, we can categorize Ag atoms into 3 groups: vertex (4 atoms), edge (12 atoms), and face (4 atoms), with the notation “v”, “e”, and “f”, respectively. Different truncation thresholds x^thr^ are employed and GTH-TZVP and GTH-DZVP basis set information are also listed for comparison. Truncated basis functions are in *italic* form.

The LR-TDDFT spectra (2000 roots) of the Ag_20_ cluster using 5 different basis sets are shown in Fig. 4f. In general, “GTH-TZV2P trunc 684” (*x*^thr^ = 0.3) gives better agreement with the reference GTH-TZV2P basis set than the one with the GTH-TZVP basis set. This can be seen as follows: 1. for the first several peaks (at ~ 2.6 eV, 2.9 eV, and 3.4 eV), red dashed peaks all locate closer to black peaks than yellow dashed peaks; 2. for the peaks up to 7 eV, the red dashed line follows closer to the black line than the yellow dashed line. However, the difference between the spectra calculated using “GTH-TZV2P trunc 512” (*x*^thr^ = 0.5) and GTH-DZVP basis sets is limited, which can be explained by their similar composition in terms of basis functions shown in Table 3. The Ag_20_ example demonstrates that the proposed truncation scheme has the ability of assigning different basis sets to the atoms, according to their “interaction” with the full system.

Additionally, we also provide some testing calculations to demonstrate: 1. 1% of the total propagation time is sufficient to show the contribution from each basis function, and the same truncation suggestion has been obtained using 1%, 10%, and 100% of the RTP steps (see Supplementary Fig. 10) 2. Indicator *x*^IP^ is necessary in the truncation scheme, and the truncation using only indicator *x*^DC^ can lead to a different spectrum (see Supplementary Fig. 11) 3. ERIs Schwarz screening does not affect the truncation scheme, and they can be used together for the acceleration in RT-TDHF/TDDFT calculations (see Supplementary Figs. 12-13 and Supplementary Tables 7-9).

### Discussion

**Fig. 5:**
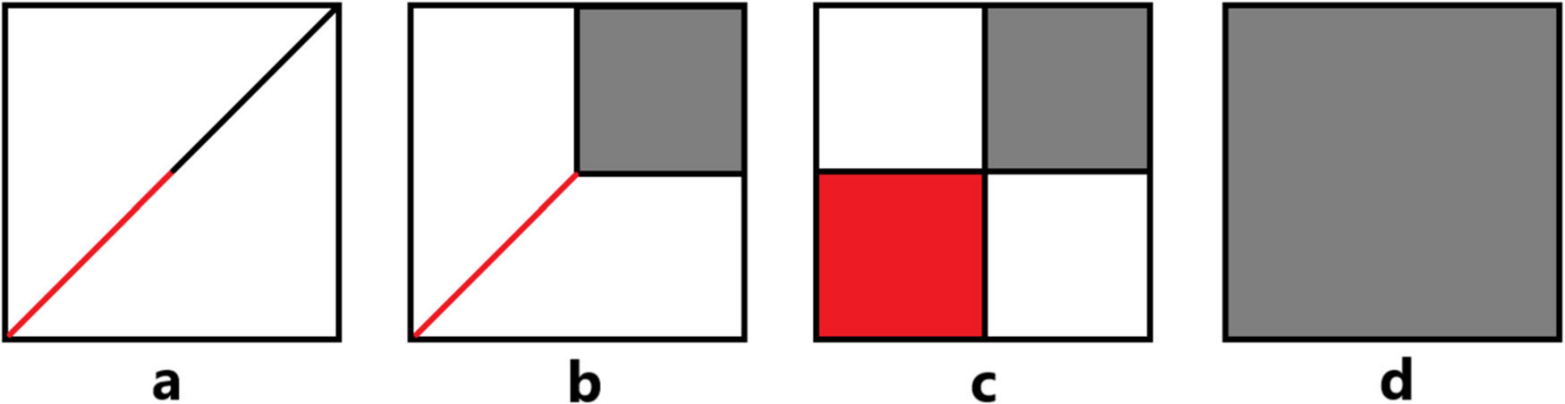
Diagrams of four different types of *x*^DC^-*x*^IP^ maps. Red/gray denote basis functions that can/cannot be removed. From left to right: **a** high correlation between *x*^DC^ and *x*^IP^, basis set can be truncated at any accuracy (e.g., H_2_ dimer 6-31G**, pulse *z* direction); **b** high correlation between *x*^DC^ and *x*^IP^ until certain *s* (where *s* is boundary of high and low correlation regions), basis set can be truncated at accuracy with *x*^thr^ ≤ *s* (e.g., H_2_ dimer 6-31++G**, pulse *z* direction); **c** two blocks with low correlation regions, basis set is recommended to be truncated at connected part between two blocks (e.g., all other test examples), which is also the most common case; **d** fully low correlation region, basis set can hardly be truncated (e.g., H_2_ dimer 6-31G/6-31++G, pulse *z* direction).

We have introduced an AO basis set truncation scheme for TDDFT calculations, based on the analysis of a short period of real time propagation of MO coefficients. Two quantities – density matrix contribution and importance of propagation stability – are constructed as indicators for the truncation process. The truncated basis sets are found to reproduce the electronic absorption spectra obtained with the original basis sets well. In some cases, truncated basis sets can serve as intermediate basis sets between two levels of available basis sets, or are found to be very close to lower level basis sets available, in which the truncation process works as a means to help in basis set selection. Two intuitive graphs, *x*^DC^-*x*^IP^ map and Jaccard index, are introduced for the analysis of basis functions. These graphs also provide a guide for the choice of the truncation threshold *x*^thr^ (e.g., see diagrams in Fig. 5).

As opposed to basis sets constructed mainly for the purpose of energy minimization and geometry optimization, the truncation scheme proposed provides a task-, system-, and chemical environment-specific basis set. It has reduced number of basis functions and accelerates the calculations involving the construction of Coulomb and/or exchange matrices iteratively in every propagation step, potentially with the scaling of ${{{{{{{\mathcal{O}}}}}}}}({N}^{4})$. ERIs usually benefit from evaluating all components from the same shell. However, they are only computed once before the propagation. Because the truncation process is carried out on the original AO basis set without any rotation or reconstruction of basis functions, the truncated basis sets can be easily employed in any quantum chemistry package using Gaussian-type (or Slater-type) basis, with a simple modification of the basis set file. Additionally, we have tested recursive truncation to show that the process is robust and will result in a “truncation consistent” basis set given a certain *x*^thr^. Though the truncation is based on the analysis of real-time propagation, the basis sets produced can also been used for LR-TDDFT calculations and provide equally good spectra. Nevertheless, the acceleration of LR-TDDFT calculations depends on the the systems and purposes of the research, e.g., limited number of excitations in LR-TDDFT for a small system may not be worth an additional RT-TDDFT calculation to determine a truncated basis set, while a highly conjugated or a large system with excitations of higher energies should benefit from the truncation scheme. How the truncation scheme and truncated basis set might be transferable to more accurate yet expensive methods like *GW*/Bethe-Salpeter equation^53–55^, time-dependent coupled cluster/configurational interaction^56–58^, or other type of any excited-state calculations might be explored in the future.

Furthermore, an “Add-While-Truncate” algorithm has been proposed to construct basis sets towards the complete basis set limit. The additional basis functions are added as diffuse functions in an even-tempered manner, and no extra polarization functions are added. The neglect of polarization functions is primarily based on truncation experiences we have got from this study (e.g., in H_2_O dimer, (*S*)-methyloxirane, and (-)-*α*-pinene systems). There are some discussions about the use of polarization and diffuse functions used for electric dipole moment, polarizability, and TDDFT calculations in previous works^59–63^. Nevertheless, as shown in test examples, the truncation process provides the possibility to select polarization and diffuse functions quantitatively. The proposed CBS scheme can construct basis sets to arbitrary accuracy, depending on a predefined parameter limited by the linear dependency between basis functions.

The truncation scheme might reveal some intrinsic knowledge for the better description of electronic excitations between ground state and excited states, and offer a thought for the design of basis sets in TDDFT calculations. Future work can be on both basis set constructions and migration to other excited-state calculations or properties. In this work, all original basis sets employed are ground state energy-optimized, however, there is another group of completeness-optimized basis sets^64–66^, with which one may also test the efficiency and validate the accuracy towards CBS-limit^65^. Auxiliary density matrix methods^67^ provide an alternative way to accelerate HF exchange calculation via auxiliary basis set, and have been found to yield highly accurate results in energies and response properties^63^. Considering the computational demanding HF exchange calculation employed in hybrid functionals, it is possible to further assess the truncation scheme for auxiliary basis sets. In addition, the idea of decomposing the electric dipole contributions into the contribution from individual basis functions can be migrated to other properties and produce different task specific basis sets. One may be interested in a truncated basis for dynamic calculations, which, however, might require further investigations on the consistency in the truncation for each nuclear configuration. Apart from basis set truncation, a direct basis set optimization algorithm (e.g., on exponents of Gaussian-type basis functions) is also possible given a proper loss function based on *x*^DC^ and *x*^IP^ parameters. While we have only tested the truncation process on neutral molecules in this study, charged systems could also be investigated. This would be an interesting topic since it may demonstrate the dependence of necessary basis functions on different charges for excited state calculations (e.g., effect of diffuse function on anions which is known for ground state cases).

In summary, our basis set truncation scheme provides a robust process for decreasing the number of basis functions and speeding up TDDFT calculations, while preserving the high accuracy of the spectra. The quantitative basis set analysis allows a profound understanding of the basis functions employed and opens up a broad area for potential research in excited state calculations.

## Methods

The systems H_2_ dimer, H_2_O dimer, (*S*)-methyloxirane, (-)-*α*-pinene, zinc phthalocyanine (ZnPc), and Ag_20_ have been investigated. Information about the applied computational methods, basis sets, and codes are listed in Table 4. For the H_2_ dimer and the H_2_O dimer, we utilize an in-house version of the PySCF^68,69^ RT-TDHF module^70^ to test truncation and CBS scheme. Calculations are carried out with 6-31G series^28,35,36^ w/o additional polarization/diffuse functions, and def2-TZVP series^32,38^ w/o additional polarization/diffuse functions. No Schwarz screening is used for the RT-TDHF calculations of H_2_ dimer and H_2_O dimer systems. For (*S*)-methyloxirane, (-)-*α*-pinene, and ZnPc, the CP2K (CP2K version 7.0 (Development Version), the CP2K developers group. CP2K is freely available from https://www.cp2k.org/.) package and the Gaussian09^39^ package is used for RT-TDDFT and LR-TDDFT (B3LYP^71^) calculations, respectively. For Ag_20_, Goedecker-Teter-Hutter (GTH) pseudopotential^50,51^ with the corresponding Gaussian-type pseudopotential basis sets^52^ GTH-DZVP, GTH-TZVP, and GTH-TZV2P, and PBE0^49^ hybrid functional are employed for time-dependent density functional perturbation theory (TDDFPT, up to the first order of the perturbation we use the term LR-TDDFT in this work) calculations using CP2K (CP2K version 7.0 (Development Version), the CP2K developers group. CP2K is freely available from https://www.cp2k.org/.) package. Schwarz screening threshold 10^−10^ (default in CP2K) is used in the RT-TDDFT calculations of (*S*)-methyloxirane, (-)-*α*-pinene, ZnPc, and Ag_20_ systems. All basis set files used in this work are from Basis Set Exchange^72^, visualization of molecular structures and orbitals uses Avogadro^73^ software, and graphs are generated with Matplotlib^74^.

**Table 4:** Systems and corresponding computational details

System	Methods	Basis sets	Codes
H_2_ dimer	RT-TDHF	6-31G,	PySCF^68, 69^
		6-31G**,	
		6-31++G,	
		6-31++G**	
H_2_O dimer	RT-TDHF	def2-TZVP,	PySCF^68, 69^
		def2-TZVPP,	
		def2-TZVPD,	
		def2-TZVPPD,	
		CBS-*ϵ*	
(*S*)-methyloxirane	RT-TDDFT,	def2-TZVPP	CP2K (CP2K version 7.0 (Development Version), the CP2K developers group. CP2K is freely available from https://www.cp2k.org/.),
(-)-*α*-pinene	LR-TDDFT		Gaussian09^39^
ZnPc	RT-TDDFT,	6-31G,	CP2K (CP2K version 7.0 (Development Version), the CP2K developers group. CP2K is freely available from https://www.cp2k.org/.),
	LR-TDDFT	6-31G(d),	Gaussian09^39^
		6-31G(d,p),	
		CBS-*ϵ*	
Ag_20_	LR-TDDFT	GTH-DZVP,	CP2K (CP2K version 7.0 (Development Version), the CP2K developers group. CP2K is freely available from https://www.cp2k.org/.)
		GTH-TZVP,	
		GTH-TZV2P	

The hybrid functional B3LYP is employed for the calculations of (S)-methyloxirane, (-)-α-pinene, and ZnPc, and PBE0 for the calculations of Ag20. Pseudopotentials are applied for Ag_20_, and all electron calculations for the other systems. Timestep: 0.05 atomic units for (S)-methyloxirane, (-)-α-pinene, ZnPc, 0.2 atomic units for the others.

A *δ*-pulse is chosen as the electric field perturbation to excite the molecules in RT-TDHF/TDDFT calculations. The application of the *δ*-pulse can be thought of as being applied instantly to the converged ground states MOs $|{\phi }^{0}\rangle$ between a time *t* = 0^−^ and *t* = 0^+^. It corresponds to an impulse^75^

17 $$\left|{\psi }^{\delta }(\overrightarrow{r},\, t={0}^{+})\right\rangle={e}^{-\frac{i}{\hslash }\overrightarrow{\kappa }\overrightarrow{r}}\left|{\psi }^{0}(\overrightarrow{r},\, t={0}^{-})\right\rangle,$$

where *ℏ* is reduced Planck constant. The vector $\overrightarrow{\kappa }$ indicates the direction and amplitude of the perturbation. The propagation is then started from the perturbed MOs $|{\psi }_{i}^{\delta }\rangle$.

## Supplementary information

Supporting Information

## Data Availability

The data generated in this study have been deposited at https://gitlab.uzh.ch/lubergroup/ao-truncation. Source data are provided in this paper.

## Source data

Source Data

## Code availability

Python codes for carrying out basis set truncation and RT-TDSCF calculations are available at https://gitlab.uzh.ch/lubergroup/ao-truncation.
