# Supplementary material for: Automatic purpose-driven basis set truncation for time-dependent Hartree–Fock and density-functional theory
Source: Nat Commun. 2023 Jan 6;14:106. doi: 10.1038/s41467-022-35694-4 (PMC9822955; doi:10.1038/s41467-022-35694-4)
Supplement: Supplementary file 1 — Supporting Information [file 41467_2022_35694_MOESM1_ESM.pdf]

Supplementary Information: Automatic purpose-drive basis set truncation for time-dependent Hartree–Fock and density-functional theory

R. Han[a], J. Mattiat[a], and S. Luber[a]*

[a]*Department of Chemistry A, University of Zurich, Winterthurerstrasse 190, 8057 Zurich, Switzerland*

E-mail: sandra.luber@chem.uzh.ch

Supplementary Note 1

Geometry information of related systems in Cartesian coordinates: H_2 dimer, H_2O dimer, (S)-methyloxirane, (-)-α-pinene, ZnPc, and Ag_{20}.

Supplementary Equation 1: the standard deviation of a set of complex numbers.

Supplementary Figure 1: electric dipole contribution (O_μ) of H_2 dimer from each basis function using two basis sets (6-31++G and 6-31++G**). Supplementary Figure 2: x^{DC}-x^{IP} map of H_2 dimer with 6-31++G** basis set and δ-pulse from x or y direction. Supplementary Figure 3: visualization of related basis functions in x^{DC}-x^{IP} map of H_2O dimer system. Supplementary Figure 4: H_2O dimer RT-TDHF spectra using def2-TZVP, def2-TZVPP, def2-TZVPD, and def2-TZVPPD basis sets. Supplementary Figures 5-6: x^{DC}-x^{IP} map of (S)-methyloxirane and (-)-α-pinene using def2-TZVPP basis set. Supplementary Figure 7: (-)-α-pinene RT-TDDFT spectra using B3LYP and BLYP functional. Supplementary Figure 8:

(-)-α-pinene electronic absorption spectra (RT-TDDFT/LR-TDDFT) and electronic circular dichroism spectra (RT-TDDFT) using original and truncated basis sets of def2-TZVPP. Supplementary Figure 9: x^{DC}-x^{IP} map of ZnPc using 6-31G(d,p) basis set. Supplementary Figure 10: x^{DC}-x^{IP} map of H_2O dimer using different RTP steps in the RT-TDHF calculation with def2-TZVP basis set. Supplementary Figure 11: H_2O dimer electronic absorption spectra and electric dipole moments in the first RTP 100 steps using original and truncated basis sets of def2-TZVP. Supplementary Figure 12: H_2O dimer RT-TDHF spectra using the original and truncated basis sets of def2- TZVPPD, and the same basis sets with Schwarz screening threshold 10^{-4}. Supplementary Figure 13: (S)-methyloxirane electronic absorption spectra using the original and truncated basis sets of def2-TZVPP, and def2-TZVPP basis set with Schwarz screening threshold 10^{-4}.

Supplementary Table 1: The steps of the CBS scheme for def2-QZVPPD basis set in H_2O dimer system. Supplementary Table 2: Information of the original and truncated basis sets of def2-TZVPP in (S)-methyloxirane system. Supplementary Table 3: Information of the original and truncated basis sets of def2-TZVPP in (-)-α-pinene system. Supplementary Table 4: Scaling information of (-)-α-pinene using HF/def2-TZVPP and its truncated basis set. Supplementary Table 5: Computational time of Coulomb and exchange matrices in (-)-α-pinene system using HF/def2-TZVPP and its truncated basis set. Supplementary Table 6: Steps of the CBS scheme for 6-31G(d,p) basis set in ZnPc system. Supplementary Tables 7-8: Number of ERIs and ideal time saving after Schwarz screening with different thresholds in H_2O dimer RT-TDHF calculations using the original and truncated def2-TZVPPD basis sets. Supplementary Table 9: Benchmark of different Schwarz screening thresholds for (S)-methyloxirane RT-TDDFT calculations using def2-TZVPP basis set.

Supplementary Note 2

Geometry Information

Cartesian coordinates. Unit: Å

H_2 dimer

H -1.0000000 0.0000000 -0.4500000

H -1.0000000 0.0000000 0.4500000

H 1.0000000 0.0000000 -0.4500000

H 1.0000000 0.0000000 0.4500000

H_2O dimer

O 0.0000000 1.5177320 -0.0003700

H 0.0000000 0.5573140 0.0967050

H 0.0000000 1.8530790 0.8971800

O 0.0000000 -1.3921280 -0.0003700

H 0.7601590 -1.7076120 -0.4939800

H -0.7601590 -1.7076120 -0.4939800

S-methyloxirane

O 6.9325093673 4.9912922342 5.5530605377

C 4.5936253666 5.9013394963 5.6844889902

H 4.6832066666 6.1468423282 4.6170076972

H 4.0123008910 4.9719106294 5.7766627582

H 4.0311157797 6.7056772937 6.1832016468

C 5.9537764115 5.7423152569 6.3079077528

H 5.9611073223 5.5054747206 7.3803825305

C 7.1510363025 6.4016163228 5.7601842322

H 7.0505597907 7.0270287426 4.8652529700

H 7.9870211972 6.6572293579 6.4210291422

(-)-α-pinene

C 6.2970643042 6.8977858223 7.7552835862

C 6.5122185949 8.3807894545 7.2293822457

C 7.4959507347 6.5407050206 6.7619290537

C 7.0273492785 7.7604056126 5.8890015843

C 7.7073199375 9.0612570693 7.9420692382

C 8.8376389742 6.9200795918 7.3880878581

C 4.9537427076 6.2975771189 7.2841864931

C 6.4517405095 6.6029763575 9.2554974386

C 8.9248800075 8.1471324503 7.9297669936

C 9.9636383640 5.9173847681 7.3613808859

H 5.6317098940 9.0342049032 7.1981797339

H 7.5034066475 5.5413021423 6.3125760262

H 7.8133228040 8.3045717371 5.3561636473

H 6.2220845680 7.5122092933 5.1945919914

H 7.4406928994 9.3248418615 8.9779909072

H 7.9396923997 10.0158911904 7.4439124807

H 4.1208818168 6.7416970350 7.8462501916

H 4.9337845091 5.2150910976 7.4682262820

H 4.7540693064 6.4559173914 6.2203464401

H 6.3951758276 5.5210824890 9.4390370326

H 5.6351096411 7.0712733551 9.8221277538

H 7.3988453120 6.9605845597 9.6654760928

H 9.8499299601 8.5015012465 8.3839403195

H 10.2130575373 5.6270787754 6.3299507350

H 9.6800313436 4.9920780195 7.8846509264

H 10.8692977134 6.3135115345 7.8338880941

ZnPc

Zn 0.000000000 0.000000000 0.000000000

N 0.000000000 3.382794340 0.000000000

N 1.407797620 1.407797620 0.000000000

N 3.382794340 0.000000000 0.000000000

N 1.407797620 -1.407797620 0.000000000

C 1.176447460 2.761717200 0.000000000

C 2.460457550 3.458046190 0.000000000

C 2.795785370 4.812939910 0.000000000

C 4.150231140 5.146435770 0.000000000

C 5.146435770 4.150231140 0.000000000

C 4.812939910 2.795785370 0.000000000

C 3.458046190 2.460457550 0.000000000

C 2.761717200 1.176447460 0.000000000

C 2.761717200 -1.176447460 0.000000000

C 3.458046190 -2.460457550 0.000000000

C 4.812939910 -2.795785370 0.000000000

C 5.146435770 -4.150231140 0.000000000

C 4.150231140 -5.146435770 0.000000000

C 2.795785370 -4.812939910 0.000000000

C 2.460457550 -3.458046190 0.000000000

C 1.176447460 -2.761717200 0.000000000

N -1.407797620 -1.407797620 0.000000000

N -1.407797620 1.407797620 0.000000000

C -1.176447460 2.761717200 0.000000000

N 0.000000000 -3.382794340 0.000000000

C -1.176447460 -2.761717200 0.000000000

C -2.761717200 -1.176447460 0.000000000

C -2.761717200 1.176447460 0.000000000

C -2.460457550 3.458046190 0.000000000

C -2.460457550 -3.458046190 0.000000000

N -3.382794340 0.000000000 0.000000000

C -3.458046190 -2.460457550 0.000000000

C -3.458046190 2.460457550 0.000000000

C -2.795785370 4.812939910 0.000000000

C -2.795785370 -4.812939910 0.000000000

C -4.812939910 -2.795785370 0.000000000

C -4.812939910 2.795785370 0.000000000

C -4.150231140 5.146435770 0.000000000

C -4.150231140 -5.146435770 0.000000000

C -5.146435770 -4.150231140 0.000000000

C -5.146435770 4.150231140 0.000000000

H -2.021772570 5.574520330 0.000000000

H -4.445152790 6.192512250 0.000000000

H -6.192512250 4.445152790 0.000000000

H -5.574520330 2.021772570 0.000000000

H 2.021772570 5.574520330 0.000000000

H 4.445152790 6.192512250 0.000000000

H 6.192512250 4.445152790 0.000000000

H 5.574520330 2.021772570 0.000000000

H 5.574520330 -2.021772570 0.000000000

H 6.192512250 -4.445152790 0.000000000

H 4.445152790 -6.192512250 0.000000000

H 2.021772570 -5.574520330 0.000000000

H -2.021772570 -5.574520330 0.000000000

H -4.445152790 -6.192512250 0.000000000

H -6.192512250 -4.445152790 0.000000000

H -5.574520330 -2.021772570 0.000000000

Ag_{20}

Ag -1.105884 1.105884 1.105884

Ag -0.981190 -0.981190 3.074906

Ag 0.981190 3.074906 0.981190

Ag -3.074906 0.981190 -0.981190

Ag 0.981190 0.981190 3.074906

Ag -3.074906 -0.981190 0.981190

Ag -0.981190 3.074906 -0.981190

Ag 2.954125 2.954125 2.954125

Ag -2.954125 2.954125 -2.954125

Ag -2.954125 -2.954125 2.954125

Ag 3.074906 0.981190 0.981190

Ag 1.105884 -1.105884 1.105884

Ag 3.074906 -0.981190 -0.981190

Ag -0.981190 -3.074906 0.981190

Ag 0.981190 -3.074906 -0.981190

Ag 2.954125 -2.954125 -2.954125

Ag 1.105884 1.105884 -1.105884

Ag 0.981190 -0.981190 -3.074906

Ag -0.981190 0.981190 -3.074906

Ag -1.105884 -1.105884 -1.105884

Equation

Standard deviation of a set of complex numbers, where N_t is the size of the set.

$$S_t[x(t)] = \sqrt{\frac{1}{N_t}\sum_t |x(t) - \bar{x}|^2}, \text{where } \bar{x} = \frac{1}{N_t}\sum_t x(t),\ \ x(t) \in \mathbb{C} \tag{1}$$

Supplementary Note 3

Figures

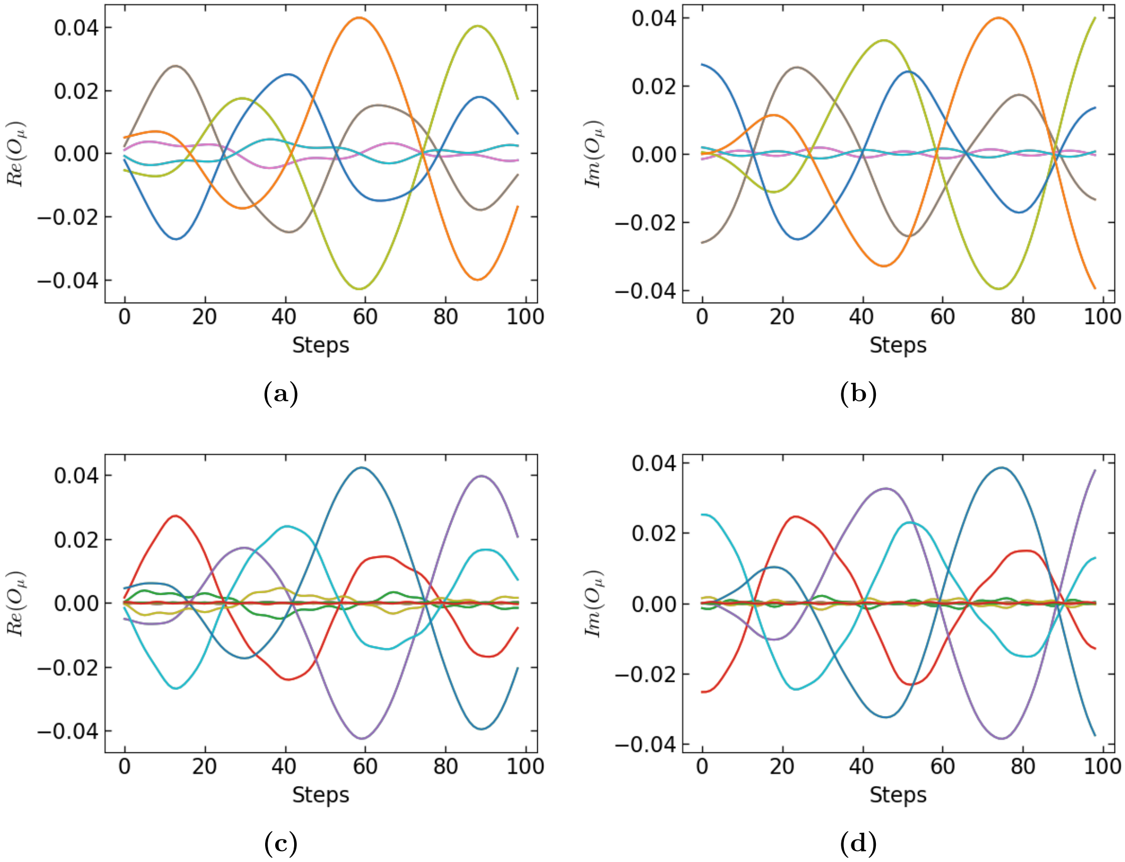

Supplementary Figure 1. O_μ of H_2 dimer in the first 100 RTP steps. (a) real part of O_μ using 6-31++G basis set, (b) imaginary part of O_μ using 6-31++G basis set, (c) real part of O_μ using 6-31++G** basis set, and (d) imaginary part of O_μ using 6-31++G** basis set. All lines are shifted to so that mean values are 0. Each line represent the contribution from a basis function. Note that some basis functions overlap with each other due to the symmetry of the system.

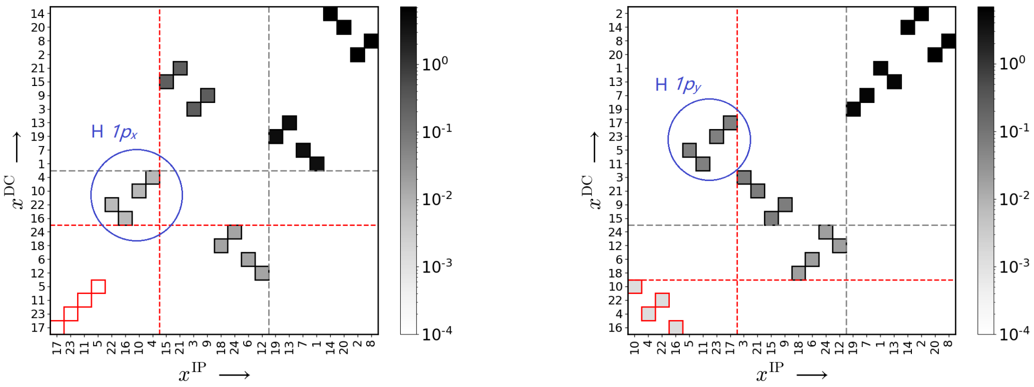

(a) **(b)**

Supplementary Figure 2. x^{DC}-x^{IP} map of H_2 dimer obtained with 6-31++G** basis set and delta pulse from (a) x and (b) y directions. Each square in the map represents a basis function with its numbering on the x and y axes. The color of the squares is based on the value calculated as $x^{\mathrm{DC}} \cdot x^{\mathrm{IP}}$, so the deeper the color the more important the basis function is in the TDHF calculation. Axis labels (basis functions) are sorted in ascending order according to their x^{DC} or x^{IP} values. The red dashed line represents $x^{\mathrm{thr}} = 0.1$ and the grey dashed line represents $x^{\mathrm{thr}} = 0.2$.

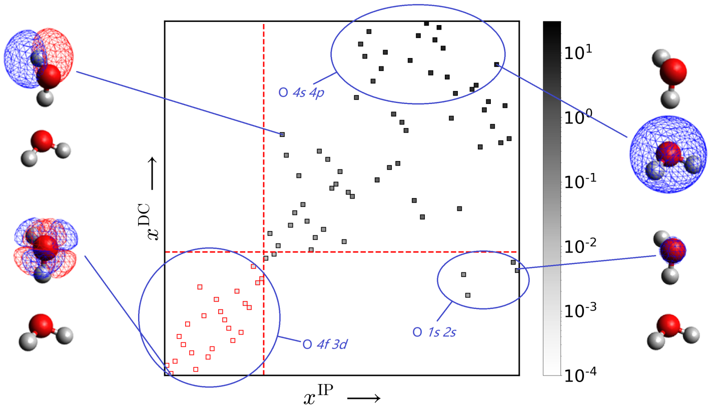

Supplementary Figure 3. x^{DC}-x^{IP} map of H_2O dimer system obtained with def2-TZVP basis set and the visualization of related basis functions.

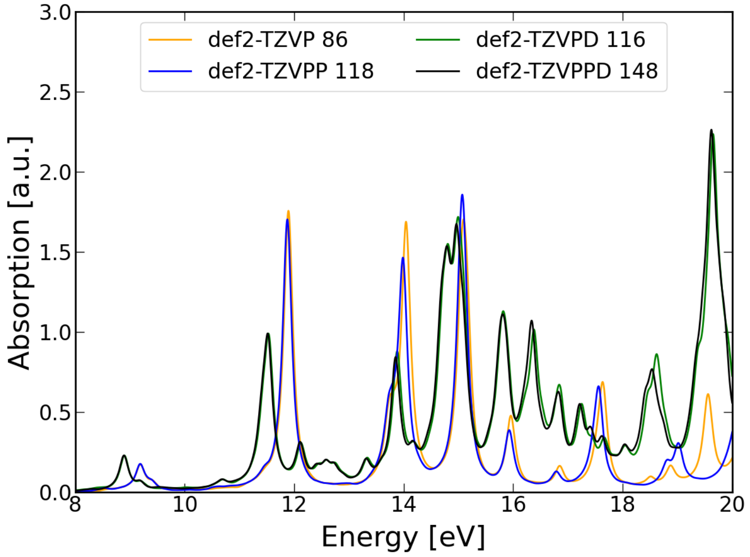

Supplementary Figure 4. H_2O dimer RT-TDHF spectra using def2-TZVP, def2-TZVPP, def2-TZVPD, and def2-TZVPPD basis sets. The number after the basis set label is the total number of basis functions used for the RTP. a.u.: arbitrary units.

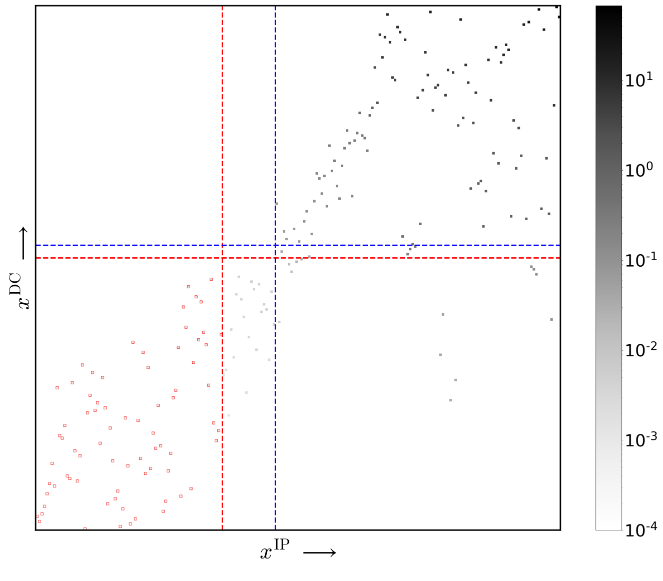

Supplementary Figure 5. x^{DC}-x^{IP} map of (S)-methyloxirane using def2-TZVPP basis set. Each square in the map represent a basis function with its numbering on the x and y axes. The color of squares is based on the value calculated as $x^{\mathrm{DC}} \cdot x^{\mathrm{IP}}$, so the deeper the color the more important the basis function is in the TDDFT calculation. The red dashed line represents $x^{\mathrm{thr}} = 0.1$, and the blue dashed line represents $x^{\mathrm{thr}} = 0.2$

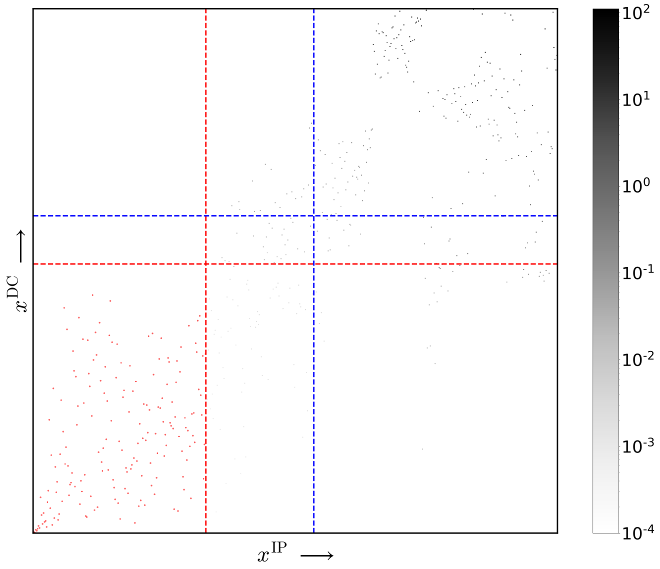

Supplementary Figure 6. x^{DC}-x^{IP} map of (-)-α-pinene using def2-TZVPP basis set. Each square in the map represents a basis function with its numbering on the x and y axes. The color of squares is based on the value calculated as $x^{DC} \cdot x^{IP}$, so the deeper the color the more important the basis function is in the TDDFT calculation. The red dashed line represents $x^{thr} = 0.1$, and the blue dashed line represents $x^{thr} = 0.2$

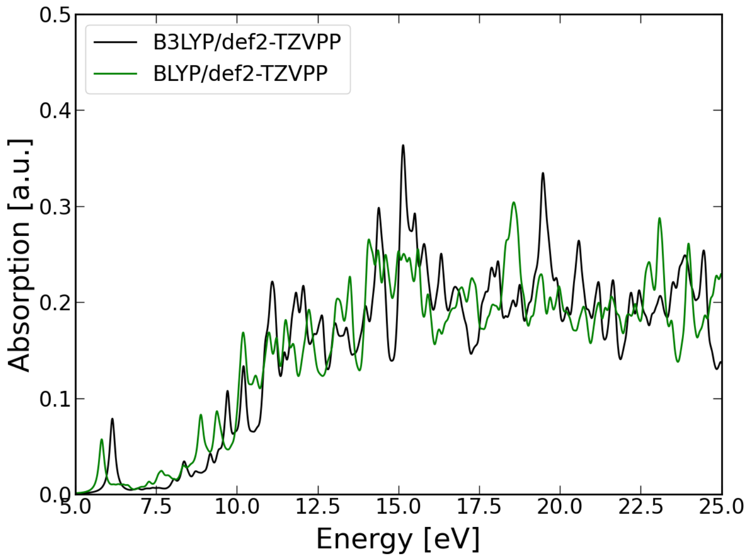

Supplementary Figure 7. (-)-α-pinene RT-TDDFT electronic absorption spectra using B3LYP and BLYP functional and def2-TZVPP basis set. a.u.: arbitrary units.

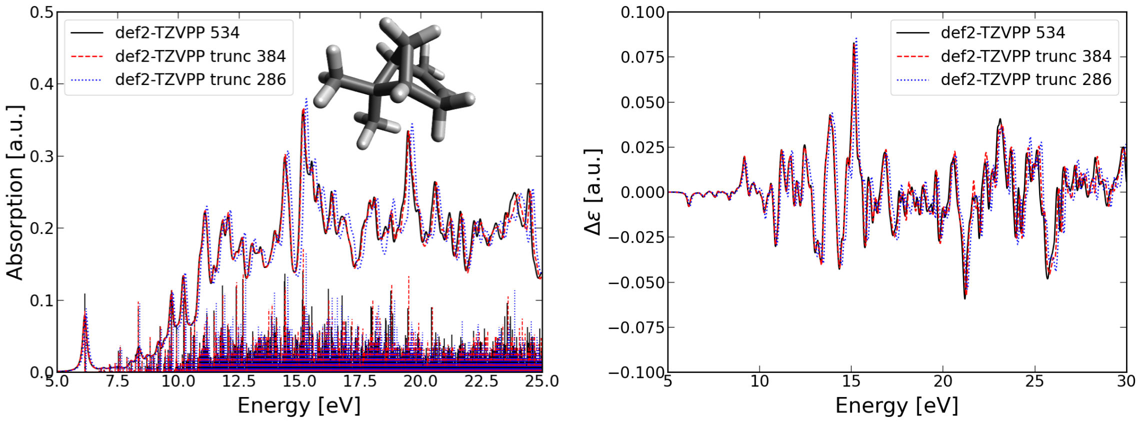

(a) **(b)**

Supplementary Figure 8. (-)-α-pinene (a) electronic absorption spectra and (b) electronic circular dichroism spectra using original and truncated basis sets of def2-TZVPP (stick spectra below correspond to LR-TDDFT ones). The truncation thresholds are $x^{\text{thr}} = 0.1$ and $x^{\text{thr}} = 0.2$. The number after the basis set label indicates the number of basis functions. a.u.: arbitrary units.

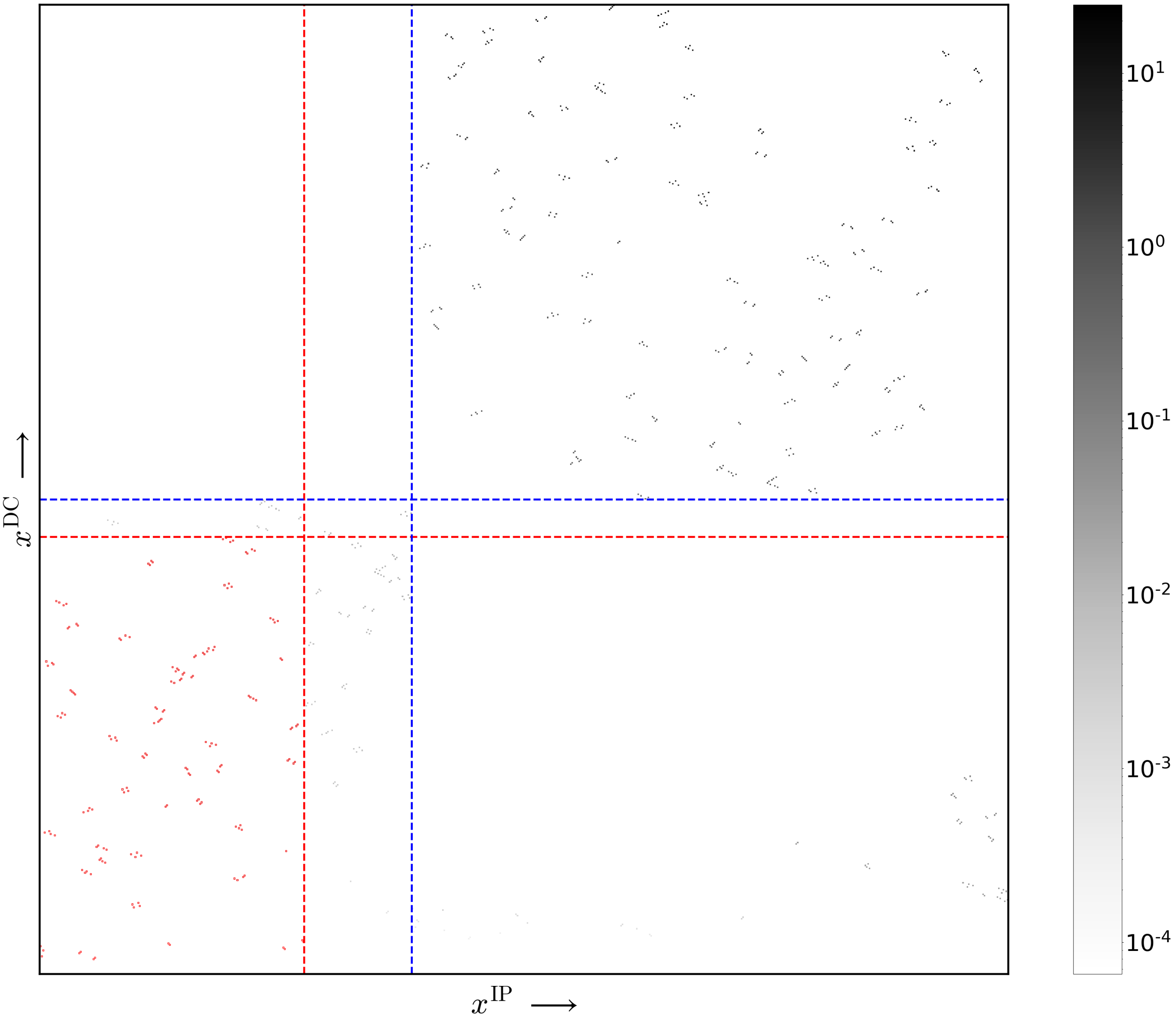

Supplementary Figure 9. x^{DC}-x^{IP} map of ZnPc using 6-31G(d,p) basis set. Each square in the the map represent a basis function with its numbering on x and y axes. The color of squares is based on the value calculated as $x^{\mathrm{DC}} \cdot x^{\mathrm{IP}}$, so the deeper the color the more important the basis function is in the TDDFT calculation. The red dashed line represents $x^{\mathrm{thr}} = 0.09$, and the blue dashed line represents $x^{\mathrm{thr}} = 0.18$

Tables

Supplementary Table 1. The steps of the CBS scheme for def2-QZVPPD basis set in H_2O dimer system. The truncation threshold used in the CBS scheme is $x^{\text{thr}} = 0.1$. Basis set information of H and O atoms is noted as *non-italic* form, and the added/truncated/removed basis functions are in *italic* form in the brackets. "Run" refers to a short time RT-TDHF calculation with 100 steps. In the last column, the total number of basis functions of the system is listed. Note that the first two RT-TDHF runs are used for the truncation. It is because the remaining $1f$ of O is half truncated (e.g. some basis functions are suggested to be truncated) in the first run and it is the only one in f-subshell, thus whether to truncate this will determine whether additional diffuse functions can enter the f-subshell.

Basis set	H	O	No. basis function
Original	4s4p2d1f	8s5p4d2f1g	264
	Run 1		
Truncate	4s3p1d (-1*p*1*d*1*f*)	8s5p3d1f (-1*d*1*f*1*g*)	162
	Run 2		
Truncate	4s3p1d	8s5p3d (-1*f*)	148
Add	5s4p2d (+5*s*4*p*2*d*)	9s6p4d (+9*s*6*p*4*d*)	202
	Run 3		
Truncate	5s4p1d (-1*d*)	8s6p3d (-2*s*1*d*)	170
Add	6s5p2d (+6*s*5*p*2*d*)	9s7p4d (+9*s*7*p*4*d*)	224
	$\lvert\lambda\rvert_{min} < 10^{-6}$		
Remove	6s5p1d (-2*d*)	9s7p3d (-4*d*)	194
	Run 4		
Truncate	6s4p1d (-1*p*)	9s7p3d	182
	$\lvert\lambda\rvert_{min} = 1.0 \times 10^{-6}$		

Supplementary Table 2. Information of the original and truncated basis sets of def2-TZVPP in (S)-methyloxirane system. The truncation threshold used are $x^{\text{thr}} = 0.1$ and $x^{\text{thr}} = 0.2$. Basis set information of H, C, and O atoms is noted as *non-italic* form, and the truncated basis functions are in *italic* form in the brackets. In the last column, number of basis functions of the system is listed.

Basis set	H	C	O	No. basis function
Def2-TZVPP	3s2p1d	5s3p2d1f	5s3p2d1f	208
Trunc $x^{\text{thr}} = 0.1$	3s2p (-1*d*)	5s3p2d (-1*f*)	5s3p1d (-1*d*1*f*)	145
Trunc $x^{\text{thr}} = 0.2$	3s1p (-1*p*1*d*)	5s3p1d (-1*d*1*f*)	5s3p1d (-1*d*1*f*)	112

Supplementary Table 3. Information of the original and truncated basis sets of def2-TZVPP in (-)-α-pinene system. The truncation threshold used are $x^{\text{thr}} = 0.1$ and $x^{\text{thr}} = 0.2$. Basis set information of H and C atoms is noted as *non-italic* form, and the truncated basis functions are in *italic* form in the brackets. In the last column, number of basis functions of the system is listed.

Basis set	H	C	No. basis function
Def2-TZVPP	3s2p1d	5s3p2d1f	534
Trunc $x^{\text{thr}} = 0.1$	3s2p (-1*d*)	5s3p2d (-1*f*)	384
Trunc $x^{\text{thr}} = 0.2$	3s1p (-1*p*1*d*)	5s3p1d (-1*d*1*f*)	286

Supplementary Table 4. Scaling information of (-)-α-pinene using HF/def2-TZVPP and its truncated basis set ($x^{\text{thr}} = 0.2$). The total number of basis functions are 534 and 286, respectively. Each computational node has 12 CPU cores@2.6 GHz and 64 GB memory. Original basis with less than 8 nodes is memory-bound.

No. nodes	1	2	4	8	16	32	64
Def2-TZVPP: Time/Step [s]	480	205	56	18	11	6.5	3.3
Trunc $x^{\text{thr}} = 0.2$: Time/Step [s]	34	17	9.6	4.7	2.7	1.7	2.5

Supplementary Table 5. Computational time for calculation of Coulomb and exchange matrices in one step for (-)-α-pinene system using HF/def2-TZVPP and its truncated basis set ($x^{\text{thr}} = 0.2$). The total number of basis functions are 534 and 286, respectively. 8 computational nodes are used for the calculation with each node 12 CPU cores@2.6 GHz and 64 GB memory.

Time/Step [s]	Coulomb	Exchange
Def2-TZVPP	7.7	5.4
Trunc $x^{\text{thr}} = 0.2$	2.1	0.8

Supplementary Table 6. Steps of the CBS scheme for 6-31G(d,p) basis set in ZnPc system. The truncation threshold used in the CBS scheme is $x^{\text{thr}} = 0.1$. Basis set information of each type of atom is noted as *non-italic* form, and the added/truncated/removed basis functions are in *italic* form in the brackets. According to Algorithm 1, this CBS scheme can stop at No. basis function = 884 or 680 (due to the ϵ-condition), but it is also possible to carry on the calculation until no additional basis functions can be added to any subshells. **Bold** font denotes the certain subshell is fixed since additional basis functions are either truncated or removed in previous steps. "Run" refers to a short time RT-TDDFT calculation with 100 steps. In the last column, the total number of basis functions of the system is listed.

Basis set	H	C	N	Zn	No. basis function
Original	2s1p	3s2p1d	3s2p1d	5s4p2d1f	674
			Run 1		
Truncate	2s (-1*p*)	3s2p1d	3s2p1d	5s4p2d (-1*f*)	619
Add	3s (+3*s*)	4s3p2d (+4*s*3*p*2*d*)	4s3p2d (+4*s*3*p*2*d*)	6s5p3d (+6*s*5*p*3*d*)	1004
			$\lvert\lambda\rvert_{min} < 10^{-6}$		
Remove	3s	4s**2p**2d (-3*p*)	4s**2p**2d (-3*p*)	6s5p3d	884
			Run 2		
Truncate	3s	4s**2p**1d (-1*d*)	4s**2p**1d (-1*d*)	**5s4p**3d ($-6s5p$)	680
Add	4s (+4*s*)	5s**2p**2d (+5*s*2*d*)	5s**2p**2d (+5*s*2*d*)	**5s4p**4d (+4*d*)	941
			$\lvert\lambda\rvert_{min} < 10^{-6}$		
Remove	4s	**4s2p1d** (-5*s*2*d*)	**4s2p1d** (-5*s*2*d*)	**5s4p**4d	701
			Run 3		
Truncate	4s	**4s2p1d**	**4s2p1d**	**5s4p**4d	701
Add	5s (+5*s*)	**4s2p1d**	**4s2p1d**	**5s4p**5d (+5*d*)	722
			$\lvert\lambda\rvert_{min} < 10^{-6}$		
Remove	**4s** (-5*s*)	**4s2p1d**	**4s2p1d**	**5s4p**5d	706
			Run 4		
Truncate	**4s**	**4s2p1d**	**4s2p1d**	**5s4p4d** (-5*d*)	701
			$\lvert\lambda\rvert_{min} = 8.4 \times 10^{-6}$		

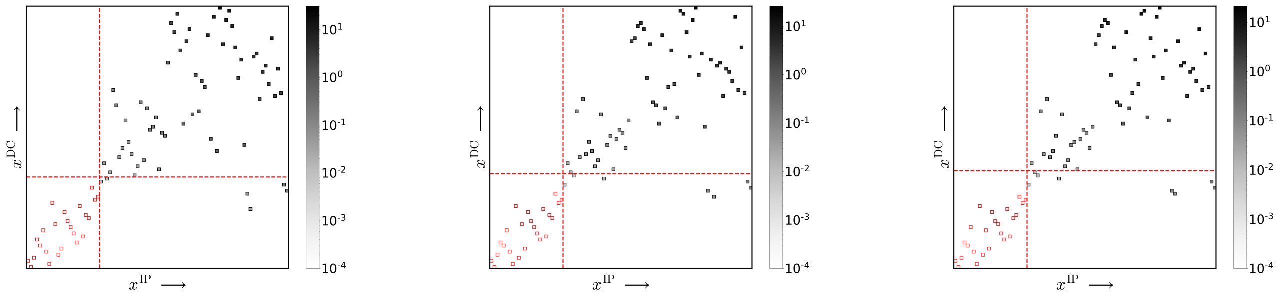

(a) **(b)** **(c)**

Supplementary Figure 10. x^{DC}-x^{IP} map of H_2O dimer using the first (a) 100 steps, (b) 1000 steps and (c) 10000 steps (all steps) in the RT-TDHF calculation with def2-TZVP basis set. The red dashed line represents $x^{thr} = 0.1$. All cases lead to the same truncation suggestion.

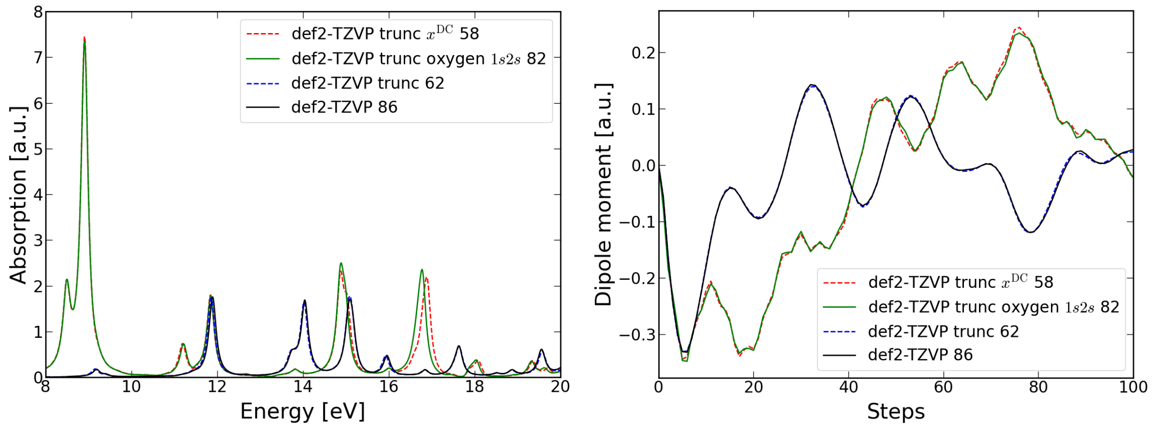

(a) **(b)**

Supplementary Figure 11. H_2O dimer RT-TDHF (a) electronic absorption spectra and (b) dipole moments in the first RTP 100 steps using original and truncated basis sets of def2-TZVP. The truncation threshold is $x^{thr} = 0.1$. The 'def2-TZVP trunc x^{DC} 58' uses only the indicator x^{DC} and truncates additional $1s2s$ basis functions from two oxygen atoms (see Supplementary Figure 3) compared to the 'def2-TZVP trunc 62' basis set. The 'def2-TZVP trunc oxygen $1s2s$ 82' only truncates $1s2s$ from two oxygen atoms compared to the original basis set 'def2-TZVP 86'. The number after the basis set label indicates the number of basis functions. a.u.: arbitrary units.

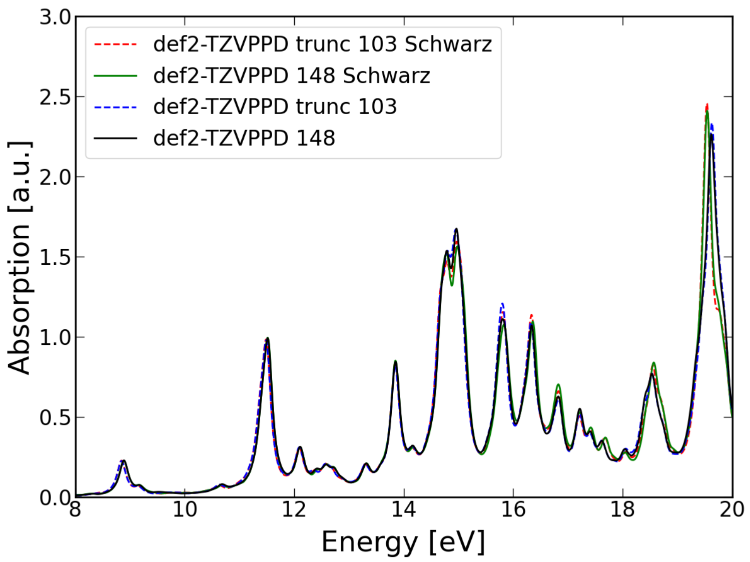

Supplementary Figure 12. H_2O dimer RT-TDHF spectra using original and truncated basis sets of def2-TZVPPD (no Schwarz screening applied), and the same basis sets with the largest Schwarz screening threshold in our tests (10^{-4}). The number after the basis set label indicates the number of basis functions. The ideal time saving of the calculations can be seen in Supplementary Tables 7 and 8. a.u.: arbitrary units.

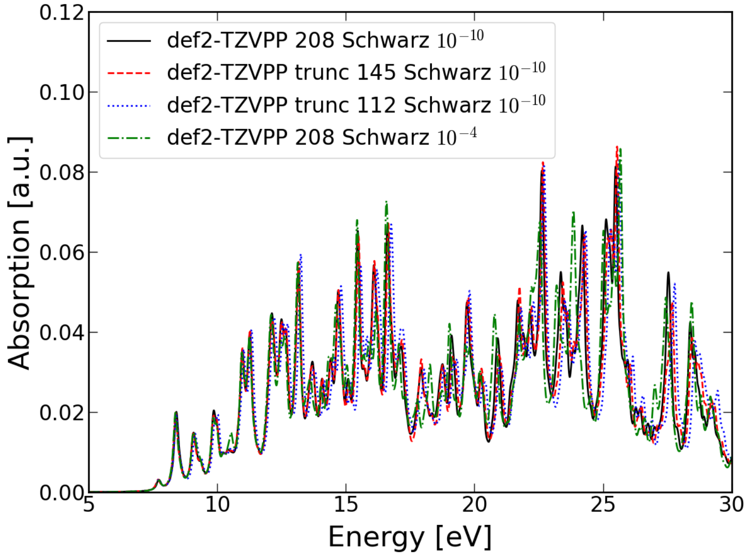

Supplementary Figure 13. (S)-methyloxirane electronic absorption spectra using the original and truncated basis sets of def2-TZVPP, and def2-TZVPP basis set with the largest Schwarz screening threshold in our tests (10^{-4}). The default Schwarz screening threshold in CP2K is 10^{-10}. Starting from the threshold 10^{-6}, the spectra in the energy range [5 eV, 30 eV] are the same with the one obtained with threshold 10^{-10}. The truncation thresholds are $x^{\text{thr}} = 0.1$ and $x^{\text{thr}} = 0.2$. The number after the basis set label indicates the number of basis functions. The benchmark of the calculations can be seen in Supplementary Tables 2 and 9. a.u.: arbitrary units.

Supplementary Table 7. Number of ERIs and ideal time saving after Schwarz screening with different thresholds. The tests are carried out with H_2O dimer RT-TDHF calculations using def2-TZVPPD basis set (PySCF). 10^{-12} is the recommended value in PySCF and 10^{-4} is the largest feasible value from our tests.

Threshold	None	10^{-12}	10^{-6}	10^{-5}	10^{-4}
No. nonzero ERIs	479785216	431086656	332741764	304148676	268552968
Time saving	0 %	10 %	31 %	37 %	44 %

Supplementary Table 8. Number of ERIs and ideal time saving after Schwarz screening with the truncated def2-TZVPPD basis set with 103 basis functions. The tests are carried out with H_2O dimer RT-TDHF calculations (PySCF). The largest feasible value 10^{-4} for the Schwarz screening is applied.

Threshold	None	10^{-4}
No. nonzero ERIs	112550881	77141769
Time saving	0 %	31 %

Supplementary Table 9. Benchmark of different Schwarz screening thresholds for (S)-methyloxirane RT-TDDFT calculations using def2-TZVPP basis set (CP2K). 10^{-10} is the default value in CP2K and 10^{-4} is the largest feasible value from our tests (i.e. RTP scheme cannot converge within 10 iterations for 10^{-3}). Unit: s: second.

Threshold	10^{-12}	10^{-10}	10^{-8}	10^{-6}	10^{-4}
Time/Step	3.82 ± 0.37 s	3.56 ± 0.22 s	3.50 ± 0.36 s	3.30 ± 1.34 s	2.80 ± 1.19 s
