# Supplementary figures and images for: Automatic purpose-driven basis set truncation for time-dependent Hartree–Fock and density-functional theory
Source: Nat Commun. 2023 Jan 6;14:106. doi: 10.1038/s41467-022-35694-4 (PMC9822955; doi:10.1038/s41467-022-35694-4)

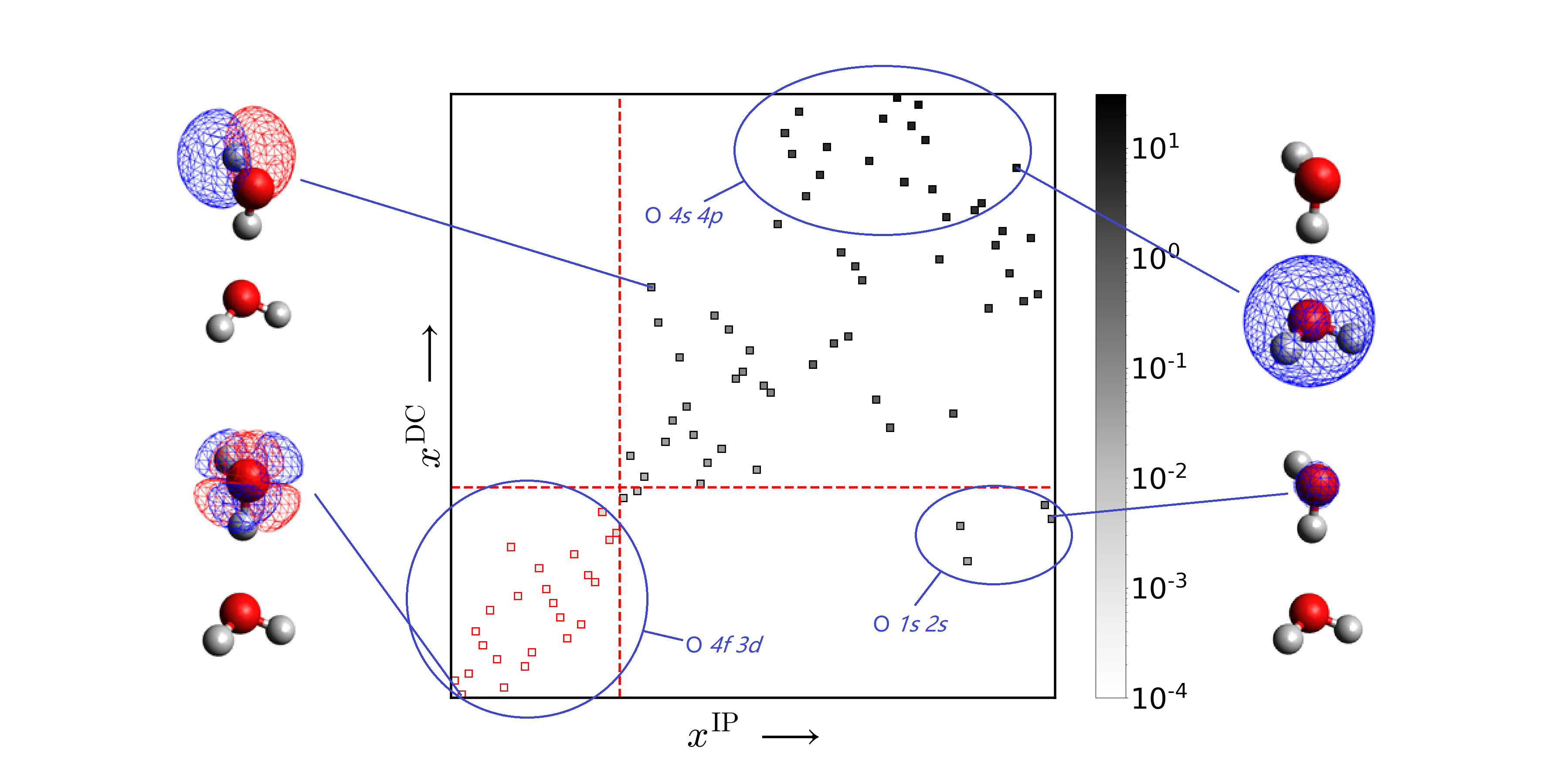

Supplement: Supplementary file 2 — Source Data [file 41467_2022_35694_MOESM2_ESM.zip › figures/h2o_dimer_tzvp_orb.png]

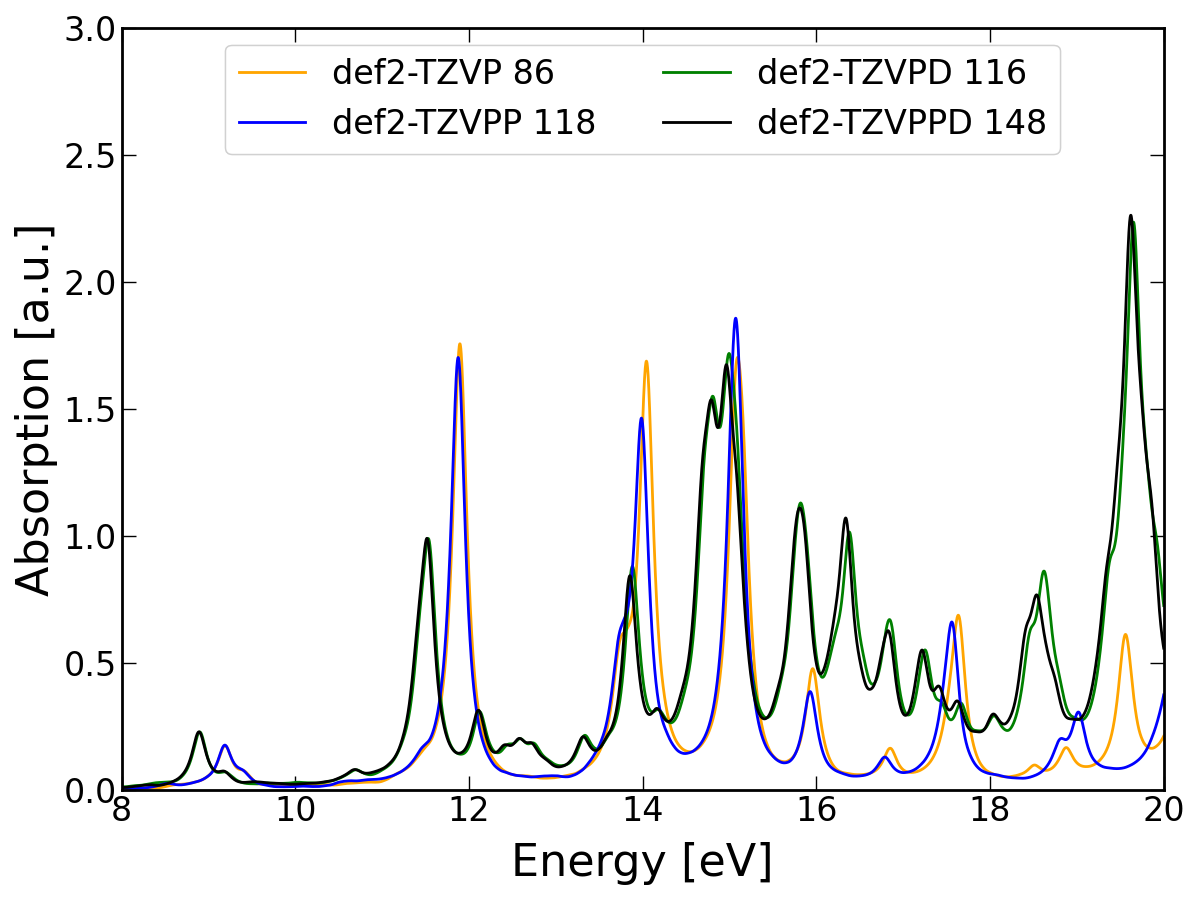

Supplement: Supplementary file 2 — Source Data [file 41467_2022_35694_MOESM2_ESM.zip › figures/h2o_dimer_spectra.png]

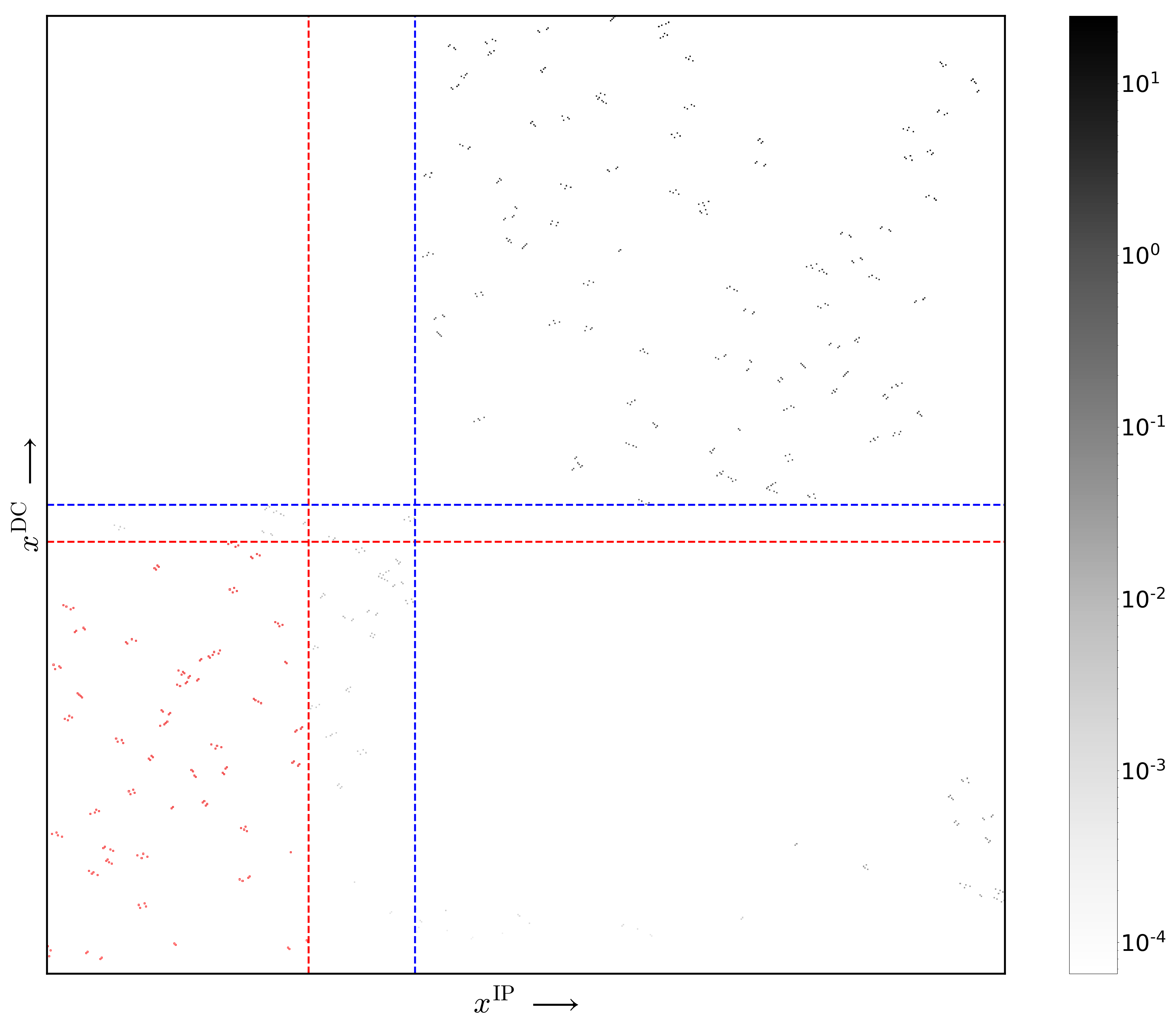

Supplement: Supplementary file 2 — Source Data [file 41467_2022_35694_MOESM2_ESM.zip › figures/znpc_4-orb.png]

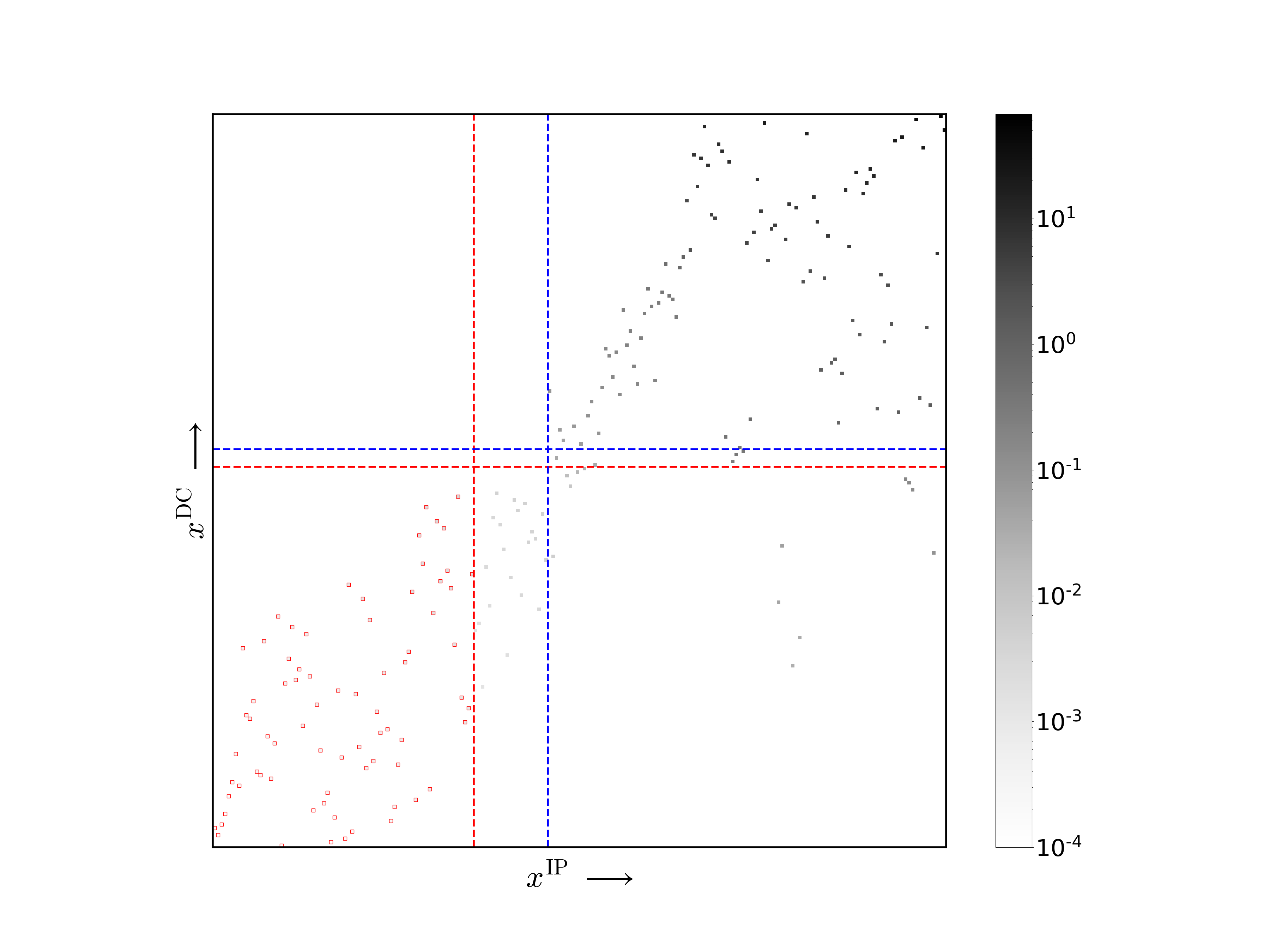

Supplement: Supplementary file 2 — Source Data [file 41467_2022_35694_MOESM2_ESM.zip › figures/c3h6o_4-orb.png]

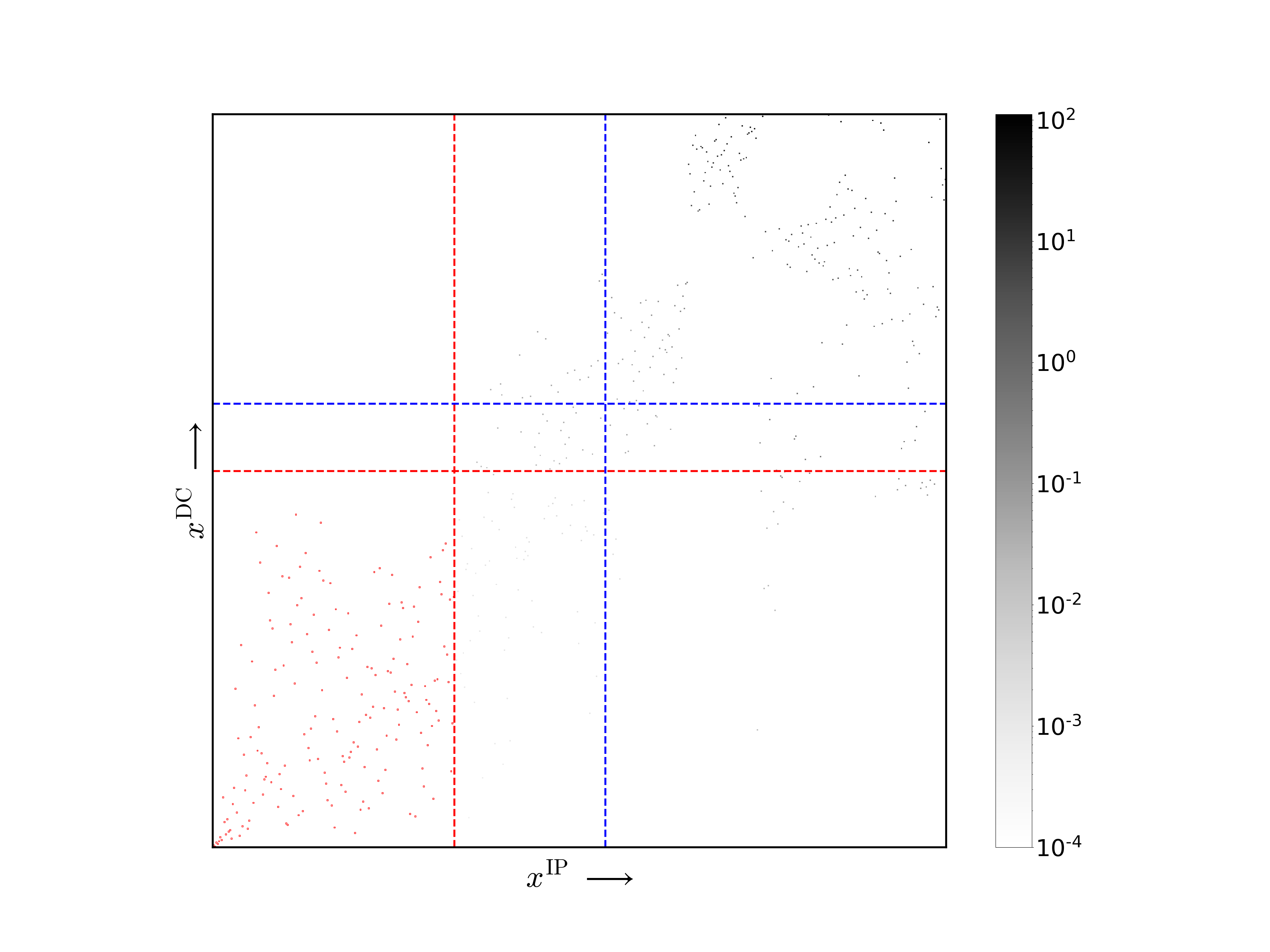

Supplement: Supplementary file 2 — Source Data [file 41467_2022_35694_MOESM2_ESM.zip › figures/pinene_4-orb.png]

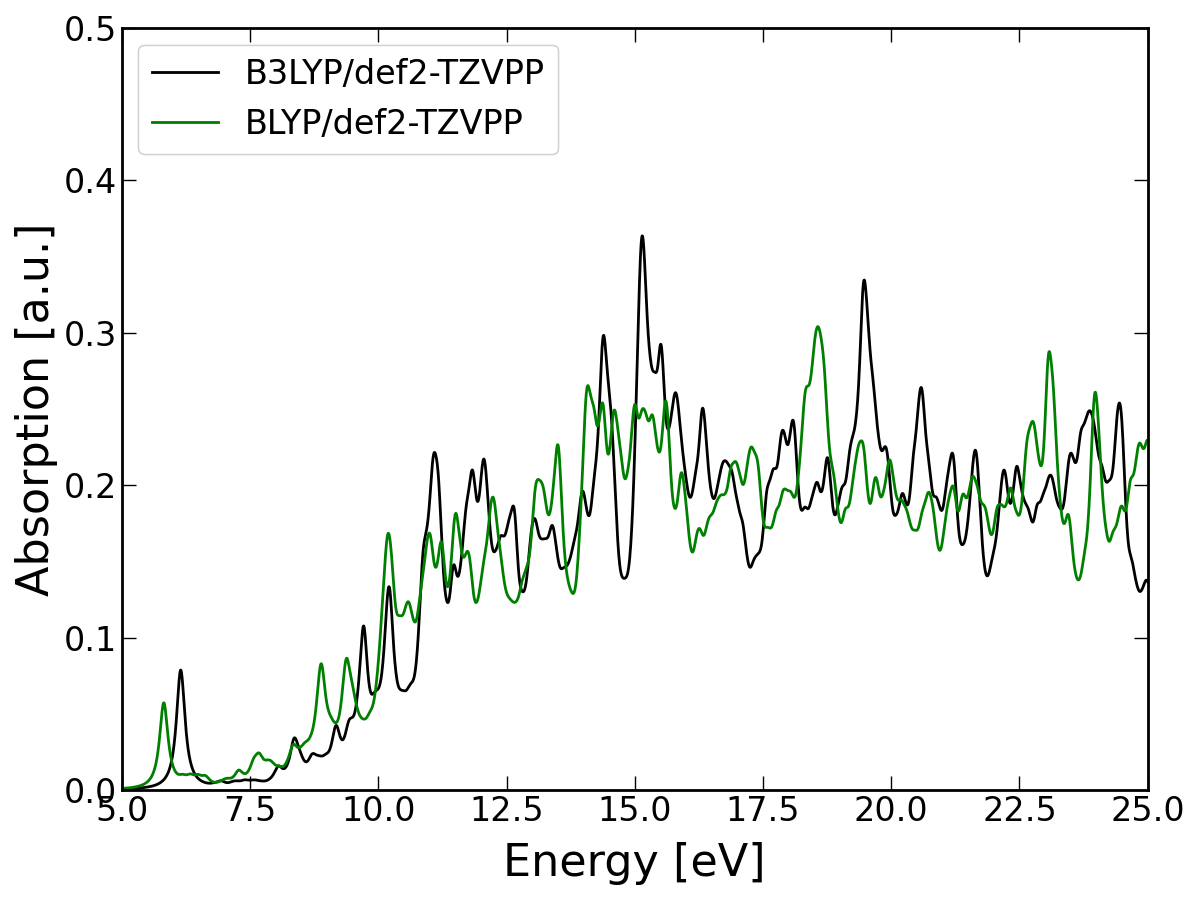

Supplement: Supplementary file 2 — Source Data [file 41467_2022_35694_MOESM2_ESM.zip › figures/pinene_spectra_compare.png]

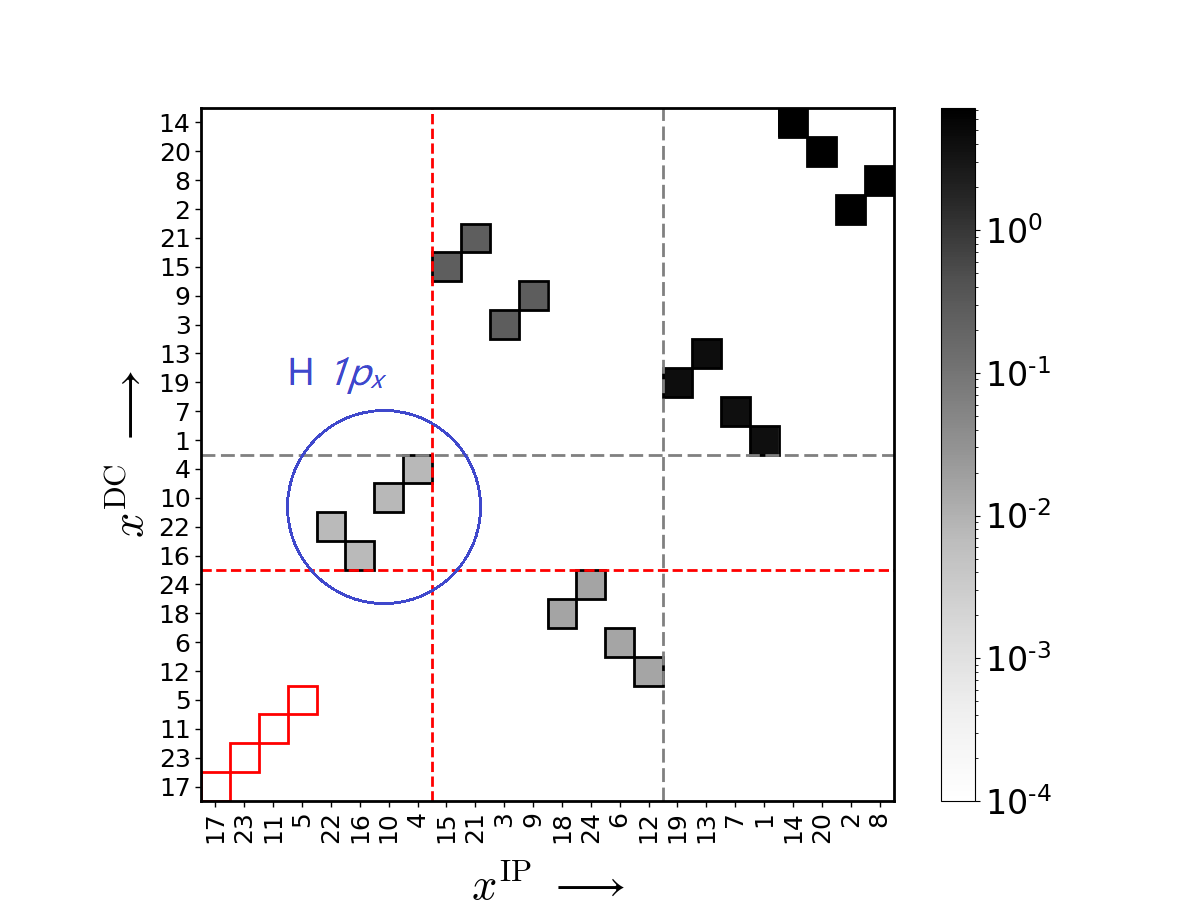

Supplement: Supplementary file 2 — Source Data [file 41467_2022_35694_MOESM2_ESM.zip › figures/h4_2.0_6-31++gpp_x.png]

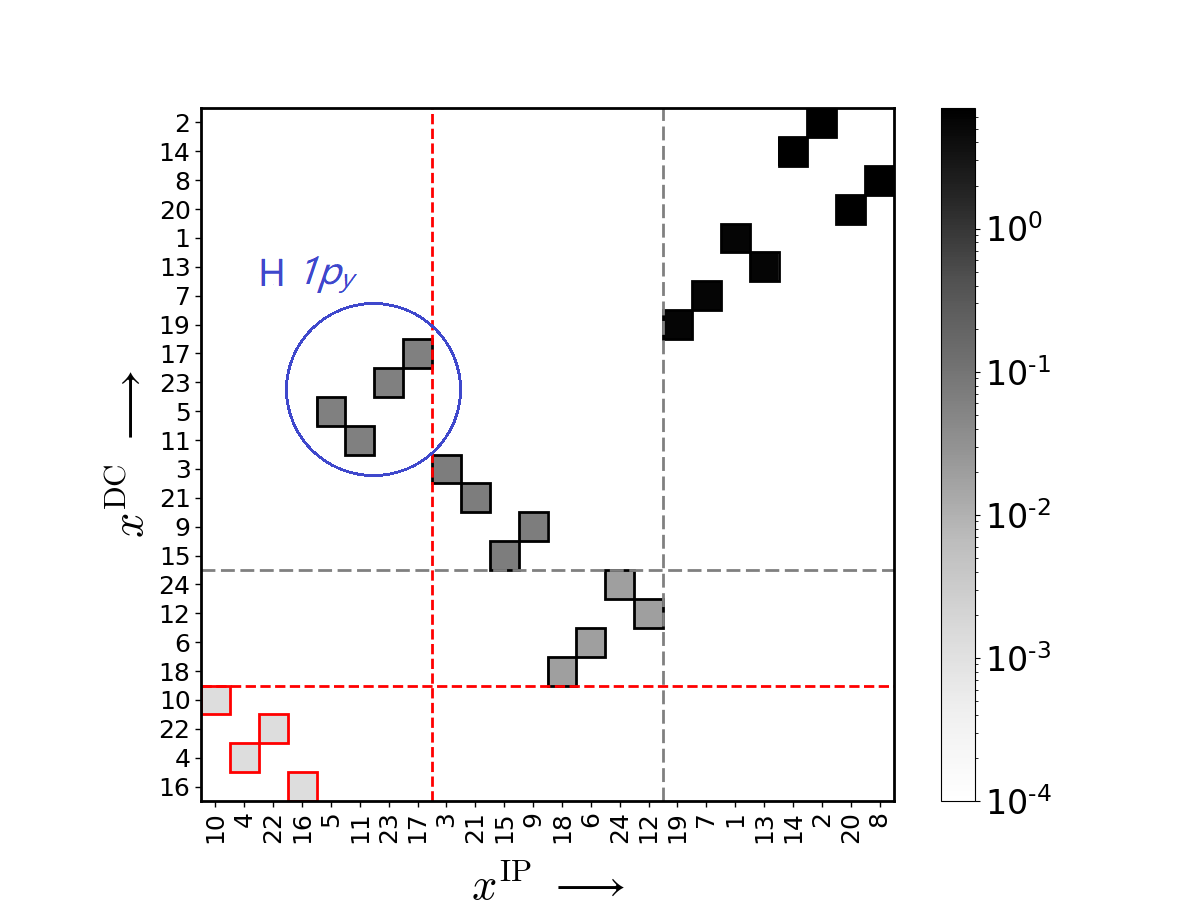

Supplement: Supplementary file 2 — Source Data [file 41467_2022_35694_MOESM2_ESM.zip › figures/h4_2.0_6-31++gpp_y.png]

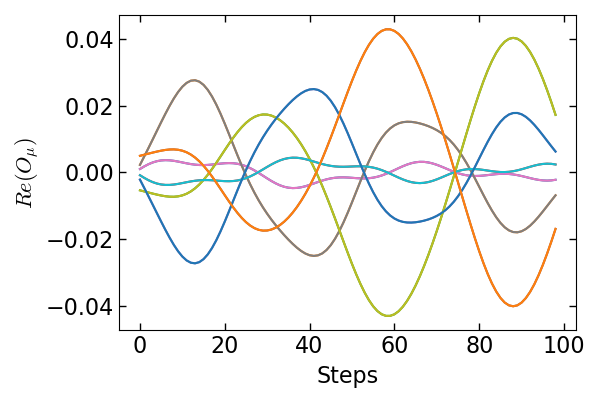

Supplement: Supplementary file 2 — Source Data [file 41467_2022_35694_MOESM2_ESM.zip › figures/h2_dimer_6-31++g_Omu_real.png]

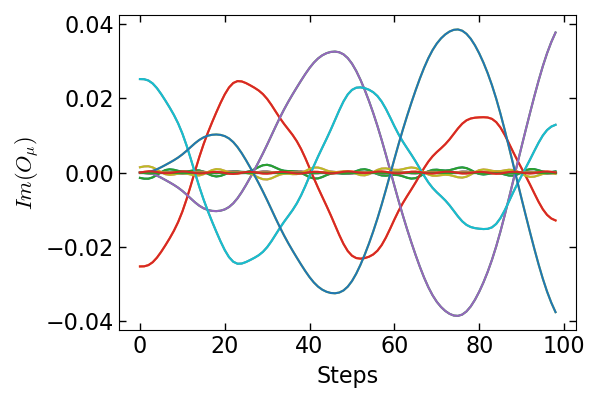

Supplement: Supplementary file 2 — Source Data [file 41467_2022_35694_MOESM2_ESM.zip › figures/h2_dimer_6-31++gpp_Omu_imag.png]

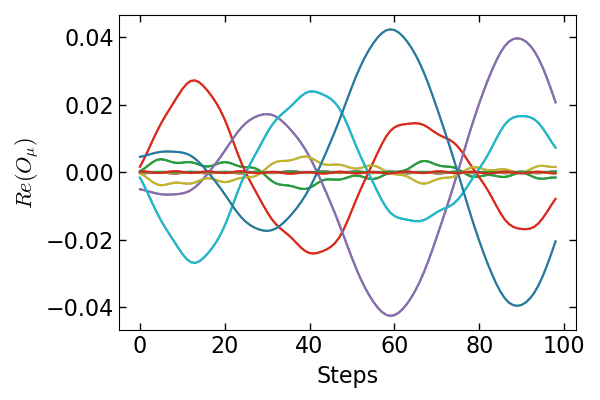

Supplement: Supplementary file 2 — Source Data [file 41467_2022_35694_MOESM2_ESM.zip › figures/h2_dimer_6-31++gpp_Omu_real.png]

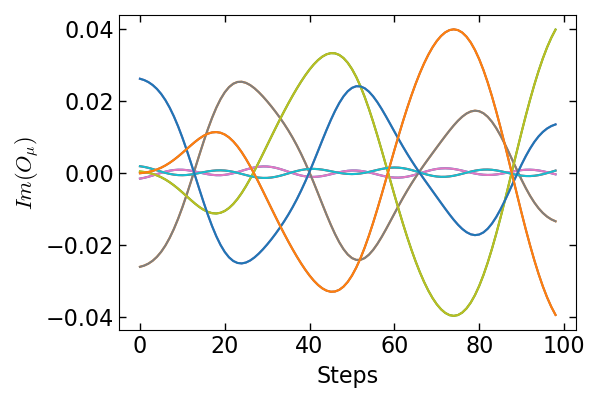

Supplement: Supplementary file 2 — Source Data [file 41467_2022_35694_MOESM2_ESM.zip › figures/h2_dimer_6-31++g_Omu_imag.png]

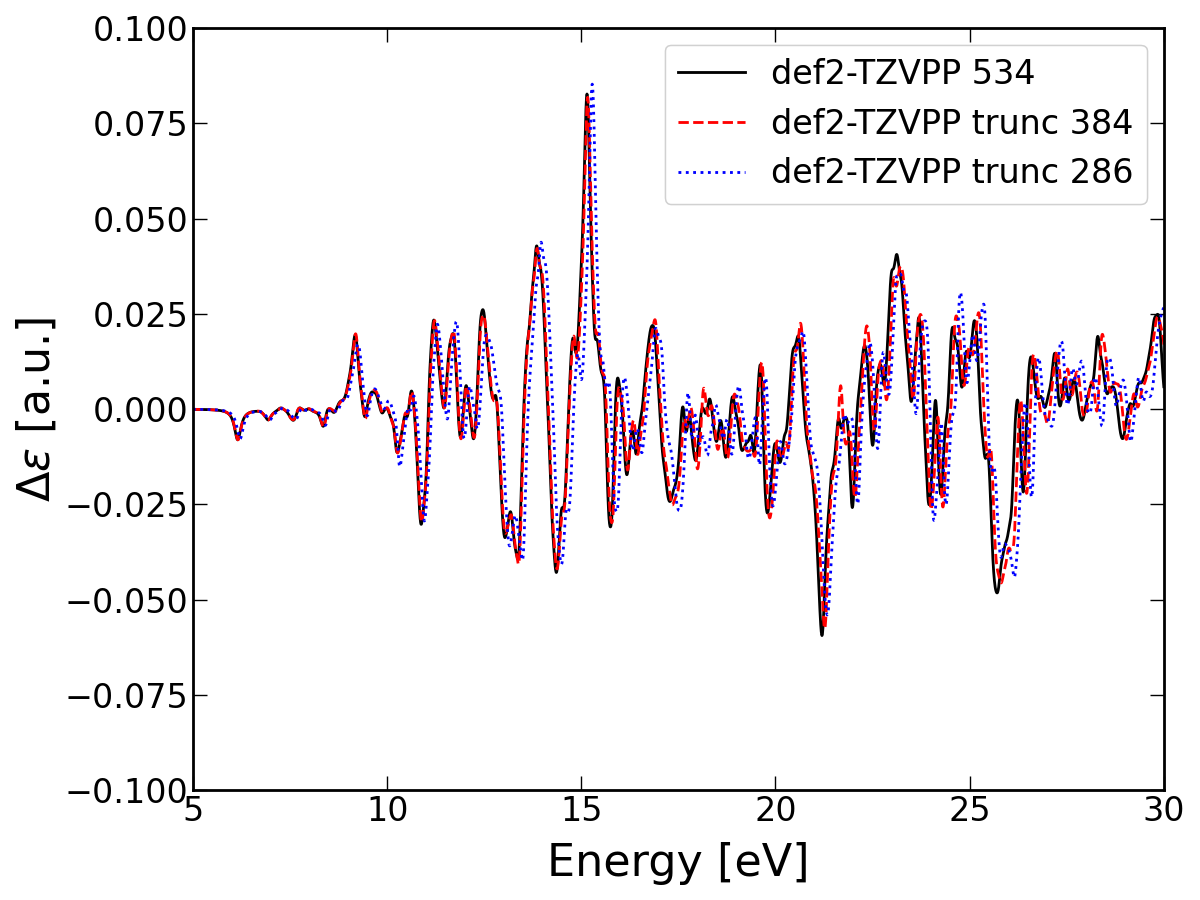

Supplement: Supplementary file 2 — Source Data [file 41467_2022_35694_MOESM2_ESM.zip › figures/pinene_spectra_mag.png]

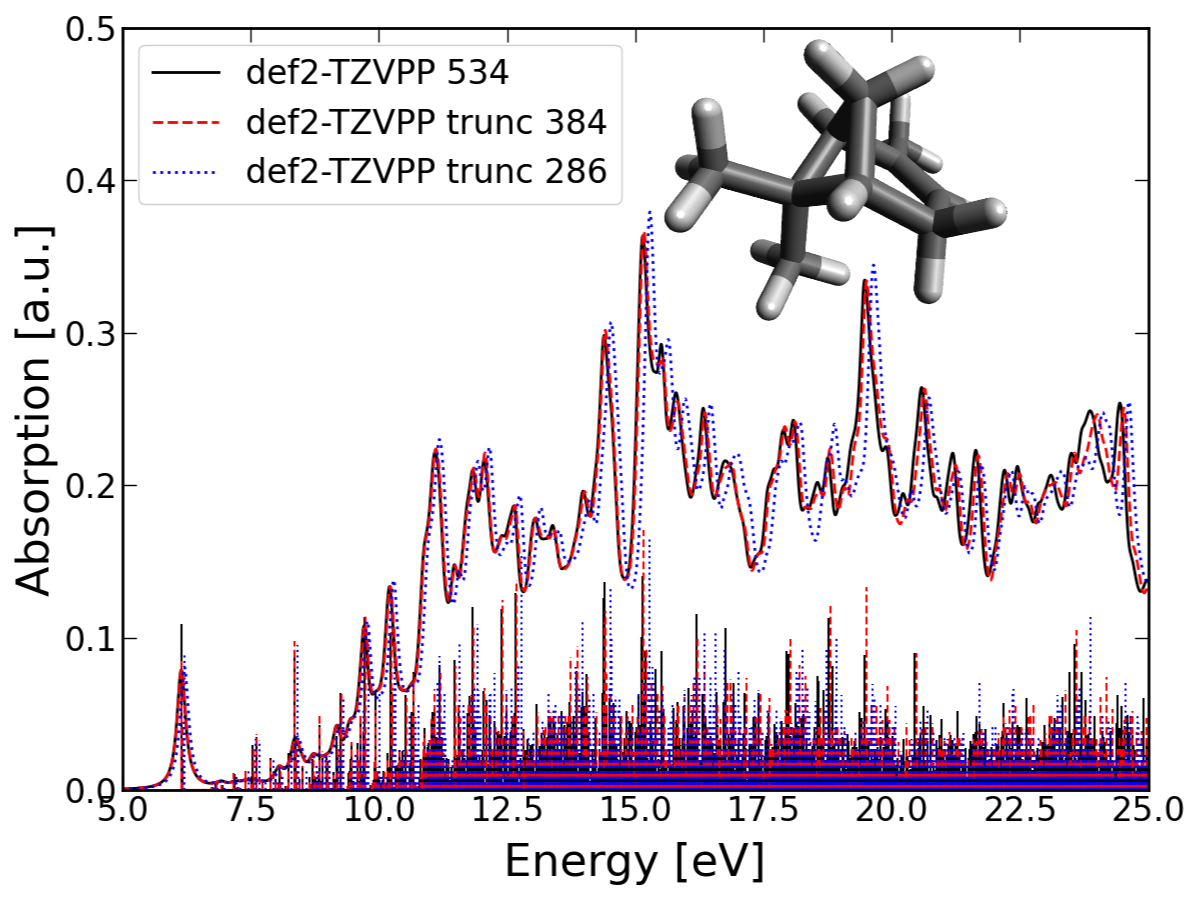

Supplement: Supplementary file 2 — Source Data [file 41467_2022_35694_MOESM2_ESM.zip › figures/pinene_spectra.png]

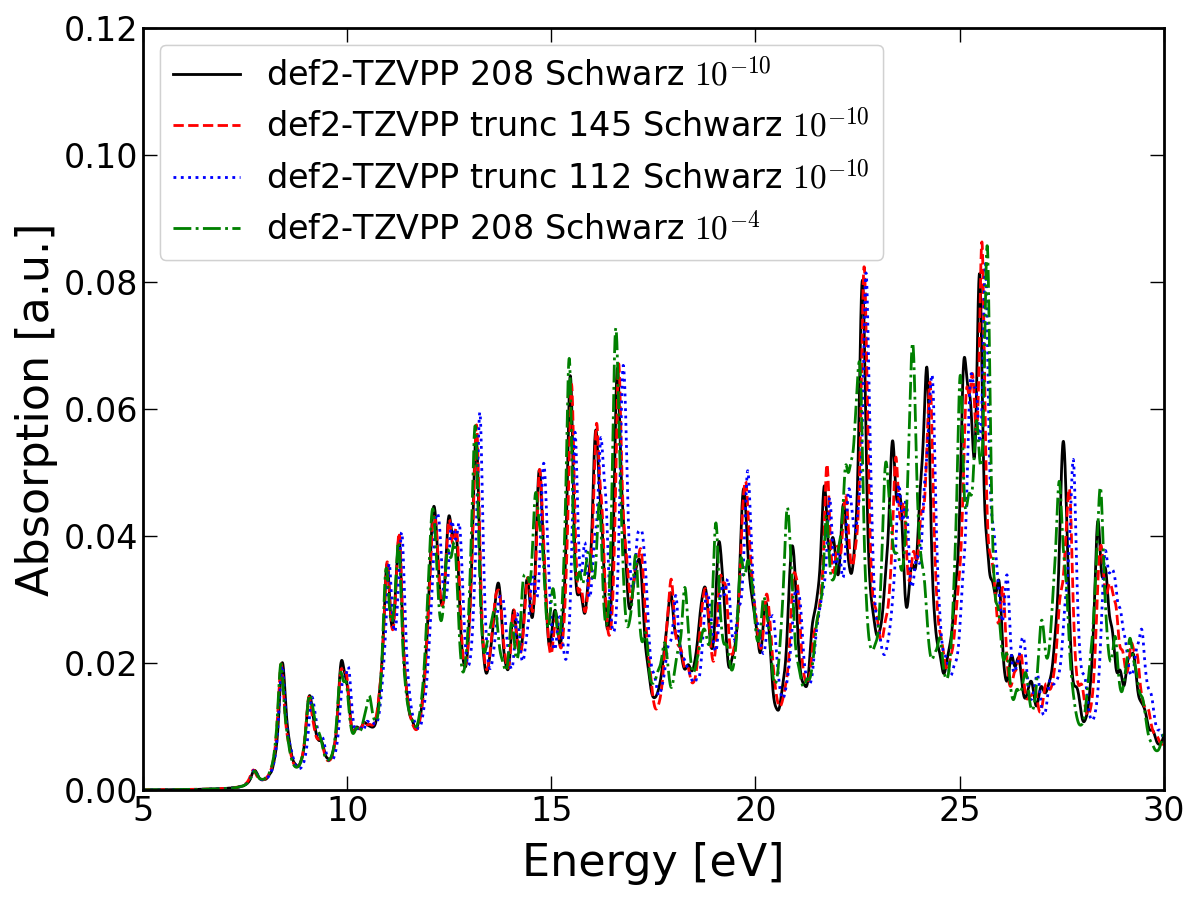

Supplement: Supplementary file 2 — Source Data [file 41467_2022_35694_MOESM2_ESM.zip › figures/c3h6o_spectra_schwarz.png]

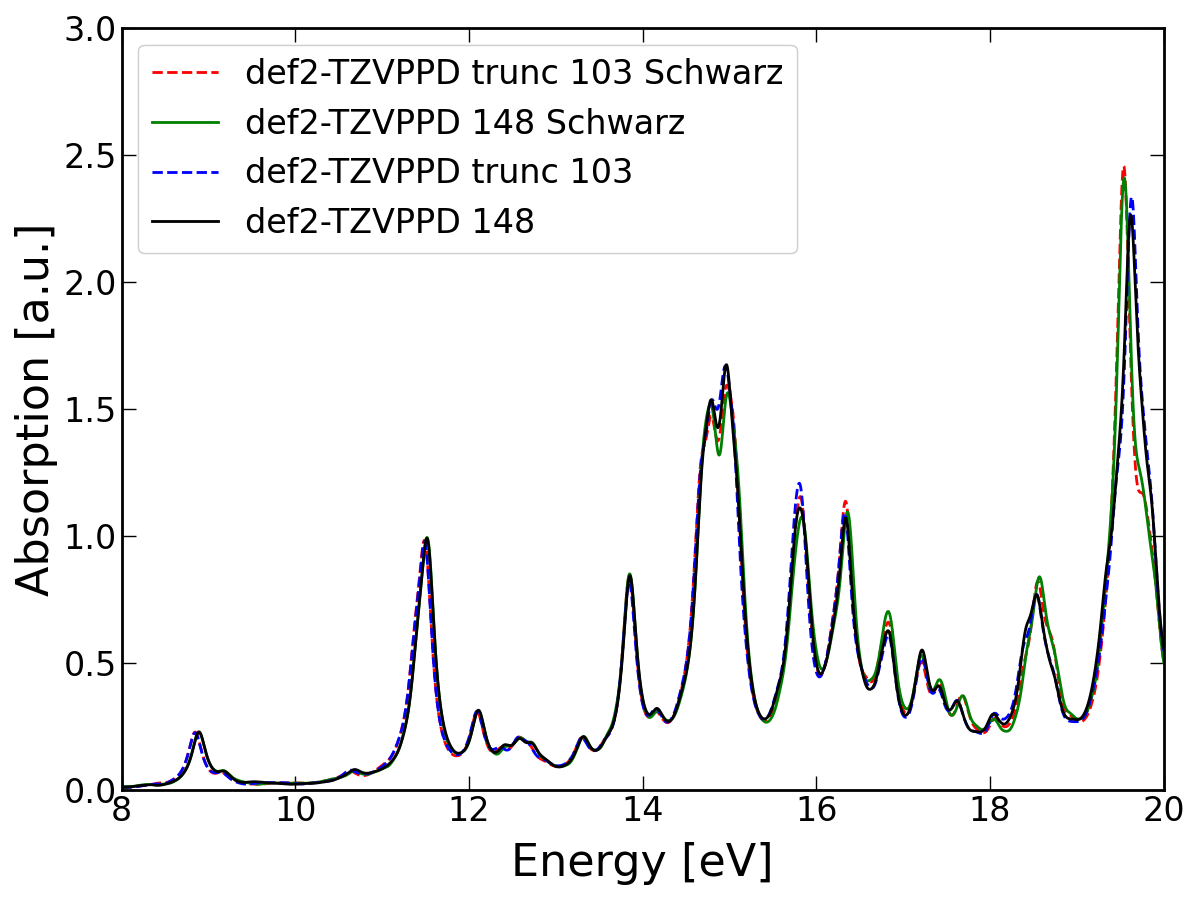

Supplement: Supplementary file 2 — Source Data [file 41467_2022_35694_MOESM2_ESM.zip › figures/h2o_dimer_spectra_schwarz.png]

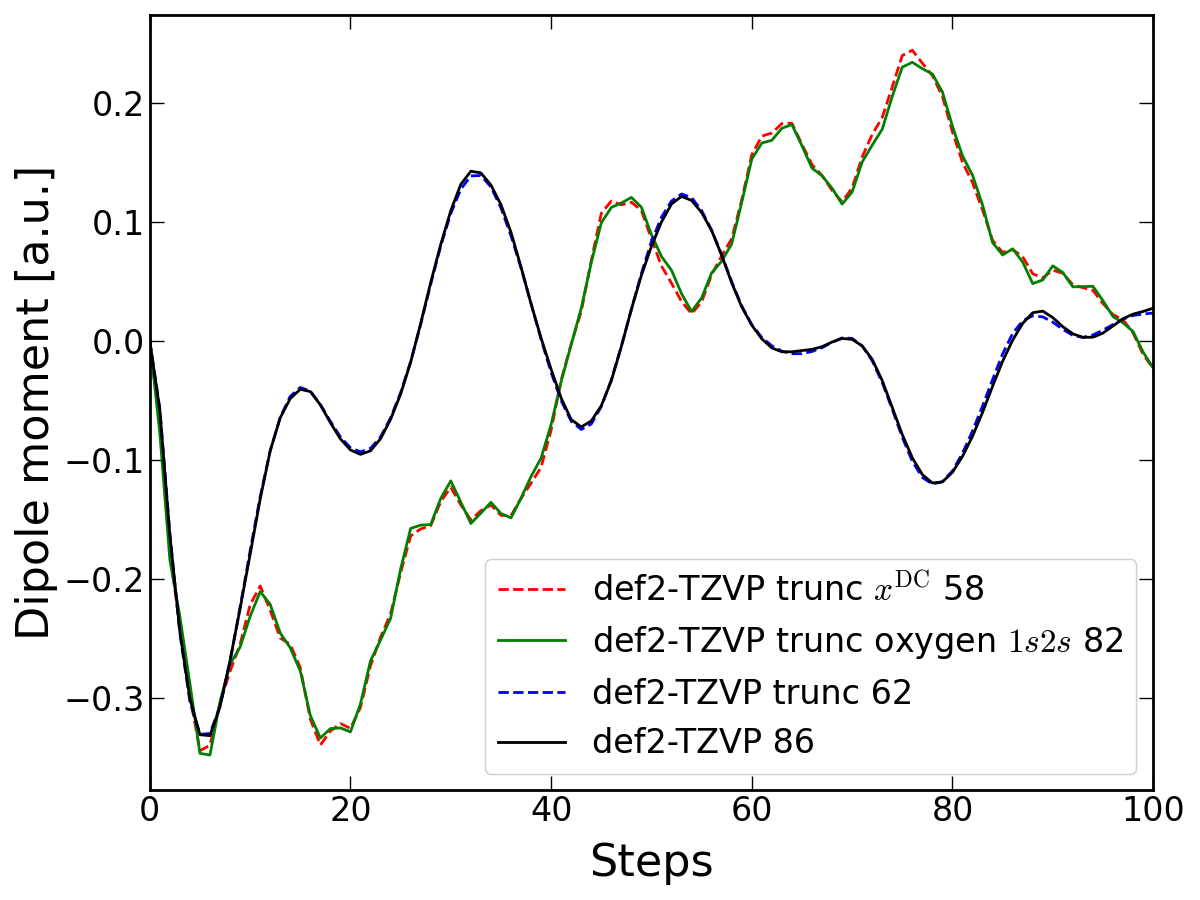

Supplement: Supplementary file 2 — Source Data [file 41467_2022_35694_MOESM2_ESM.zip › figures/h2o_dimer_onlydc_dipole.png]

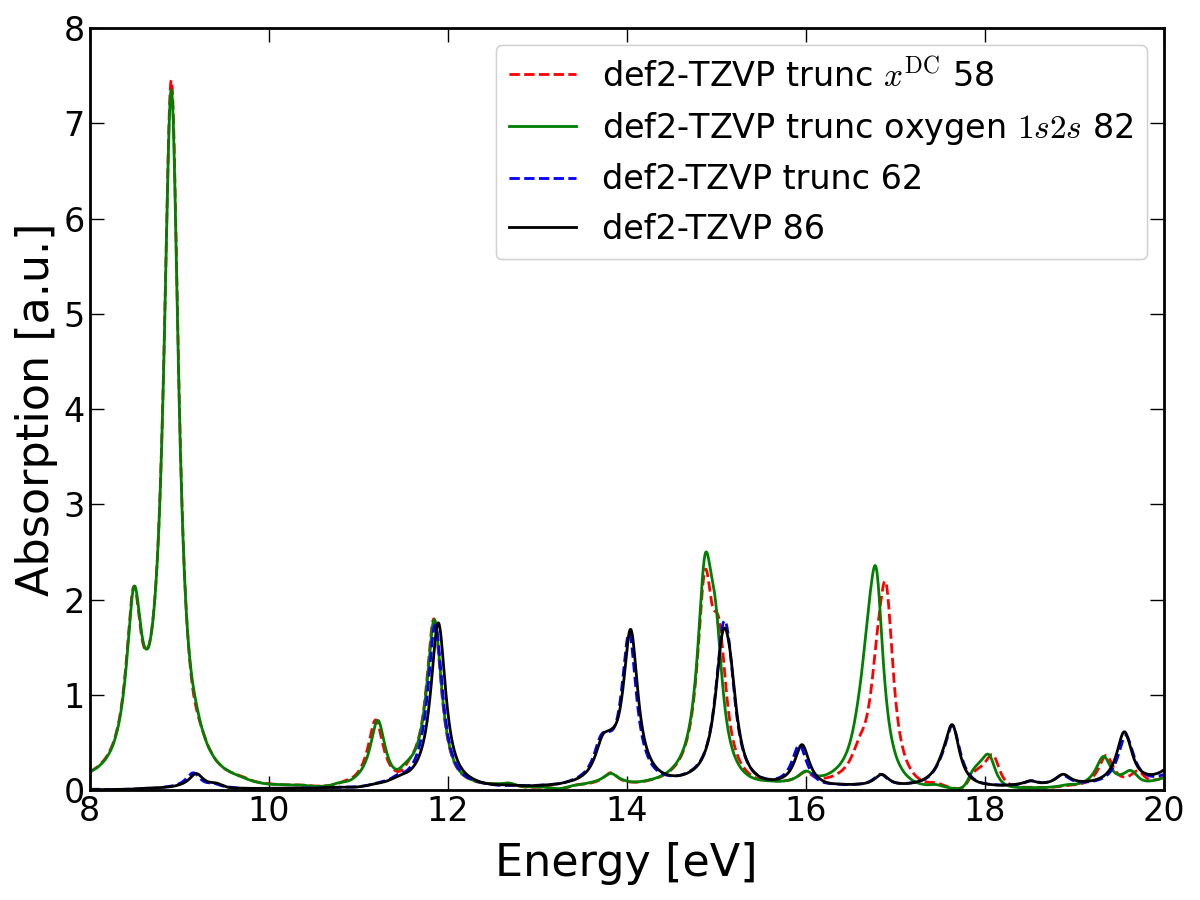

Supplement: Supplementary file 2 — Source Data [file 41467_2022_35694_MOESM2_ESM.zip › figures/h2o_dimer_onlydc_spectra.png]

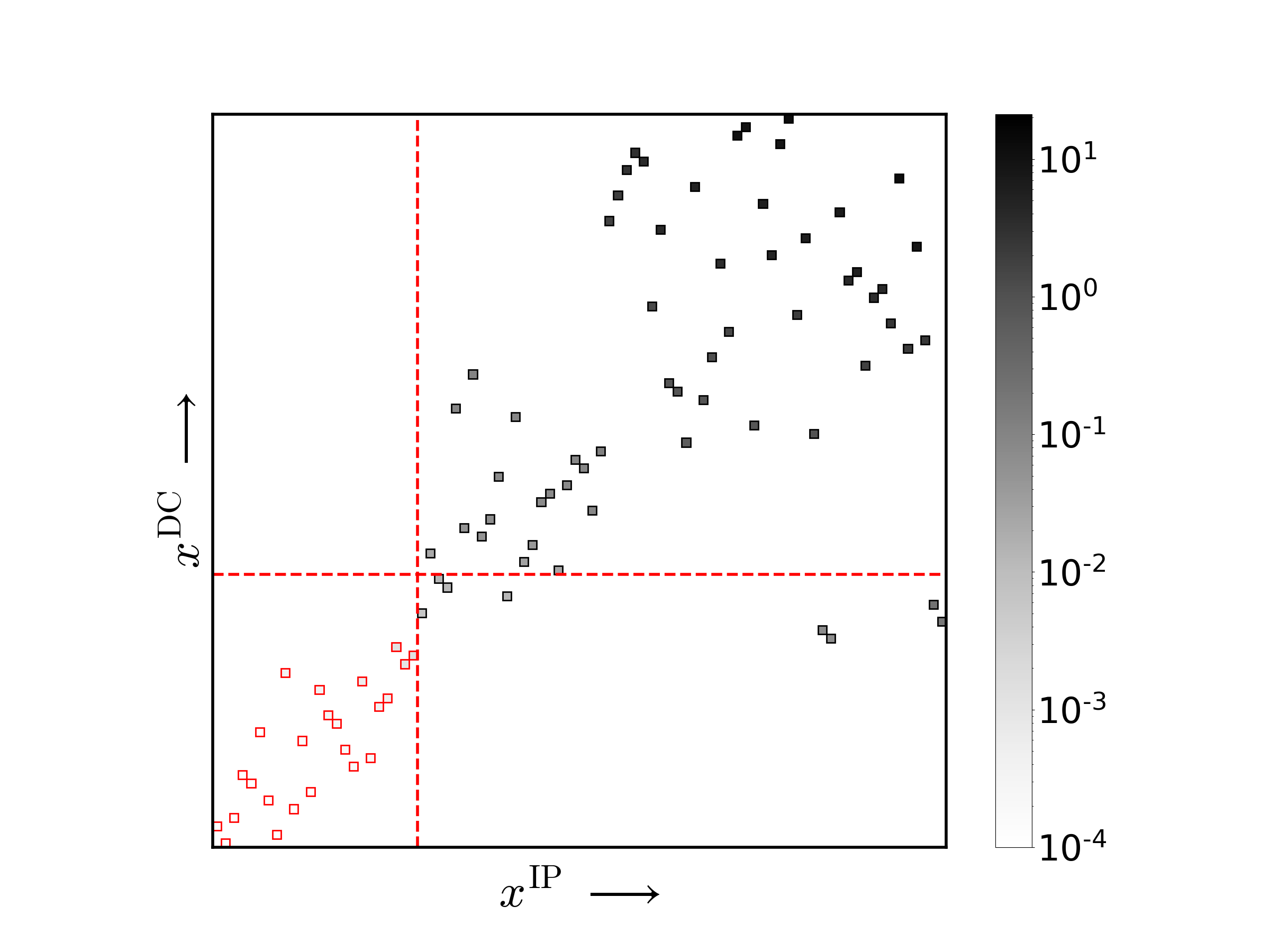

Supplement: Supplementary file 2 — Source Data [file 41467_2022_35694_MOESM2_ESM.zip › figures/h2o_2_steps_4-orb_10k.png]

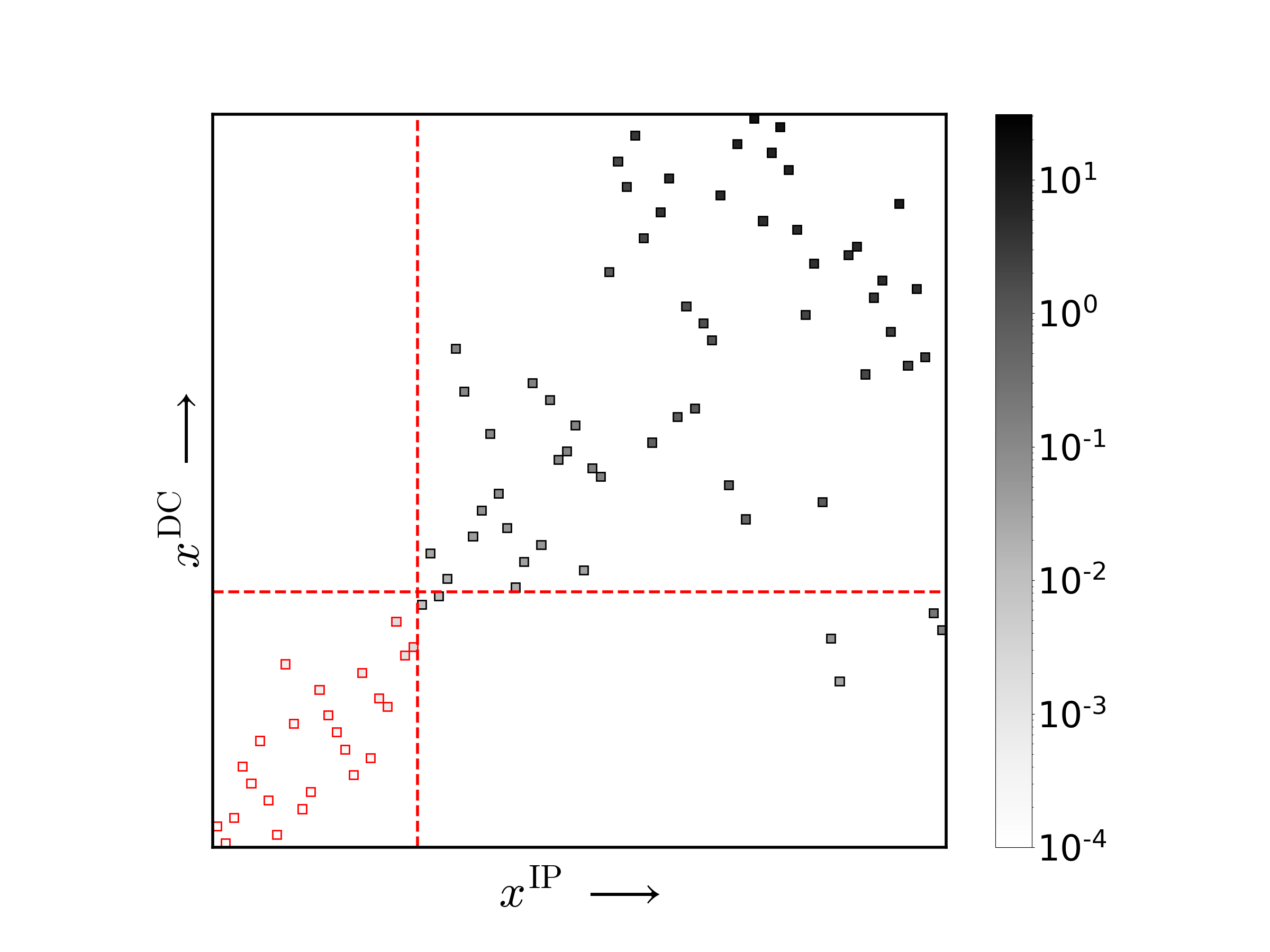

Supplement: Supplementary file 2 — Source Data [file 41467_2022_35694_MOESM2_ESM.zip › figures/h2o_2_steps_4-orb_100.png]

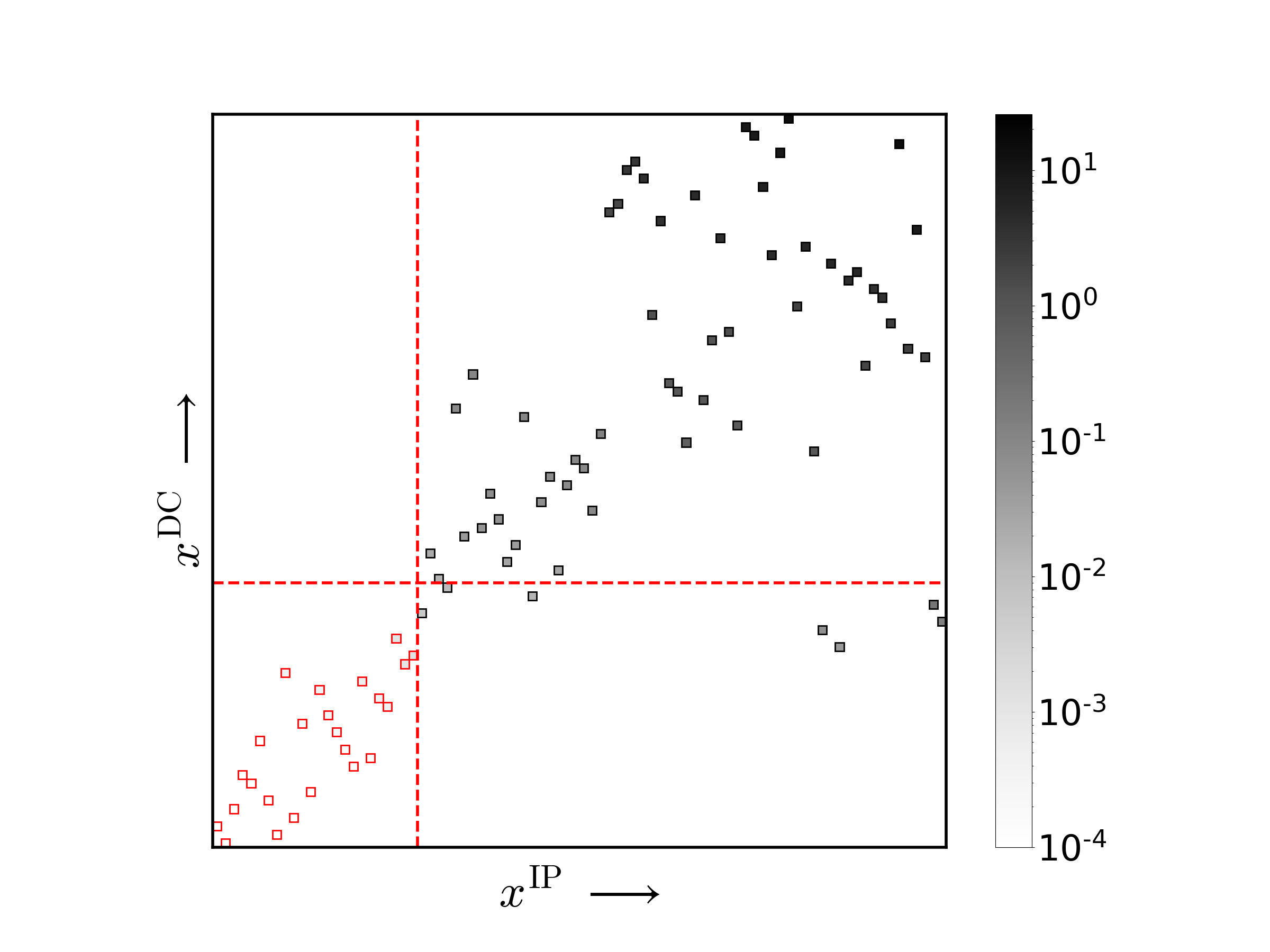

Supplement: Supplementary file 2 — Source Data [file 41467_2022_35694_MOESM2_ESM.zip › figures/h2o_2_steps_4-orb_1k.png]

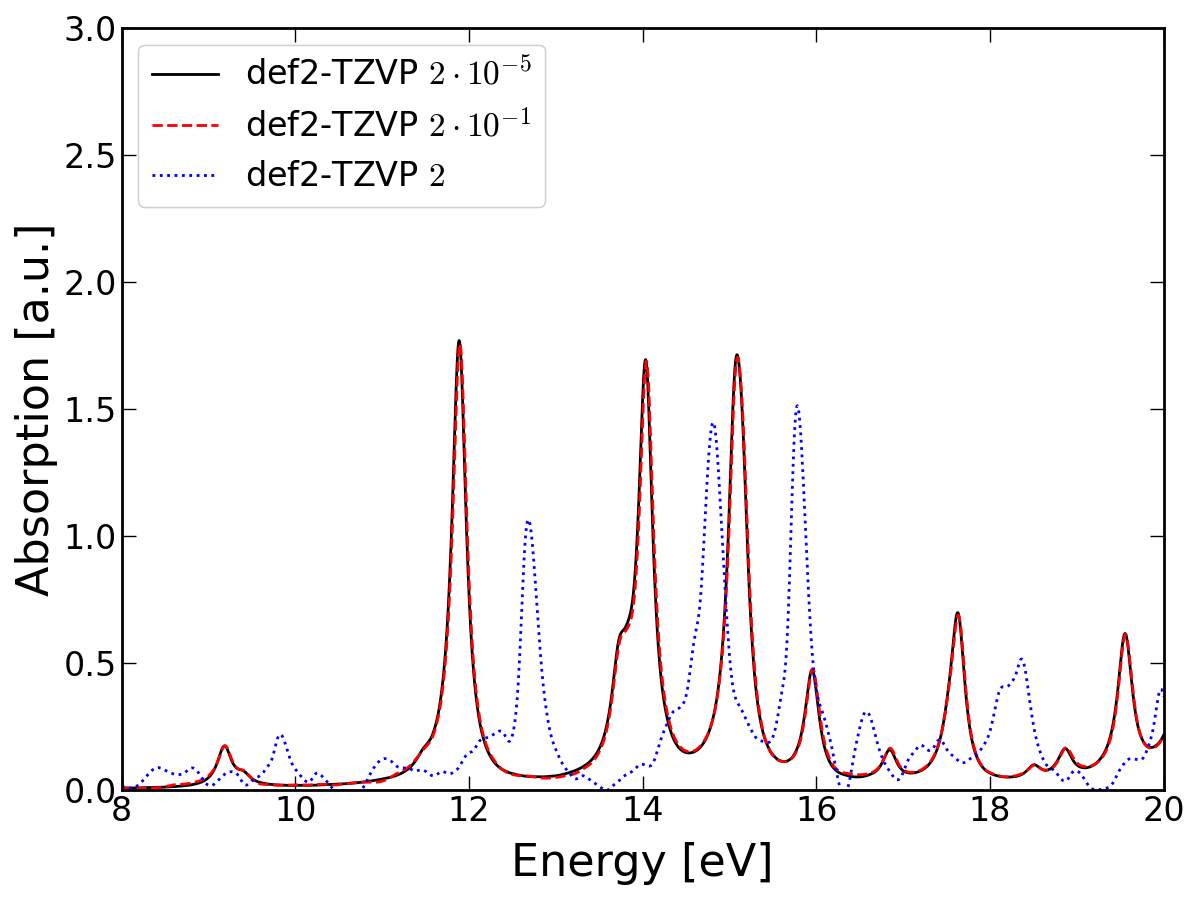

Supplement: Supplementary file 2 — Source Data [file 41467_2022_35694_MOESM2_ESM.zip › figures/spectra_strength.png]

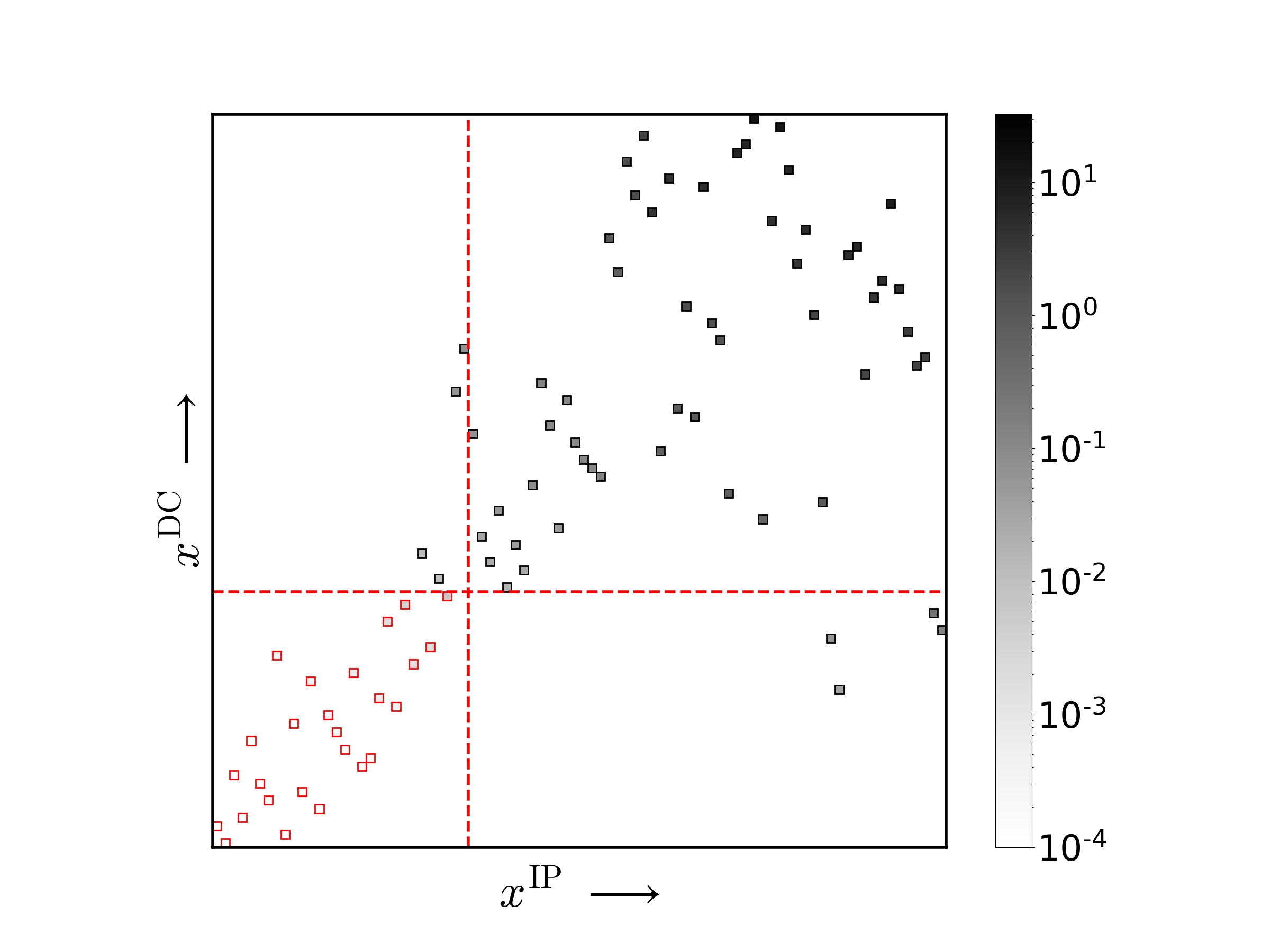

Supplement: Supplementary file 2 — Source Data [file 41467_2022_35694_MOESM2_ESM.zip › figures/h2o_2_strength_4-orb_0.0001.png]

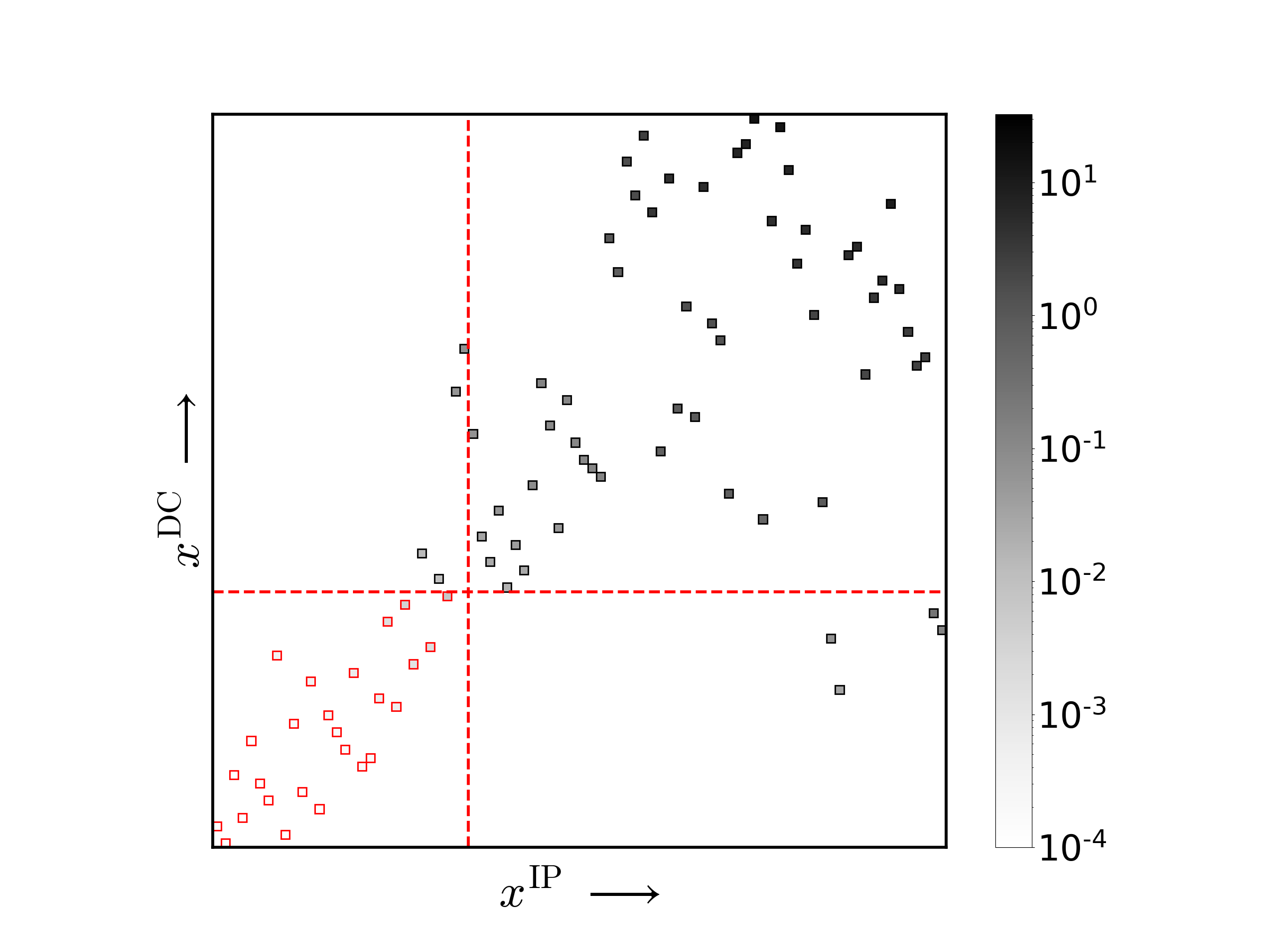

Supplement: Supplementary file 2 — Source Data [file 41467_2022_35694_MOESM2_ESM.zip › figures/h2o_2_strength_4-orb_0.001.png]

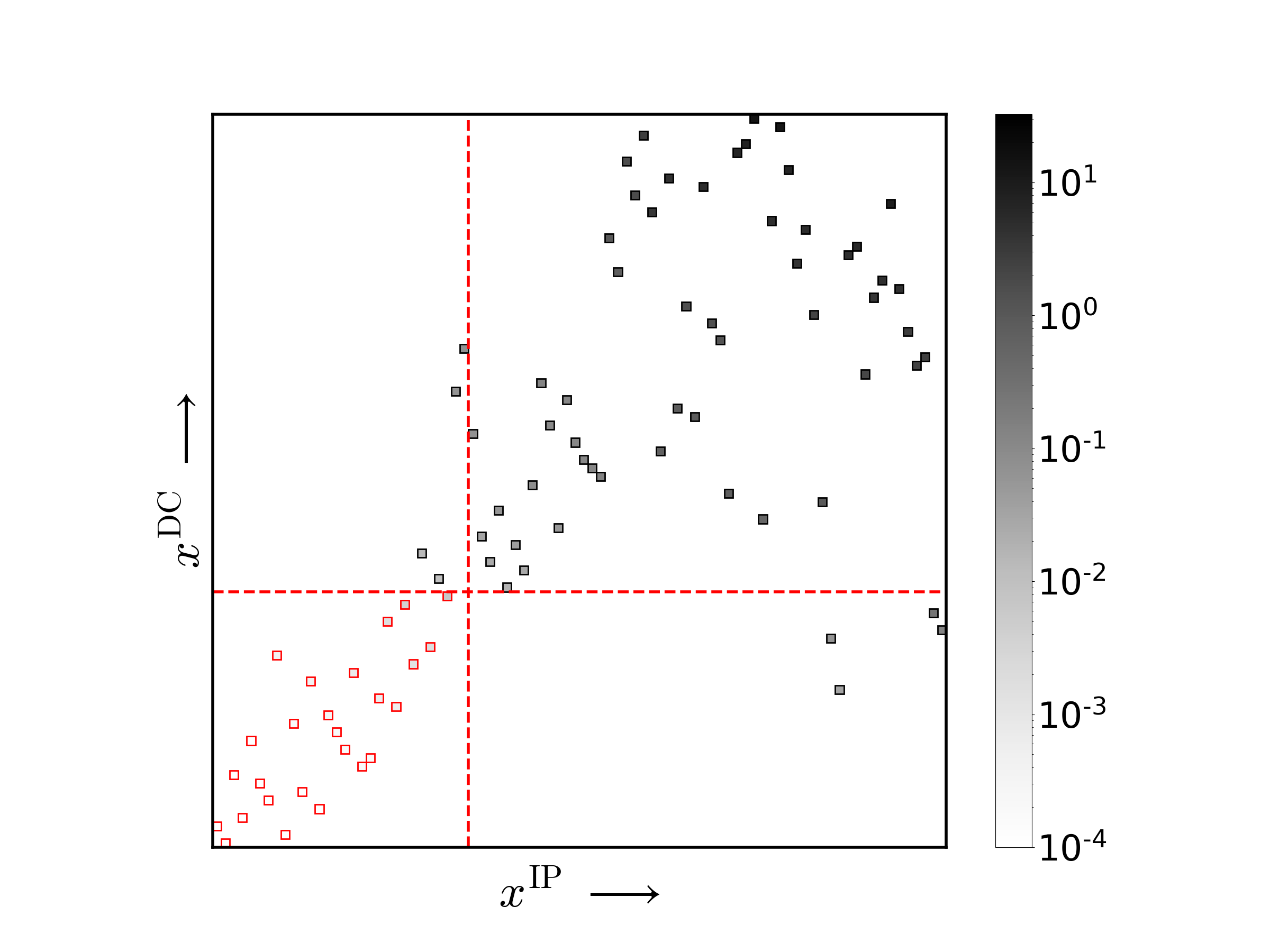

Supplement: Supplementary file 2 — Source Data [file 41467_2022_35694_MOESM2_ESM.zip › figures/h2o_2_strength_4-orb_0.01.png]

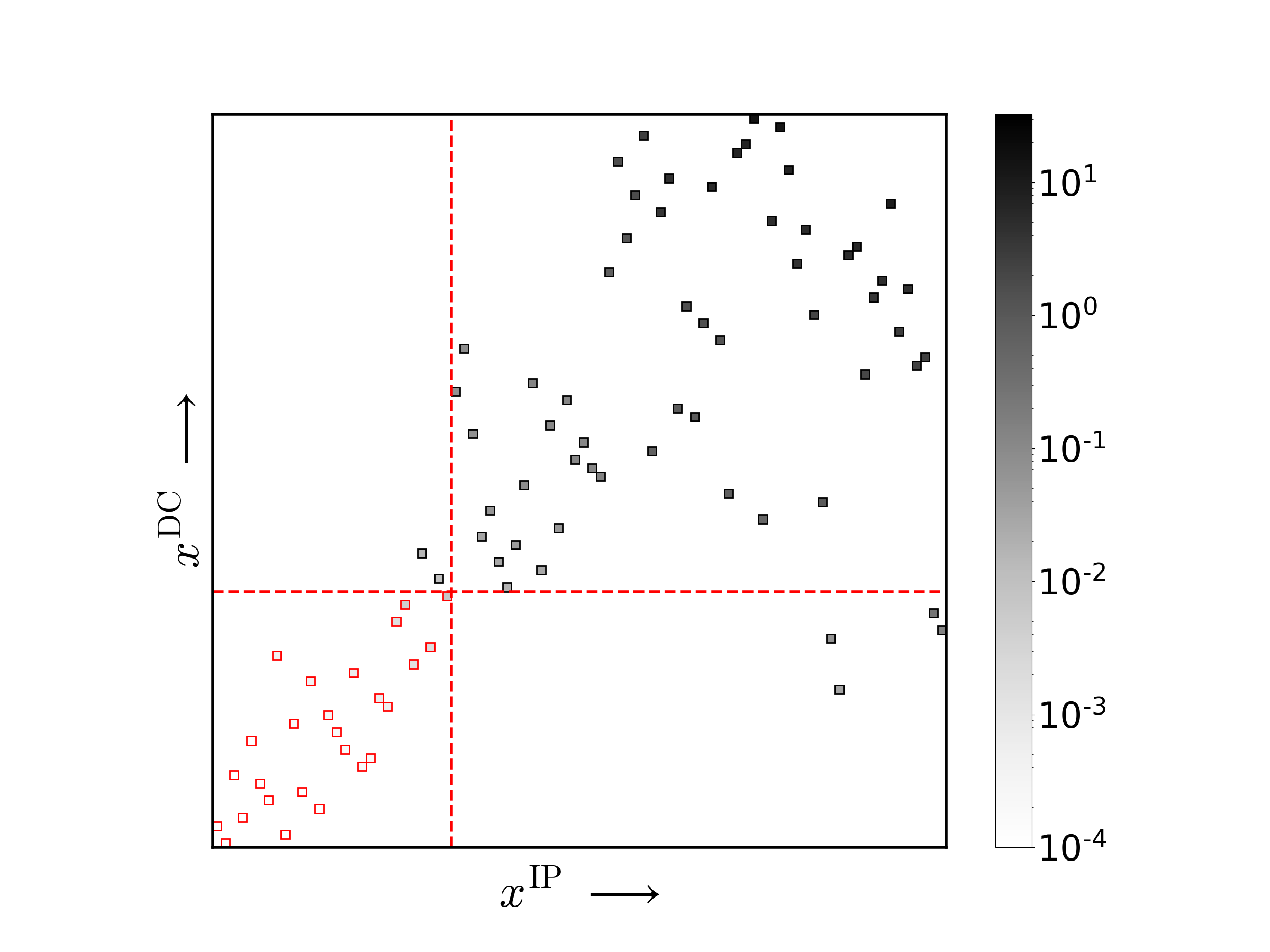

Supplement: Supplementary file 2 — Source Data [file 41467_2022_35694_MOESM2_ESM.zip › figures/h2o_2_strength_4-orb_0.1.png]

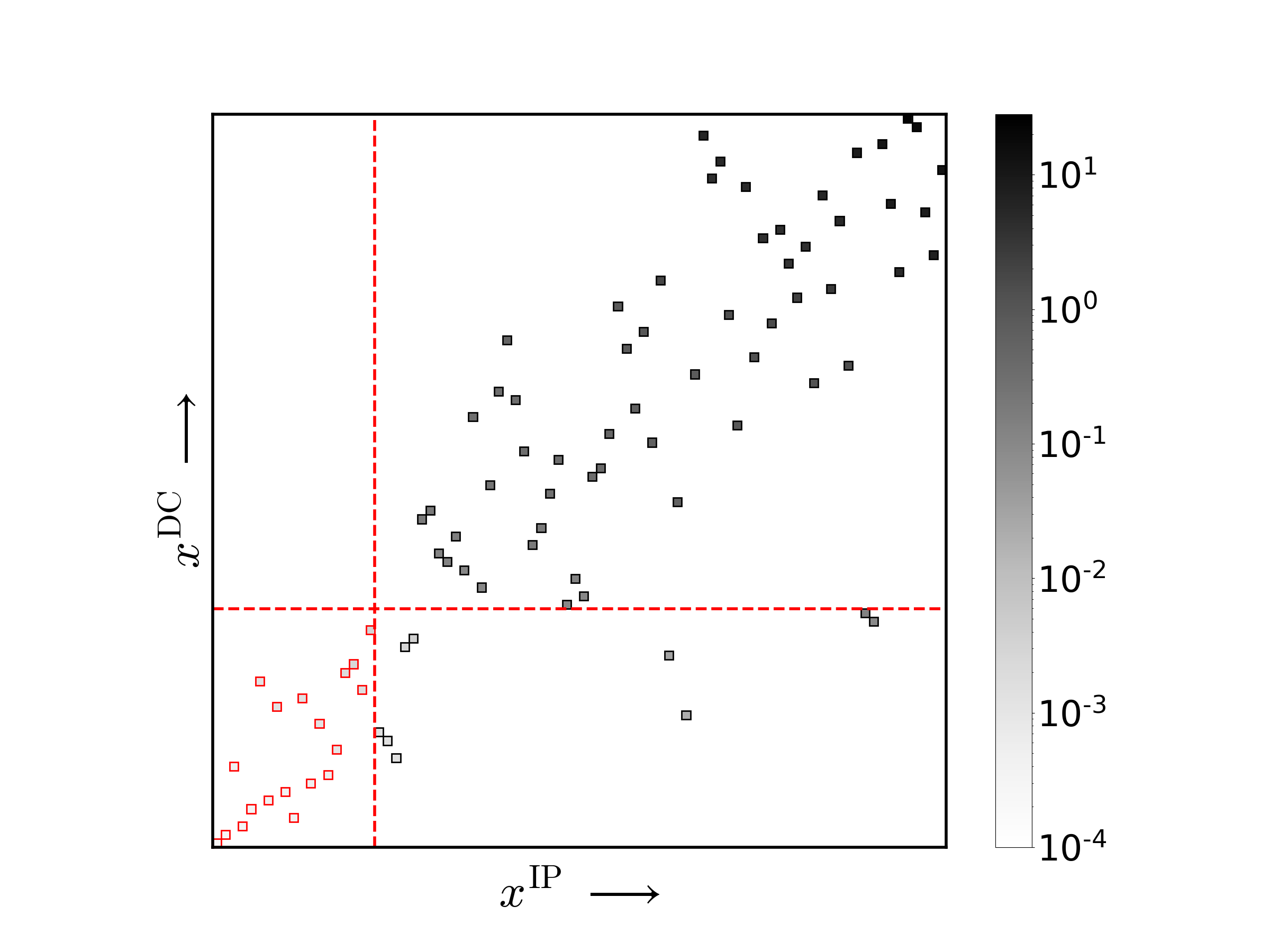

Supplement: Supplementary file 2 — Source Data [file 41467_2022_35694_MOESM2_ESM.zip › figures/h2o_2_strength_4-orb_10.png]
